# Highly specific intracellular ubiquitination of a small molecule

**DOI:** 10.1038/s41589-025-02011-1

**Published:** 2025-08-21

**Authors:** Weicheng Li, Enrique M. Garcia-Rivera, Dylan C. Mitchell, Joel M. Chick, Micah Maetani, Rohit Bhadoria, John M. Knapp, Ryosuke Misu, Geoffrey M. Matthews, Ryosuke Shirasaki, Ricardo de Matos Simoes, Vasanthi Viswanathan, John L. Pulice, Matthew G. Rees, Jennifer A. Roth, Steven P. Gygi, Constantine S. Mitsiades, Cigall Kadoch, Stuart L. Schreiber, Jonathan M. L. Ostrem

**Affiliations:** 1 Department of Medicine, University of California, San Francisco, San Francisco, CA USA; 2 Department of Pediatric Oncology, Dana-Farber Cancer Institute, Harvard Medical School, Boston, MA USA; 3 Broad Institute of MIT and Harvard, Cambridge, MA USA; 4 Department of Cell Biology, Harvard Medical School, Boston, MA USA; 5 Department of Chemistry and Chemical Biology, Harvard University, Cambridge, MA USA; 6 Minase Research Institute, Ono Pharmaceutical Co., Ltd, Osaka, Japan; 7 Department of Medical Oncology, Dana-Farber Cancer Institute, Harvard Medical School, Boston, MA USA; 8 Howard Hughes Medical Institute, Harvard University, Cambridge, MA USA; 9 Present Address: Department of Hematology/Oncology, Teikyo University School of Medicine, Tokyo, Japan

**Keywords:** Mechanism of action, Chemical tools, Chemical modification

## Abstract

Ubiquitin is a small, highly conserved protein that acts as a posttranslational modification in eukaryotes. Ubiquitination of proteins frequently serves as a degradation signal, marking them for disposal by the proteasome. Here we report a novel small molecule from a diversity-oriented synthesis library, BRD1732, that is directly ubiquitinated in cells, resulting in dramatic accumulation of inactive ubiquitin monomers and polyubiquitin chains, which causes broad inhibition of the ubiquitin–proteasome system. Ubiquitination of BRD1732 and its associated cytotoxicity are stereospecific and dependent on two homologous E3 ubiquitin ligases, RNF19A and RNF19B, and their shared E2 conjugating enzyme, UBE2L3. Our finding opens the possibility for indirect ubiquitination of a target through a ubiquitinated bifunctional small molecule and more broadly raises the potential for posttranslational modification in *trans*.

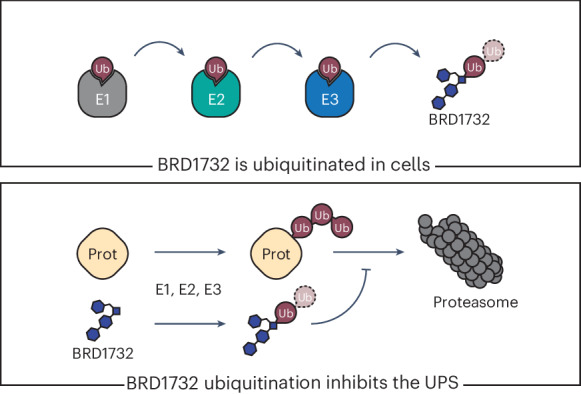

## Abstract (web summary)

Li et al. discovered that the cytotoxic synthetic small molecule BRD1732 is directly ubiquitinated in cells. Ubiquitination of BRD1732 is E3 ligase dependent and leads to inhibition of proteasomal degradation.

## Main

Until recently, the bulk of small-molecule development efforts largely focused on planar molecules having high *sp*^2^ hybridization^1^, which might not optimally capitalize on the three-dimensional complexity or stereospecificity of biological macromolecules. Diversity-oriented synthesis (DOS) produces small-molecule libraries whose members have diverse stereochemistry and three-dimensional skeletons. This has yielded novel chemical probes with diverse targets and functions^2,3^. An innovative use of these libraries is to identify small molecules with entirely new modes of action within the cell through mechanistic studies of molecules with interesting phenotypes.

Mechanism-of-action (MOA) studies of small molecules have repeatedly led to important biological advances and entirely new avenues for drug development. MOA studies of the immunosuppressant natural products cyclosporin A and FK506 revealed for the first time that small molecules can function as molecular glues by inducing protein–protein interactions^4^. Mechanistic studies revealed that the myeloma drug lenalidomide functions as a molecular glue degrader, co-opting the ubiquitin–proteasome system (UPS) and promoting ubiquitination and degradation of two zinc finger transcription factors, IKZF1 and IKZF3, by inducing their association with the E3 ubiquitin ligase cereblon^5,6^. The discovery of molecular glue degraders has led to extensive efforts to understand and harness the UPS for therapeutic applications. While many tools and drugs have been developed to modulate the UPS, none act at the level of ubiquitin itself.

The ubiquitination cascade consists of activation of ubiquitin by an E1 enzyme, transfer to an E2 conjugating enzyme and ultimately conjugation of ubiquitin to a substrate in a reaction mediated by an E3 ligase. Until recently, ubiquitination was thought to occur exclusively on protein substrates. However, recent studies have demonstrated ubiquitination of some nonprotein substrates, including lipids and sugars^7,8^. Here, we report identification of a synthetic small molecule, BRD1732, that is directly ubiquitinated in cells, dependent on a pair of E3 ubiquitin ligases, RNF19A and RNF19B, and their shared E2 conjugating enzyme, UBE2L3. Ubiquitination of BRD1732 results in a covalent linkage between the C terminus of ubiquitin and a secondary amine on BRD1732. The resulting ubiquitin–BRD1732 conjugate accumulates dramatically in cells. In addition, we demonstrate accumulation of diubiquitin–BRD1732 conjugates assembled through noncanonical K27 ubiquitin–ubiquitin linkage, a rare and poorly characterized linkage type^9,10^. Likely through pleotropic effects on the UPS, ubiquitination of BRD1732 leads to inhibition of ubiquitin-dependent proteasomal degradation.

## Results

### BRD1732 is a stereospecific cytotoxin dependent on the E3 ligases RNF19A and RNF19B

**Fig. 1:**
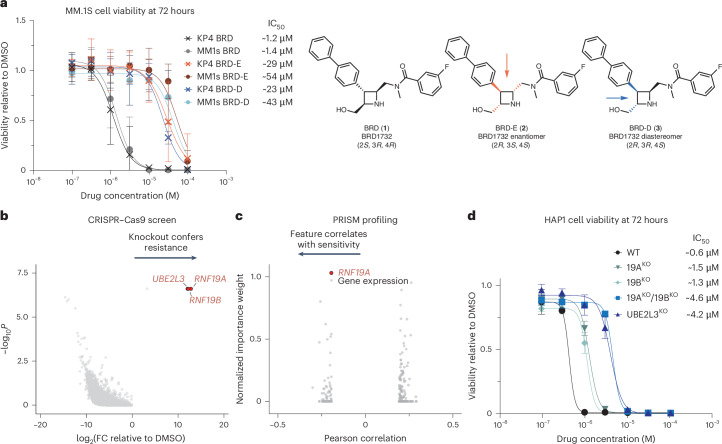
BRD1732 is a stereospecific cytotoxin dependent on the E3 ligases RNF19A and RNF19B. **a**, Structures of BRD1732 (**1**), BRD-E (**2**) and BRD-D (**3**) and cell viability after 72-h drug treatment in KP4 and MM.1S cells. Data are shown as mean ± s.d. of *n* = 6 biological replicates (*n* = 9 for MM.1S BRD and BRD-E) representing at least 2 independent experiments. **b**, Genome-wide CRISPR–Cas9 KO screen in MM.1S^Cas9^ multiple myeloma cells treated with 5 µM BRD1732 or DMSO. Top hit genes enriched in BRD1732-treated cells are highlighted in red. One-sided *P* values were calculated using the MAGeCK algorithm (*n* = 3 biological replicates). **c**, PRISM screen of pooled, barcoded cancer cell lines treated with 5 µM BRD1732 showing sensitivity feature Pearson correlation with area under the curve. The importance weight was calculated using enet. **d**, Cell viability after 72-h drug treatment of BRD1732 in CRISPR–Cas9 KO cell lines with indicated genotypes. Data are shown as mean ± s.d. of *n* = 6 biological replicates (*n* = 9 for WT and DKO) representing at least 2 independent experiments.

We identified the azetidine scaffold exemplified by BRD1732 (**1**) from a DOS library as having stereospecific activity across multiple phenotypic screens. BRD1732 is broadly cytotoxic in cancer and immortalized cell lines, irrespective of lineage, with a half-maximal inhibitory concentration (IC_50_) of approximately 1–3 µM and this effect is exclusive to the (2*S*,3*R*,4*R*) stereoisomer, meaning that altering one or more stereocenters on BRD1732 dramatically reduces activity (Fig. 1a and Supplementary Fig. 1)^11^. Compared to BRD1732, both its enantiomer BRD-E (**2**) and a diastereomer BRD-D (**3**) are 10–40-fold less potent in viability assays across multiple cell lines. Our finding that activity depends on the three-dimensional orientation of the substituents on BRD1732 suggested to us that this molecule acts through direct interaction with one or more macromolecules.

To identify the molecular machinery required for the cytotoxicity of BRD1732, we performed a genome-wide CRISPR–Cas9 resistance screen (Fig. 1b and Supplementary Data 1). Single guide RNAs (sgRNAs) targeting *RNF19A*, *RNF19B* and *UBE2L3* were significantly enriched following BRD1732 treatment, suggesting that loss of these genes confers resistance. RNF19A and RNF19B are homologous RING-in-between-RING (RBR) E3 ubiquitin ligases and UBE2L3 is an E2 ubiquitin-conjugating enzyme responsible for charging the RNF19 proteins with ubiquitin^12^. Using the PRISM platform^13^ to profile the dependency features of BRD1732 across ~580 cancer cell lines, we identified *RNF19A* expression as a feature strongly associated with sensitivity to BRD1732 (Fig. 1c and Supplementary Data 2). Mean proliferation IC_50_ was 1.3 µM (95% confidence interval: 0.21–4.3 µM) across all cell lines. Taken together, the bidirectional findings from CRISPR screening and PRISM profiling strongly support a central role of RNF19A/B in the cytotoxicity of BRD1732. To validate these findings, we used CRISPR–Cas9 to generate single-knockout (KO) (RNF19A, RNF19B and UBE2L3) and double-KO (DKO) (RNF19A and RNF19B) clones from the haploid cell line HAP1. KO of either RNF19A or RNF19B resulted in a 2–3-fold reduction in potency of BRD1732 in viability assays (Fig. 1d and Supplementary Fig. 2). KO of UBE2L3 or DKO of RNF19A and RNF19B resulted in a ~10-fold reduction in potency of BRD1732.

### BRD1732 is directly ubiquitinated in cells

**Fig. 2:**
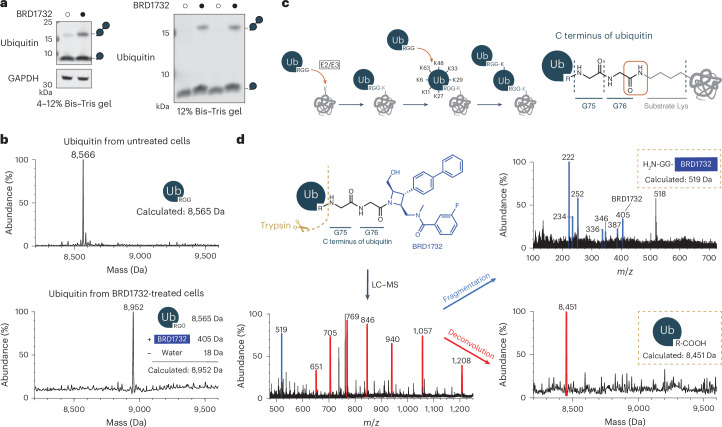
BRD1732 is directly ubiquitinated in cells. **a**, HEK293T cells were treated with DMSO or BRD1732 for 6 h and ubiquitin levels were analyzed by immunoblot following separation on 4–12% Bis–Tris or 12% polyacrylamide gel. Images are representative of three and two independent experiments, respectively. **b**, Deconvoluted mass spectra from intact protein LC–MS of ubiquitin purified from untreated and BRD1732-treated cells. **c**, Schematic representations showing conjugation of ubiquitin C-terminal glycine to ε-amino group of a lysine on the target substrate. **d**, Left: schematic representation of trypsin digestion of ubiquitin–BRD1732 conjugate with intact protein LC–MS spectrum of trypsin-digested ubiquitin from BRD1732-treated ubiquitin. Right: LC–MS/MS spectrum from fragmentation of the 519 ion and spectrum from deconvolution of the envelope of peaks highlighted in red. Images in **b** and **d** are representatives of three independent experiments. Source data

While further interrogating the effects of BRD1732 on the UPS, we made the unexpected discovery that treatment of cells with BRD1732 causes accumulation of monoubiquitin and diubiquitin species along with a very small but reproducible gel shift for monoubiquitin (Fig. 2a). This effect is even more prominent on 12% Bis–Tris polyacrylamide gel. We reasoned that this could indicate a change in posttranslational modification of ubiquitin or conjugation of ubiquitin to a small molecule or metabolite. BRD1732-induced accumulation of ubiquitin species is even more pronounced in cells overexpressing RNF19A (Supplementary Fig. 3); thus, we purified endogenous ubiquitin from RNF19A-overexpressing Expi293F cells either untreated or treated with 10 µM BRD1732 for 6 h. We then purified these ubiquitin samples by cation exchange followed by size-exclusion chromatography (SEC) and analyzed them by liquid chromatography–mass spectrometry (LC–MS) (Fig. 2b). The mass of endogenous ubiquitin from untreated cells matches the calculated mass for unmodified ubiquitin (8,565 Da). However, the mass of ubiquitin from BRD1732-treated cells is 387 Da greater, which exactly matches the predicted mass of a covalent adduct between ubiquitin and BRD1732 with loss of water (8,952 Da).

Ubiquitin is transferred to substrate lysine residues by formation of an isopeptide bond between the C terminus of ubiquitin and the ε-amine of lysine resulting in loss of a water molecule (Fig. 2c)^14^. Given that BRD1732 contains a free aliphatic amine like lysine, we suspected that BRD1732 was mimicking a substrate protein and undergoing direct ubiquitination (Fig. 2d). To further characterize the ubiquitin conjugate formed in cells, we performed trypsin digestion under nondenaturing conditions. Digestion of unmodified ubiquitin under these conditions results in a single cleavage after R74 to yield ubiquitin(1–74), as previously reported^15^, with a mass of 8,452 Da by LC–MS (Supplementary Fig. 4). Similar trypsin digestion of the BRD1732-induced ubiquitin conjugate yielded ubiquitin(1–74) and a new fragment ion at *m*/*z* = 519, which corresponds to the predicted mass of Gly-Gly-BRD1732 ([M + H]^+^ = 519 Da). Further fragmentation of this 519 ion yielded BRD1732 ([M + H]^+^ = 405) and its expected fragmentation ions (Fig. 2d).

### BRD1732 is ubiquitinated on a secondary amine

**Fig. 3:**
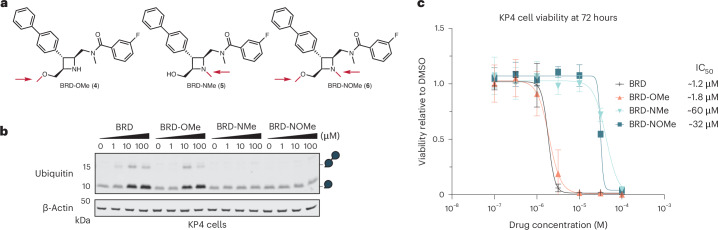
BRD1732 is ubiquitinated on the azetidine secondary amine. **a**, Structures of methylated BRD1732 analogs, including *O*-methyl analog BRD-OMe (**4**), *N*-methyl analog BRD-NMe (**5**) and *N*,*O*-bis(methyl) BRD1732 analog BRD-NOMe (**6**). Sites of methylation are indicated with arrows. **b**, Immunoblots of lysate from KP4 cells treated with BRD1732, BRD-OMe, BRD-NMe and BRD-NOMe for 6 h at 0, 1, 10 and 100 µM, representative of three independent experiments. **c**, Cell viability after 72-h drug treatment with BRD1732, BRD-OMe, BRD-NMe and BRD-NOMe in KP4 cells. Data are shown as mean ± s.d. of *n* = 9 biological replicates representing three independent experiments. Source data

The only two potential sites of ubiquitination on BRD1732 are the primary alcohol or the azetidine nitrogen, a secondary amine. To identify the site of ubiquitination, we synthesized three analogs of BRD1732 in which one or both of these sites was modified by a methyl group, chemically inactivating them (Fig. 3a). As we expected, methylation of the primary alcohol alone BRD-OMe (**4**) had no effect on formation of ubiquitin conjugates in cells (Fig. 3b) and no notable effect on the IC_50_ in proliferation assays (Fig. 3c). Conversely, methylation of the azetidine nitrogen alone BRD-NMe (**5**) or in combination with the primary alcohol BRD-NOMe (**6**) completely prevented accumulation of ubiquitin–BRD1732 conjugates and increased the IC_50_ to >30 µM. Taken together, these results demonstrate that BRD1732 is directly ubiquitinated in cells by formation of an amide bond between the ubiquitin C terminus and the azetidine secondary amine.

### Ubiquitination of BRD1732 is stereospecific and dependent on UBE2L3 and RNF19

**Fig. 4:**
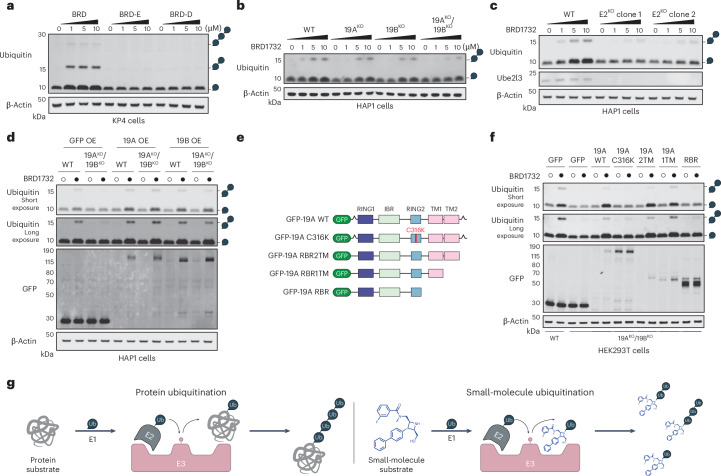
Formation of BRD1732–ubiquitin conjugates is dependent on UBE2L3 and RNF19. **a**, Immunoblots of lysate from KP4 pancreatic cancer cells treated with BRD1732, BRD-E or BRD-D for 6 h at 0, 1, 5 and 10 µM. Ubiquitin chain symbols indicate the expected molecular weight for unanchored ubiquitin chains of various lengths. **b**, Immunoblot of HAP1 WT and *RNF19A* and *RNF19B* single-KO and DKO cells treated for 6 h with 5 µM BRD1732 or DMSO. **c**, Immunoblot of HAP1 WT or *UBE2L3* KO cells treated for 6 h with 5 µM BRD1732 or DMSO. **d**, Immunoblot of HAP1 WT and *RNF19A/B*-DKO cells transfected to express GFP, RNF19A or RNF19B then treated for 6 h with 5 µM BRD1732 or DMSO. **e**, Schematic diagram of RNF19A mutants and truncation constructs. **f**, Immunoblots of HEK293T WT or DKO cells transfected with the indicated expression constructs then treated for 6 h with 5 µM BRD1732 or DMSO. **g**, Schematic representation of protein substrate ubiquitination by ubiquitin ligases along with our proposed mechanism of small-molecule ubiquitination. Immunoblots in **a**–**d** and **f** are representative of three independent experiments. Source data

BRD1732–ubiquitin conjugate accumulation is concentration dependent and stereospecific (Fig. 4a,b). To understand whether this accumulation of ubiquitin species is dependent on RNF19 proteins, we performed immunoblot analysis in wild-type (WT) and KO HAP1 cell lines (Fig. 4b,c). KO of either *RNF19A* or *RNF19B* decreased formation of these ubiquitin species and DKO of these genes had an even more dramatic effect (Fig. 4b). Similarly, KO of *UBE2L3* greatly reduced the accumulation of both monoubiquitin and diubiquitin species (Fig. 4c). Exogenous stable overexpression of GFP–RNF19A or GFP–RNF19B in DKO (19A^KO^;19B^KO^) HAP1 or DKO HEK293T cells resulted in recovery of BRD1732 ubiquitination (Fig. 4d and Supplementary Fig. 5). We also note a dramatic increase in RNF19A and RNF19B levels upon treatment with BRD1732, consistent with stabilization of these proteins by BRD1732. RBR E3 ligases require transfer of ubiquitin from the E2 enzyme to a catalytic cysteine in the E3 RING2 domain before transfer to a substrate^16^. Importantly, BRD1732-induced accumulation of ubiquitin requires the RBR core of RNF19A (RING1-IBR-RING2), including the catalytic C316 and at least one of its two transmembrane domains but does not require the N-terminal extension or the C-terminal regulatory domain as demonstrated by exogenous overexpression of mutants of RNF19A in 19A^KO^;19B^KO^ HEK293T cells (Fig. 4f). Based on these findings, we conclude that BRD1732 is ubiquitinated in cells in a manner that is dependent upon the E3 ligases RNF19A and RNF19B and their shared E2 enzyme UBE2L3 (Fig. 4g).

### BRD1732 disrupts the UPS at multiple pathway nodes

**Fig. 5:**
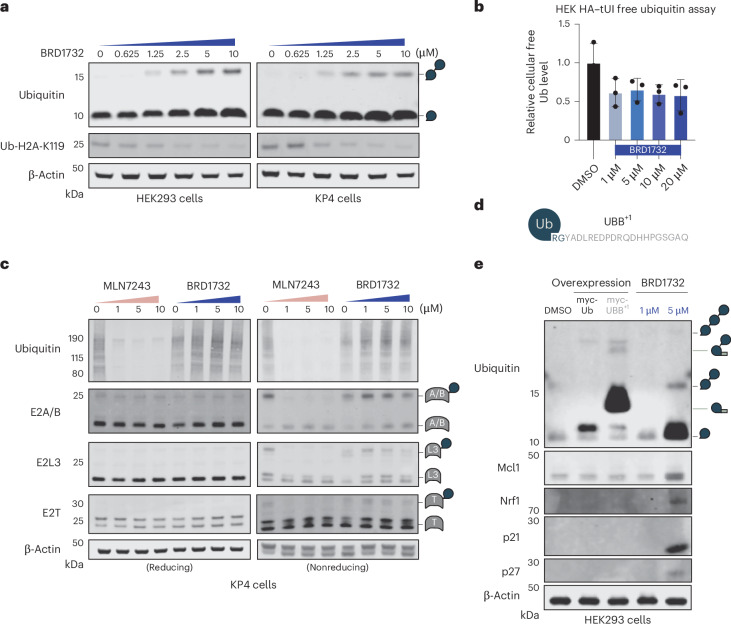
BRD1732 has pleotropic effects on the UPS. **a**, Immunoblots following treatment of HEK293T or KP4 cells with BRD1732 at indicated concentrations for 24 h. **b**, Flow cytometry of HEK293T cells treated with BRD1732 at indicated concentrations for 6 h, stained with free ubiquitin binder HA–tUI followed by Alexa Fluor 750-conjugated anti-HA antibody. Data are presented as mean ± s.e.m. for three biological replicates. **c**, Immunoblots following treatment of KP4 cells with indicated concentrations of BRD1732 or MLN7243 for 6 h. **d**, Schematic representation of UBB^+1^ with 20-aa C-terminal extension. **e**, HEK293T cells were treated with BRD1732 for 24 h or were transfected with myc-tagged ubiquitin or myc-tagged UBB^+1^. Accumulation of polyubiquitin and stabilization of short-half-life proteins were analyzed by immunoblotting. Immunoblots in **a**, **c** and **e** are representative of three independent experiments. Source data

Following purification of endogenous ubiquitin from BRD1732-treated cells, we exclusively detect the ubiquitin–BRD1732 conjugate by LC–MS. Therefore, we wondered whether BRD1732 treatment might interfere with ubiquitin homeostasis in the cell. Indeed, treatment of WT HEK293T or KP4 cells with BRD1732 caused concentration-dependent loss of histone H2A-K119 ubiquitination, potentially consistent with depletion of nuclear ubiquitin (Fig. 5a). Because loss of H2A-K119 ubiquitination can also indicate more general ubiquitin-related stress, we directly quantified free monoubiquitin by flow cytometry staining with HA–tUI (Fig. 5b and Supplementary Fig. 6)^17^. HA–tUI is a tagged synthetic protein designed to bind free ubiquitin with high affinity (dissociation constant 66 pM) and high specificity. Using this method, HEK293T cells treated with BRD1732 showed a slight decrease in free ubiquitin compared to DMSO-treated cells. However, we note that this effect does not show a clear dose response. In addition, exogenous overexpression of ubiquitin does not significantly alter the IC_50_ for BRD1732 in cell viability assays (Supplementary Fig. 7). Thus, despite noting dramatic accumulation of ubiquitin–BRD1732 conjugates in cells, we do not see evidence that cellular ubiquitin stores are depleted upon treatment with BRD1732 and exogenous overexpression of ubiquitin does not confer resistance to BRD1732. Taken together these effects do not adequately explain the toxicity of BRD1732.

The adenosine monophosphate mimetic ubiquitin-activating enzyme (UAE) inhibitor MLN7243 conjugates to the C terminus of ubiquitin within the UAE active site^18^. The resulting ubiquitin–MLN7243 conjugate potently inhibits the UAE itself. Given the broad requirement of UAE for ubiquitination within the cell, MLN7243 treatment results in depletion of all conjugated and unconjugated ubiquitin species with the exception of free monoubiquitin (Fig. 5c and Supplementary Fig. 8). As expected, charging of E2 enzymes by UAE is also prevented by MLN7243. In contrast, BRD1732 only partially blocks UAE activity as indicated by ubiquitin charging of the E2 enzymes UBE2T, UBE2A/2B and UBE2L3, and BRD1732 has minimal effect on high-molecular-weight polyubiquitin conjugates at concentrations of up to 10 μM (Fig. 5c). These results are consistent with BRD1732 having a mechanism that is distinct from the UAE inhibitor MLN7243.

The terminal diglycine of ubiquitin is required for activation by ubiquitin ligases, conjugation to substrate proteins and removal by deubiquitinases^15,19,20^. In neurodegenerative diseases, molecular misreading results in a frameshift mutation in the ubiquitin B (*UBB*) gene resulting in production of an aberrant form of ubiquitin with G76 replaced by a 20-aa extension (Fig. 5d)^21^. Unanchored polyubiquitin chains terminating in UBB^+1^ have been reported to inhibit proteasomal degradation in cells and we wondered whether ubiquitin–BRD1732 conjugates could behave similarly^22^. While treatment with BRD1732 induced accumulation of multiple proteins known to undergo rapid proteasomal degradation, including Mcl1, Nrf1, p21 and p27, these proteins were not stabilized by overexpression of myc-tagged UBB^+1^ (Fig. 5e). Given the striking effects on these proteins, we wondered whether BRD1732 might be disrupting the UPS more broadly.

### BRD1732 disrupts ubiquitin-dependent proteasomal degradation

**Fig. 6:**
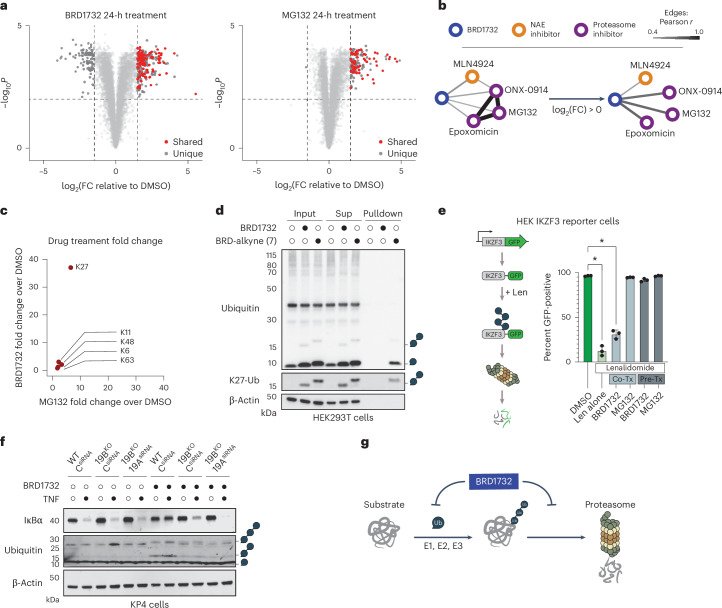
BRD1732 disrupts ubiquitin-dependent proteasomal degradation. **a**, Yamato synovial sarcoma cells were treated with 5 µM BRD1732 or 5 µM MG132 for 24 h and then analyzed by TMT quantitative proteomics. Plot shows only proteins with no significant change in corresponding gene expression by RNA-seq. Proteins with significant ‘shared’ (both BRD1732 and MG132) or ‘independent’ (BRD1732 or MG132 only) changes are highlighted with larger dots. Adjusted *P* values were calculated by a two-sided Student’s *t*-test followed by Benjamini–Hochberg correction (*n* = 3 biological replicates). **b**, Compound similarity by TMT quantitative proteomics in HCT116 cells. Network edge thickness corresponds to Pearson *r*. All nodes and network edges higher than 0.4 (Pearson *r*) are shown. **c**, Yamato cell TMT proteomics dataset from **a** analyzed for peptides corresponding to specific ubiquitin–ubiquitin linkages. **d**, HEK293T cells were treated with 5 µM BRD1732 or 5 µM BRD1732-alkyne (**7**) for 6 h and lysates were subjected to click reaction with biotin-azide and streptavidin pulldown followed by immunoblot. **e**, Lenalidomide induces proteasomal degradation of IKZF3–GFP. HEK293T cells stably expressing IKZF3–GFP were subjected to treatment with DMSO, 5 µM BRD1732 or 5 µM MG132 alone or in combination with lenalidomide and GFP levels were quantified by flow cytometry. Len, lenalidomide; co-Tx, co-treatment for 2 h; pre-Tx, pretreatment with BRD1732 or MG132 for 6 h before addition of lenalidomide for 2 h. Data are presented as the mean ± s.e.m. of three biological replicates. *P < 0.05 (two-tailed Student’s *t*-test for pairwise comparisons). **f**, KP4 WT or *RNF19B*-KO cells were transfected with *RNF19A* siRNA or control siRNA, subjected to 6-h pretreatment with 5 µM BRD1732 or DMSO followed by 20-min stimulation with TNF. IκBα degradation and polyubiquitin accumulation were analyzed by immunoblot. **g**, Proposed model wherein BRD1732 blocks ubiquitin-dependent proteasomal degradation. Immunoblots in **d** and **f** are representative of three independent experiments. Source data

To assess the effect of BRD1732 on protein homeostasis in cells, we performed quantitative proteomics comparing BRD1732 to a proteasome inhibitor, MG132, in Yamato synovial sarcoma cells (Fig. 6a and Supplementary Data 3 and 4). Focusing on proteins with no significant gene expression changes upon treatment by RNA-seq (log_2_FC (fold change) < 1, *P*_adj_ < 0.01), we identified 138 proteins that were stabilized by the proteasome inhibitor MG132 and only two proteins that were destabilized. In contrast, BRD1732 induced dramatic bidirectional changes in protein stability, with 225 proteins significantly increased and 94 proteins significantly decreased. Strikingly, 63 proteins were stabilized by both BRD1732 and MG132. Our RNA-seq data demonstrate that these changes are not transcriptionally driven but rather occur at the protein level.

To facilitate broader comparison to 877 drugs and tool compounds reported as part of DeepCoverMOA^23^, we performed similar quantitative proteomics in HCT116 colorectal cancer cells following treatment with BRD1732. Across this panel of molecules with a wide range of MOAs, BRD1732-induced protein-level changes correlated most strongly with the proteasome inhibitors MG132, epoxomicin and ONX-0941 (Pearson *r* = 0.4–0.43) and the NEDD8-activating enzyme inhibitor MLN4924 (*r* = 0.48) (Fig. 6b and Supplementary Data 5). Restricting this analysis to proteins that increase upon treatment with BRD1732, we found that the correlation with proteasome inhibitors strengthened (*r* = 0.58–0.6).

**Extended Data Fig. 1:**
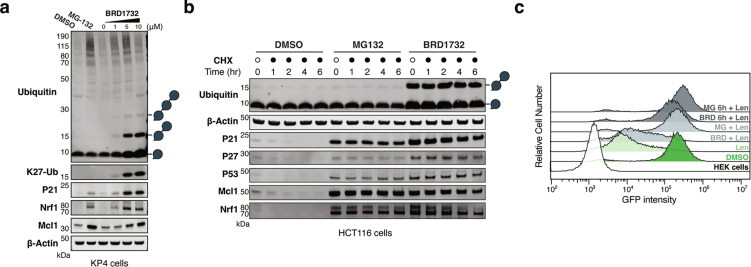
BRD1732 disrupts ubiquitin-dependent proteasomal degradation. **a**, Immunoblots of 24-h drug treatment of DMSO, 5 µM MG132, and indicated concentrations of BRD1732 in KP4 cells. **b**, Immunoblot of lysate from HCT116 cells treated for 24 h with DMSO, 5 µM MG132, or 5 µM BRD1732 followed by exposure to 20 ng/mL cycloheximide for indicated duration. **c**, (continuing of Fig. 6e) Lenalidomide induces proteasomal degradation of IKZF3-GFP. HEK293T cells stably expressing IKZF3-GFP were subjected to treatment with DMSO, 5 µM BRD1732, or 5 µM MG132 alone or in combination with lenalidomide, and GFP levels were quantified by flow cytometry. Len, Lenalidomide; co-Tx, co-treatment for 2 h; pre-Tx, pre-treatment with BRD1732 or MG132 for 6 h prior to addition of lenalidomide for 2 h. Immunoblots in **a** and **b** are representative of three biological experiments. Source data

Further analysis of our Yamato cell proteomics data revealed an increase in ubiquitin–ubiquitin linkages upon treatment with BRD1732, similar to changes seen with proteasome inhibition (Fig. 6c and Supplementary Fig. 9). Strikingly, however, BRD1732 induced dramatic accumulation of K27-bridged ubiquitin linkages. While BRD1732 and MG132 showed similarities at the proteome level, they differed in their effects on ubiquitin. Unlike BRD1732, MG132 did not induce accumulation of low-molecular-weight polyubiquitin chains but rather led to accumulation of high-molecular-weight polyubiquitinated substrate proteins (Extended Data Fig. 1a). In contrast, BRD1732 showed a bimodal effect on polyubiquitinated species, first increasing like MG132 and then decreasing with a more dramatic rise in low-molecular-weight ubiquitin conjugates. Immunoblot analyses demonstrated that BRD1732-induced diubiquitin species contained the uncommon K27 ubiquitin linkage. Accumulation of this species correlated closely with accumulation of several proteins that were also stabilized by MG132, including p21, p27, p53, Nrf1 and Mcl1 (Extended Data Fig. 1a,b). Using cycloheximide chase experiments, we confirmed that the accumulation of these proteins resulted from prolongation of their half-lives (Extended Data Fig. 1b).

We wondered whether the BRD1732-induced K27-linked diubiquitin species might form as a conjugate on BRD1732. Using an alkyne-modified BRD1732 analog, BRD-alkyne (**7**), and subjecting lysate from treated cells to a click reaction with biotin-azide before immunoblot analysis, we discovered that K27-linked diubiquitin species were indeed conjugated to BRD1732 (Fig. 6d). After biotin-azide click, diubiquitin species in BRD-alkyne treated cells shifted higher corresponding to the added mass of biotin-azide. Following pulldown with streptavidin beads, immunoblot analyses demonstrated that both monoubiquitin and K27-linked diubiquitin were covalently conjugated to BRD1732.

To further characterize the effect of BRD1732 on ubiquitin-dependent proteasomal degradation, we evaluated its effect on lenalidomide-induced degradation of IKZF3 using a fluorescent reporter system consisting of IKZF3-GFP expressed under the SFFV promoter and mCherry expressed from an internal ribosome entry site, developed by Dr Benjamin Ebert. Lenalidomide induces ubiquitination and degradation of IKZF3 by the E3 ubiquitin ligase cereblon^5^. Using our reporter system, we see loss of GFP signal upon treatment with lenalidomide corresponding to degradation of IKZF3, and co-treatment with MG132 blocks this effect. While simultaneous co-treatment with BRD1732 only minimally affected IKZF3 degradation by lenalidomide, pretreatment with BRD1732 for 6 h robustly blocked degradation (Fig. 6e and Extended Data Fig. 1c). To assess the effect of BRD1732 on proteasomal degradation in an endogenous system, we evaluated tumor necrosis factor (TNF)-induced degradation of inhibitor of kappa B alpha (IκBα) (Fig. 6f and Supplementary Fig. 10). Following 6-h pretreatment, BRD1732 efficiently blocked degradation of IκBα following stimulation with TNF. Importantly, we saw evidence of intact IκBα phosphorylation and polyubiquitination, the two proximal signals for proteasomal degradation of IκBα, suggesting that BRD1732 interfered with degradation at the level of the proteasome itself (Supplementary Fig. 10). As expected, cells lacking expression of one or both RNF19 proteins showed reduced production of the ubiquitin–BRD1732 conjugate and less inhibition of IκBα degradation.

**Extended Data Fig. 2:**
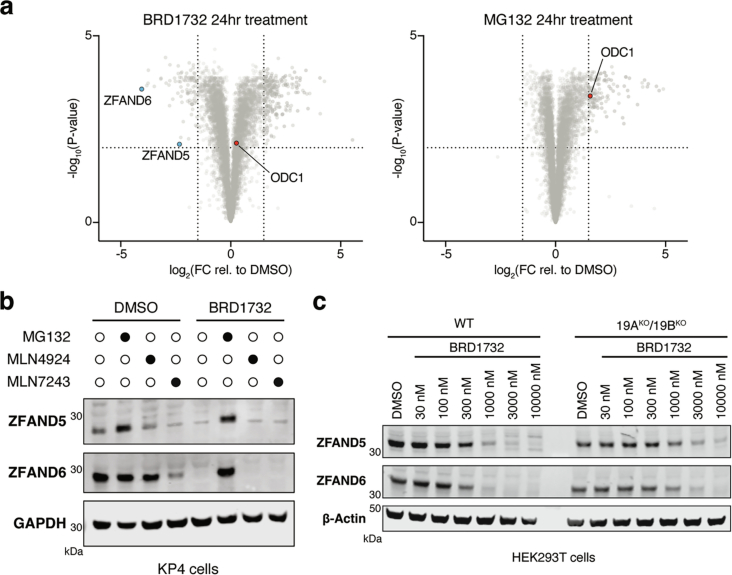
BRD1732 promotes degradation of ZFAND5 and ZFAND6 in an RNF19-dependent manner. **a**, Yamato synovial sarcoma cells were treated with 5 µM BRD1732 or 5 µM MG132 for 24 h, then analyzed by TMT quantitative proteomics. Plot shows only proteins with no significant change in corresponding gene expression by RNA-Seq (same data presented in Fig. 6a). Adjusted *p*-values were calculated by two-sided Student’s t-test followed by Benjamini-Hochberg correction. n = 3 biological experiments. **b**, ZFAND5 and ZFAND6 levels were analyzed by immunoblot of lysates from KP4 cells treated with 10 µM MG132, 500 nM MLN4924, or 50 nM MLN7243 in combination with either DMSO or 10 µM BRD1732 for 6 h. **c**, Immunoblot analysis of ZFAND5 and ZFAND6 protein levels in wild-type (WT) and RNF19A/RNF19B double-knockout (19AKO/19BKO) HEK293T cells treated with DMSO or indicated concentration of BRD1732 for 6 h. Immunoblots in **a** and **b** are representative of three biological experiments. Source data

In considering the bidirectional effects of BRD1732 in our proteomic analysis, we wondered whether BRD1732 might block ubiquitin-dependent proteasomal degradation while leaving ubiquitin-independent proteasomal degradation intact (Extended Data Fig. 2a). Indeed, we saw no stabilization of ornithine decarboxylase, a classic substrate for ubiquitin-independent proteasomal degradation because of its strong PEST sequence^24^. In addition, BRD1732 induced loss of two homologous zinc finger proteins, ZFAND5 and ZFAND6, and this effect was blocked by co-treatment with MG132 but not MLN7243, consistent with ubiquitin-independent proteasomal degradation (Extended Data Fig. 2b). Like other cellular effects of BRD1732, degradation of ZFAND5 and ZFAND6 is dependent on RNF19 proteins (Extended Data Fig. 2c). Further studies to understand the mechanism of degradation of ZFAND5 and ZFAND6 by BRD1732 are underway. Together our results are consistent with a model in which BRD1732 is ubiquitinated by UBE2L3 and RNF19 proteins and accumulation of the resulting ubiquitin–BRD1732 conjugate leads to inhibition of ubiquitin-dependent proteasomal degradation (Fig. 6g).

## Discussion

Small-molecule MOA studies have repeatedly helped to lay the groundwork for new fields of drug discovery. However, even when a target can be identified, achieving a deep understanding of a small molecule’s mechanism remains a considerable challenge. Powerful modern tools such as genome-wide CRISPR screening can be illuminating but these are inadequate for unveiling the details of a chemical mechanism, which is still only achievable through highly tailored biochemical studies.

Here, we used a combination of CRISPR screening, proteomics and classical biochemistry to uncover a previously unknown small-molecule MOA wherein BRD1732 receives a protein modification, ubiquitin, that is thought to serve almost exclusively as a posttranslational modification on other proteins. Nearly all known covalent small-molecule probes and drugs contain reactive electrophiles to allow covalent modification of nucleophilic amino acids, such as cysteine, serine or tyrosine, on their protein targets. There, the reactive moiety resides within the small molecule. For BRD1732, the directionality of the reaction is reversed. BRD1732 contains a relatively unreactive nucleophile, the azetidine nitrogen, which forms a covalent bond with the C terminus of ubiquitin. In this case, the reactive electrophile is the thioester bond between the ubiquitin C terminus and the catalytic cysteine of the ubiquitin ligase. Even more surprisingly, the ubiquitinated azetidine nitrogen is a secondary amine, unlike the primary amine of lysine side chains.

Nucleotide-mimetic inhibitors of E1 enzymes MLN7243 and MLN4924 also form covalent conjugates with the C terminus of ubiquitin and NEDD8, respectively. However, while these molecules function through product inhibition blocking the continued production of conjugate, the ubiquitin–BRD1732 conjugate rapidly accumulates in cells, suggesting efficient turnover. Despite this dramatic accumulation, we do not see evidence that cellular free ubiquitin stores are depleted upon treatment with BRD1732. Given the wide-ranging functions and interactors of ubiquitin in the cell, it is unsurprising that BRD1732 shows pleiotropic effects culminating in disruption of the UPS and proteasomal degradation.

In addition, we find that ubiquitin–BRD1732 conjugates consist of a combination of monoubiquitinated and diubiquitinated species and these diubiquitin species are predominantly assembled through the atypical K27 linkage. Unlike the canonical K48-linked ubiquitin chains, which typically serve as direct signals for proteasomal degradation, K27-linked ubiquitin chains are less common and are reported to have a role in p97 substrate processing of nuclear proteins and nonproteolytic signaling pathways^25–27^. Diubiquitin linked through K27 is also stable to most deubiquitinases, a feature that may account for its accumulation in BRD1732-treated cells^10,28^. Together, while BRD1732 shows similarity with active site proteasome inhibitors at the transcriptional and proteomic level, BRD1732 functions through a distinct mechanism and is unique in causing widespread bidirectional effects on protein homeostasis.

Ubiquitination of BRD1732 is both highly stereospecific and highly dependent on expression of RNF19 proteins. While we have been unable to reconstitute ubiquitination of BRD1732 by RNF19 with recombinant proteins despite multiple attempts, on the basis of our CRISPR–Cas9 KO and subsequent re-expression experiments, it is highly likely that RNF19 proteins are directly responsible for ubiquitination of BRD1732. Ubiquitination of nonproteinaceous substrates has only rarely been reported, notably with ubiquitination of lipopolysaccharide by RNF213 upon entry of *Salmonella* into the cytosol^7^. Our demonstration of ubiquitination of a small molecule in cells raises the possibility of noncovalently directing ubiquitin to any protein target using bifunctional compounds related to BRD1732 within the cell. Indeed, our work more generally raises the possibility of posttranslational modification in *trans*, the delivery of a posttranslational mark through a small-molecule intermediary.

## Methods

### Antibodies and expression constructs

The details and sources of the antibodies and plasmid constructs that were used in this study are included in the Supplementary Tables 1 and 2, respectively. In addition, human RNF19A variant constructs were synthesized and cloned into the pLVX6 backbone by Twist Biosciences. All constructs were verified by whole-plasmid sequencing (Plasmidsaurus).

### Cell culture and reagents

PC9, MM.1S and HEK293T cells were purchased from the American Type Culture Collection. HCT116 cells were obtained from the Broad Institute Genetic Perturbation Platform. KP4 cells were purchased from the Japanese Collection of Research Bioresources Cell Bank. HEK293T IKZF3 reporter cells were a gift from the laboratory of B. Ebert (Dana-Farber Cancer Institute). Expi293F cells were purchased from Thermo Scientific. HAP1 cells were purchased from Horizon Discovery. All cell lines were confirmed to be *Mycoplasma* free. Transient transfections were carried using TransIT-LT1 reagent (Mirus Bio). HEK293T, HCT116 and KP4 cells were maintained in DMEM (Gibco) containing 10% (v/v) FBS (Gibco). PC9 and MM.1S cells were maintained in RPMI medium (Gibco) containing 10% FBS (Gibco). Expi293F cells were maintained in Expi293 expression medium (Thermo Scientific). HAP1 cells were maintained in IMDM (Gibco) containing 10% FBS (Gibco).

### Cell viability assays

Cells were seeded in 96-well plates at 1,000 cells per well in 90 μl of corresponding medium with 10% FBS. Following attachment for 24 h, cells were treated by addition of 10 μl of compound dilution or vehicle (0.1% DMSO final). After 72 h, cell viability was quantified using the CellTiterGlo luminescent cell viability assay (Promega) measuring luminescence on a SpectraMax M3 plate reader or BioTek Synergy 2 plate reader installed with Biotech Gen5 version 3.03 software. Data were processed using GraphPad Prism.

### Chemical compounds

The compounds BRD1732 (**1**), BRD-E (**2**), BRD-D (**3**), BRD-OMe (**4**), BRD-NMe (**5**), BRD-NOMe (**6**) and BRD-alkyne (**7**) were synthesized in house for use in this study. Detailed synthetic procedures and characterization data are provided in the Supplementary Note. The details of purchased chemical compounds are also included in the Supplementary Note.

### Multiplexed quantitative proteomic analysis in Yamato cells

Yamato synovial sarcoma cells were plated in 10-cm dishes at a density of 2 × 10^6^ cells per ml and allowed to grow for 48 h or until 75% confluency was reached. These cells were treated with BRD1732 at 1 or 5 μM, MG132 at 5 μM or DMSO for 24 h at a final DMSO concentration of 1% v/v. Cells were harvested, washed with ice-cold PBS and lysed in 200 μl of radioimmunoprecipitation assay (RIPA) buffer (50 mM Tris-HCl pH 7.5, 150 mM NaCl, 1% Triton X-100, 0.1% SDS, 0.5% sodium deoxycholate, 1 mM DTT and cOmplete protease inhibitors (Roche)). Protein concentrations were measured using the DC protein assay (Bio-Rad). Data were acquired using Xcalibur version 4.2 and analyzed as previously described^29^. Briefly, a 0.003-Da window centered on each reporter ion’s theoretical *m*/*z* was used to record the closest intensity signal. Tandem mass tag (TMT) signals were corrected for isotope impurities per manufacturer’s instructions. Peptides were quantifiable only if total signal-to-noise across channels exceeded 200 and isolation specificity was >0.75. Peptide intensities were summed and normalized across channels. Protein quantitation was based on summed peptide signal-to-noise, scaled to 100. The adjusted *P* value was calculated by Student’s *t*-test followed by Benjamini–Hochberg procedure. Proteomic datasets were processed to remove proteins with expression changes by RNA-seq (|log_2_FC| > 1 and −log_10_(*P*_adj_) > 2, where *P*_adj_ is the Benjamini–Hochberg-corrected *P* value). Resulting datasets for 5 µM BRD1732 and 5 µM MG132 were compared to identify proteins with log_2_FC > 1.5 and −log_10_(*P*_adj_) > 2. Plots were generated with GraphPad Prism.

### RNA-seq analysis in Yamato cells

Yamato cells were plated in 15-cm dishes at a density of 2× 10^6^ cells per ml and allowed to grow for 48 h or until 75% confluency was reached. These cells were then treated with BRD1732 at 5 µM, MG132 at 5 µM or DMSO in triplicate at a final DMSO concentration of 1% v/v and incubated for 24 h. Following perturbation, the medium was removed and 1 ml of TRIzol reagent (Thermo Fisher) was added to each well; RNA was extracted according to the manufacturer’s protocol. Quantification of the samples was performed using the Qubit RNA HS assay kit (ThermoFisher) in a Qubit fluorometer (Thermo Fisher). All library preparation and sequencing (75-bp single-end reads on Illumina NextSeq 500) was performed in the Molecular Biology Core Facilities at the Dana-Farber Cancer Institute. RNA-seq reads were mapped to the human reference genome (hg19) using STAR (version 2.3.1)^30^ with default parameters. All error bars represent the mean ± s.e.m. Significance was assessed using the R package DESeq2 (ref. ^31^) using raw read counts generated with Rsubread featureCounts against the hg19 refFlat annotation.

### Genome-wide CRISPR resistance screening

KP4 cells expressing Cas9 were infected with the AVANA genome-wide sgRNA library^32^ and plated in T175 flasks at a density of 2 × 10^6^ cells per ml and allowed to adhere for 48 h. Cells were then treated with BRD1732 at 10 μM or DMSO for 3 days and allowed to recover from treatment for additional 7 days. Following recovery, cells were harvested and genomic DNA was isolated using Maxi kits according to the manufacturer’s protocol (Qiagen). Next-generation sequencing was performed by the Genomics Platform at the Broad Institute.

MM.1S multiple myeloma cells engineered to stably express the nuclease SpCas9 (provided by Q. L. Sievers in the B. Ebert lab) were also transduced with a pool of lentiviral particles for ~70,000 sgRNAs (Brunello library), targeting exons of ~20,000 genes (plus nontargeting control sgRNAs), under conditions of transduction that allow for an average of no more than one sgRNA to be incorporated in a given cell^33^. The MM.1S-Cas9^+^ Brunello-transduced cells were then cultured in the presence or absence of increasing doses of BRD1732 (0.5–4 µM). The treated versus control cultures of MM.1S cells were passaged serially over 10 weeks and were periodically checked for BRD1732 sensitivity using CTG. When the dose–response curve identified the isolation of BRD1732-resistant MM cells, these were isolated from the cultures and processed for sequencing. Data were analyzed with the MAGeCK package using default parameters.

### Multiplexed quantitative proteomics time course and DeepCoverMOA

HCT116 cells were plated in six-well plates at a density of 1 × 10^6^ cell per well. After attachment for 24 h, cells were treated with 5 µM BRD1732 for 1, 2, 4 or 24 h or 0.5% (v/v) DMSO for 24 h. Cells were then washed with PBS three times and lysed with 8 M urea, 0.1% SDS and 200 mM EPPS buffer pH 8.5 containing cOmplete protease inhibitor cocktail tablets (Roche) and PhosSTOP (Roche). Samples were processed as previously described^23^ but using 16-plex TMTpro reagents. Briefly, 30 μg of protein was reduced with TCEP (5 mM, 15 min), alkylated with iodoacetamide (20 mM, 30 min) and then extracted using single-pot, solid-phase-enhanced sample preparation technology. Proteins were digested overnight at 37 °C using 0.3 μg of LysC (Wako) and 0.3 μg of trypsin (Thermo) before TMT labeling. TMT-labeled peptides were pooled then desalted using a 50-mg SepPak (Waters) before offline basic pH reverse-phase HPLC fractionation, generating 24 fractions. A total of 12 nonadjacent fractions were desalted using in-housed packed stagetips and then analyzed using a real-time search MS3 method. The adjusted *P* value was calculated by Student’s t-test followed by Benjamini–Hochberg procedure. Plots were generated using GraphPad Prism. Data corresponding to the log_2_FC from the 24-h time point were subjected to comparative analysis by DeepCoverMOA (http://wren.hms.harvard.edu/DeepCoverMOA/)^23^. When limiting analysis to proteins that increase upon treatment with BRD1732, data for proteins with log_2_FC < 0 were removed and the remaining data were subjected to analysis by DeepCoverMOA. Networks were generated using Cytoscape.

### Immunoblotting

Cells were plated into a 12-well plate (Costar, 3513) with 100,000 cells per well (final volume: 1 ml) and incubated overnight. Cells at 40–60% confluency were treated with compounds or 0.5% (v/v) DMSO. After drug treatment for indicated times, cells were lysed in CelLytic M (Sigma-Aldrich) reagent supplemented with 1× EDTA-free protease inhibitor cocktail tablet (Sigma-Aldrich) for 40 min on a rotating wheel at 4 °C. Lysates were separated on 4–12% Bis–Tris SDS–PAGE (Invitrogen) in MES buffer (Invitrogen) at 150 V for 45 min. Proteins were transferred to a PVDF membrane using dry transfer method with iBlot 2 (Invitrogen) following the manufacturer’s instructions. The membrane was blocked with 5% BSA (RPI) in TBST buffer at room temperature for 1 h followed by incubation with primary antibody at 4 °C overnight, followed by secondary antibody incubation for 1 h at room temperature. Immunoblots were then imaged by using a Li-Cor Odyssey CLx imaging system with Image Studio 5.0 software. All antibodies are listed at the end of the section.

### PRISM profiling

PRISM profiling was performed as previously described^34^. The PRISM cell set used consisted of 483 solid tumor cancer cell lines. These cell lines largely overlap with and reflect the diversity of the Cancer Cell Line Encyclopedia cell lines (https://portals.broadinstitute.org/ccle). Cell lines were grown in RPMI with 10% FBS without phenol red. Parental cell lines were stably infected with a unique 24-nt DNA barcode by lentiviral transduction and blasticidin selection. After selection, barcoded cell lines were expanded and quality-checked (*Mycoplasma* contamination test, a sinle-nucleotide polymorphism test for confirming cell line identity and barcode confirmation). Passing barcoded lines were then pooled (20–25 cell lines per pool) on the basis of doubling time and frozen in assay-ready vials. A Labcyte Echo was used to acoustically transfer test compounds were to 384-well plates at eight doses with threefold dilutions in triplicate. These assay-ready plates were then seeded with the thawed cell line pools. Adherent cell pools were plated at 1,250 cells per well. Treated cells were incubated for 5 days then lysed with QIagne TCL buffer and frozen. Lysate plates were collapsed together before barcode amplification and detection. Each cell line’s unique barcode was located at the end of the blasticidin resistance gene and was expressed as mRNA. These mRNAs were then captured using magnetic particles that recognize poly(A) sequences. mRNA was then reverse-transcribed into complementary DNA and then the sequence containing the unique PRISM barcode was amplified using PCR. Finally, Luminex beads that recognize the specific barcode sequences in the cell set were hybridized to the PCR products and then detected using a Luminex scanner that reports signal as a median fluorescence intensity.

### Endogenous ubiquitin purification

Ubiquitin–BRD1732 conjugate was prepared as follows. Expi293T cells were grown in 125-ml shaker flasks at 37 °C and 8% CO_2_. Cells were transiently transfected with pLVX6 RNF19A using the ExpiFectamine 293 transfection kit following the manufacturer’s instructions. After incubation overnight, the medium was exchanged to Expi293 expression medium containing 10 µM BRD1732 and cells were incubated for 6 h. The cell stock was then resuspended in lysis buffer (50 mM ammonium chloride, pH 6.0) supplemented with EDTA-free protease inhibitor cocktail tablet (Sigma-Aldrich) and lysed by sonication. The sample were then centrifuged at 20,199*g* for 30 min at 4 °C using JA-25.50 rotor. The supernatant was collected and filtered using a 0.45-µm filter (Millex) before loading on a 1-ml HiTrap Q HP column (Cytiva) using fast protein LC (FPLC) on ÄKTA pure (Cytiva). Ubiquitin–BRD1732 conjugates were eluted with a salt gradient from 0 to 1 M NaCl in lysis buffer. The pooled protein sample was further purified by SEC using a Superdex 75 Increase 10/300 GL (Cytiva) column in PBS buffer. In comparison, WT ubiquitin was purified from Expi293T cells using the abovementioned method except for the steps of pLVX6 plasmid transfection and BRD1732 treatment.

### Protein expression and purification

HA–tUI protein was purified as previously described^17^. Briefly, HA–tUI protein cloned on pET28 (Addgene, 122662) was transformed into BL21 (DE3) Star II *Escherichia coli* (QB3-Berkeley) for protein expression. The expression was induced by adding 0.4 mM IPTG to cells grown at 37 °C until the optical density at 600 nm reached 0.6 to 0.8. After a 30-min ice bath, the cells were left to grow at 20 °C overnight. The cells were then centrifuged at 6,000*g* for 25 min at 4 °C. The collected pellets were resuspended in buffer A (20 mM sodium phosphate, 500 mM NaCl, 20 mM imidazole and 1 mM TCEP, pH 7.4) and sonicated for cell lysis. The lysate was further centrifuged at 20,199*g* for 30 min at 4 °C using a JA-25.50 rotor. The supernatant was filtered against a 0.45-µm filter (Millex) before loading on a 5-ml HisTrap HP column (Cytiva) for FPLC. The protein was eluted by the imidazole gradient from 20 mM to 500 mM in buffer A. After an overnight buffer dialysis into buffer B (PBS and 1 mM TCEP, pH 7.4) where the protein solution was held in the 3.5-kDa cutoff tubing (Spectrum Labs), protein was then purified by SEC using a HiLoad 16/600 Superdex 200 pg (Cytiva) column in buffer B.

### Intact protein LC–MS

LC–MS was performed on a Xevo G2-XS quadrupole time-of-flight MS instrument coupled with an Acquity ultrahigh-performance LC (UPLC) system equipped with an Acquity UPLC protein BEH C4 column (300 Å, 1.7 µm, 2.1 mm × 50 mm). Buffer A (water supplemented with 0.1% formic acid) and buffer B (acetonitrile supplemented with 0.1% formic acid) were used as the mobile phase. The flow rate was 0.5 ml min^−1^. Spectra were deconvoluted and analyzed using Masslynx version 4.2 software.

### Trypsin digestion and MS/MS analysis of ubiquitin–BRD1732

FPLC–SEC-purified ubiquitin–BRD1732 conjugate in PBS buffer (pH 7.4) was trypsin-digested and analyzed by tandem MS. Briefly, purified ubiquitin–BRD1732 conjugate (~1 µM, 100 µl) in PBS was buffer exchanged into digestion buffer A (20 mM Tris pH 8.0 and 2 mM CaCl_2_) using 0.5-ml 3-kDa-cutoff centrifugal filters (Amicon). Trypsin digestion was performed by adding 0.5 mg trypsin into the sample and incubating overnight at 37 °C. The digestion reaction was stopped by adding 5 µl of 10% formic acid to reach a final concentration of 0.5 % (v/v) formic acid. Digested peptides were characterized by a Waters Xevo G2-XS system equipped with an Acquity UPLC BEH C4 1.7-µm column using a linear gradient of 5–95% acetonitrile in water + 0.05% formic acid. Gating on the ion at *m*/*z* = 519, we applied a collision energy of 20 eV. A similar protocol for trypsin digestion and intact protein LC–MS was used for unmodified ubiquitin.

### Quantification of free ubiquitin

HEK293T cells were plated 200,000 cells per well in a 12-well plate and incubated at 37 °C with 5% CO_2_ overnight. Drug treatments were performed by treating the cells with different concentration of BRD1732 for 6 h. Cells were detached by trypsinization using TrypLE express enzyme (Thermo Fisher) and then washed by ice-cold PBS buffer (all following PBS washes used ice-cold PBS buffer). Next, cells were fixed by incubating with 4% formaldyhyde (Sigma-Aldrich) in PBS buffer for 15 min at room temperature, followed by a PBS wash. Cells were then permeabilized by slowly dropping ice-cold 100% methanol (Fisher Scientific) with vertexing to the final concentration of 90% methanol, followed by a 30-min incubation on ice and a PBS wash. Next, cell samples were incubated with blocking buffer (5% BSA (RPI) in TBST buffer) for 1 h to block the nonspecific binding. After that, samples were divided into two groups. The experimental group was stained with 100 nM purified HA–tUI protein in blocking buffer for 30 min at room temperature to detect the free ubiquitin concentration. In the control group, purified 100 nM HA–tUI protein was mixed with 10 µM ubiquitin protein (Enzo Life Sciences) for 5 min at room temperature in blocking buffer before adding to the sample. Three PBS washes were performed with at least 5-min intervals between each. Cells were then incubated overnight at 4 °C with anti-HA monoclonal antibody conjugated with Alexa Fluor 750 (Cell signaling) in a black box with 1:500 dilution. The next day, samples were washed three times with PBS buffer before loaded with 200 µl of PBS supplemented with 1 % BSA. Samples were then transferred to a 96-well plate and analyzed on a CytoFLEX S flow cytometer (Beckman) using CytExpert software. The results were then processed using FlowJo.

### IKZF3 reporter assay

HEK293T IKZF3 cells were plated at 3 × 10^5^ cells per well in 12-well plates. After attachment for 24 h, cells were treated with 5 µM BRD1732, 5 µM MG132 or 0.1% (v/v) DMSO for 6 h. The medium was then exchanged to include 200 nM lenalidomide with indicated compounds for an additional 2 h. Cells were detached using TrypLE Express (Thermo), resuspended in PBS with 10% (v/v) FBS, centrifuged for 2 min at 300*g*, resuspended in PBS with 1% (v/v) FBS and analyzed by flow cytometry on a CytoFLEX S flow cytometer. Data were processed using FlowJo (Supplementary Fig. 11). Data represent three biological replicates.

### BRD1732-alkyne pulldown assay

HEK293 cells were seeded in 10-cm^2^ dishes at 37 °C with 5% CO_2_ overnight before treatment with 5 μM BRD1732 or its alkyne-tagged derivative, BRD-alkyne, for 6 h. Following treatment, cells were washed with PBS, detached using TrypLE express enzyme (12604013, Thermo Fisher) and collected by centrifugation at 300*g* for 3 min. The cell pellet was washed once with PBS (MT21031CV, Corning), resuspended in the lysis buffer of PBS containing protease and phosphatase inhibitors (78446, Thermo Fisher) and lysed by sonication. Lysates were cleared by centrifugation at 20,627*g* for 10 min at 4 °C. Click chemistry labeling was performed using biotin-picolyl azide in a copper(I)-catalyzed azide–alkyne cycloaddition reaction^35,36^. To 47 μl of lysate, the following reagents were sequentially added in a final volume of 50 μl: 2.5 mM sodium ascorbate (11140, Sigma-Aldrich), 0.1 mM biotin-picolyl azide (900912, Sigma-Aldrich), 0.5 mM THPTA ligand (762342, Sigma-Aldrich) and 0.25 mM copper sulfate (209198, Sigma-Aldrich). The reaction mixture was vortexed, briefly spun down and incubated in the dark for 30 min at room temperature. Excess click chemistry reagents were removed using a Zeba spin desalting column (PI89882, Thermo Scientific) following the manufacturer’s protocol. A portion of the sample was reserved for input immunoblots and the rest of the sample was diluted up to 300 μl with lysis buffer. Biotinylated proteins were then captured using streptavidin magnetic beads (88816, Thermo Fisher) by incubating the diluted and desalted lysate with streptavidin beads for at least 1 h at room temperature with rotation. After reservation of a portion of the supernatant for immunoblots, beads were washed extensively with TBS (BP2471, Thermo Fisher) containing 0.1% Tween-20 detergent (P1379, Sigma-Aldrich) to remove nonspecifically bound proteins. Bound proteins were eluted using 2× SDS sample buffer (J61337-AD, Thermo Scientific) and analyzed by immunoblotting.

### Generation of KO and DKO stable cells

KP4 or HEK293T cells were reverse-transfected with preformed Cas9 ribonucleoprotein (RNP) with EnGen Cas9 NLS (New England Biolabs), CRISPR RNA (crRNA) targeting RNF19B (target sequence: TCGTACTTGTGCATAAGCGG) and *trans*-activating crRNA (Integrated DNA Technologies (IDT)) using Lipofectamine CRISPRMAX (Thermo). Cells were allowed to attach for 24 h then were selected with 5 µM BRD1732 for 24 h to obtain RNF19B-KO cells. Following two passages, RNF19B-KO cells were similarly reverse-transfected with Cas9 RNP with crRNA targeting RNF19A (target sequence: AAGAATTTATGCTTAGACGG). Following attachment for 24 h, cells were selected with 20 µM BRD1732 for 24 h to obtain RNF19A/B-DKO cells.

CRISPR–Cas9 KO in HAP1 cell lines with single clonal selection was performed as follows. gRNA of the gene of interest was selected from a predesigned online library from IDT. We used the following sgRNA sequences:

RNF19A: AAGAATTTATGCTTAGACGG

RNF19B: CCGCTTATGCACAAGTACGA

UBE2L3: TATGATAAGGGAGCCTTCAG

The Cas9 RNP complex, containing synthesized gRNA from IDT and S.p. HiFi Cas9 (1081061, IDT), was prepared using the Neon transfection kit (MPK10025K, Thermo Fisher) following the company’s protocol. The RNP complex was then delivered into cells using the Neon transfection system (MPK5000, Thermo Fisher) according to the company’s protocol with some modifications. Briefly, for each transfection, 1 × 10^6^ cells were electroporated with RNP complex (7.5 µg of gRNA + 30 µg of Cas9) in 120 µl of reaction buffer with the following settings on Neon: 1,575 V, 10 ms and three pulses. After electroporation, cells were seeded in a six-well culture plate in IMDM (Gibco) and cultured for 2 days. After transfection, single-cell clones were isolated through limiting dilution into 96-well plates (3997, Corning). Expanded single-cell clones were screened for successful KO by PCR amplification and subsequent sequencing analysis (Genewiz) of targeted genomic regions to confirm the absence or mutation of the targeted genes.

### Small interfering RNA (siRNA)-mediated knockdown

KP4 WT or RNF19B-KO cells were reverse-transfected with Silencer siRNA targeting RNF19A (134494, Thermo) or Silencer Select siRNA control (AM4611, Thermo), plating cells at 8 × 10^5^ per well. Following attachment for 24 h, cells were treated with 5 µM BRD1732 or DMSO 0.5% (v/v) DMSO for 6 h followed by 20 ng ml^−1^ TNF or water in medium for 10 min. Plates were placed on ice and cells were harvested for immunoblot analysis by the addition of cold RIPA buffer.

### Reporting summary

Further information on research design is available in the Nature Portfolio Reporting Summary linked to this article.

## Online content

Any methods, additional references, Nature Portfolio reporting summaries, source data, extended data, supplementary information, acknowledgements, peer review information; details of author contributions and competing interests; and statements of data and code availability are available at 10.1038/s41589-025-02011-1.

## Supplementary information

Supplementary Information Supplementary Figs. 1–11, Tables 1 and 2, Note (chemical synthesis, NMR spectra and purchased compounds) and source data.

Reporting Summary

Supplementary Data 1 Summary of genome-wide CRISPR resistance screening summary.

Supplementary Data 2 Summary of PRISM.

Supplementary Data 3 Summary of TMT proteomics in Yamato cells.

Supplementary Data 4 Summary of TMT proteomics and RNA-seq in Yamato cells.

Supplementary Data 5 Summary of TMT proteomics in HCT116 cells and DeepcoverMOA Pearson correlation coefficient.

## Source data

Source Data Fig. 2 Unprocessed western blots.

Source Data Fig. 3 Unprocessed western blots.

Source Data Fig. 4 Unprocessed western blots.

Source Data Fig. 5 Unprocessed western blots.

Source Data Fig. 6 Unprocessed western blots.

Source Data Extended Data Fig. 1 Unprocessed western blots.

Source Data Extended Data Fig. 2 Unprocessed western blots.

## Data Availability

There are no restrictions on the availability of the data. Proteome quantification data are available from the PRIDE repository under accession numbers PXD053243 and PXD065168. Source data are provided with this paper.
