# Supplementary material for: Highly specific intracellular ubiquitination of a small molecule
Source: Nat Chem Biol. 2025 Aug 21;22(4):663–71. doi: 10.1038/s41589-025-02011-1 (PMC13038411; doi:10.1038/s41589-025-02011-1)
Supplement: Supplementary file 1 — Supplementary Figs. 1–11, Tables 1 and 2, Note (chemical synthesis, NMR spectra and purchased compounds) and source data. [file 41589_2025_2011_MOESM1_ESM.pdf]

nature chemical biology

Supplementary information https://doi.org/10.1038/s41589-025-02011-1

Highly specific intracellular ubiquitination of a small molecule

In the format provided by the authors and unedited

Table of Contents

Supplementary Figures	p.2
Supplementary Tables	p.13
Supplementary Note: Chemical synthesis	p.15
Supplementary Note: NMR Spectra	p.23
Supplementary Note: Purchased compounds	p.31
Supplementary Note: Source Data	p. 32

Supplementary Figures

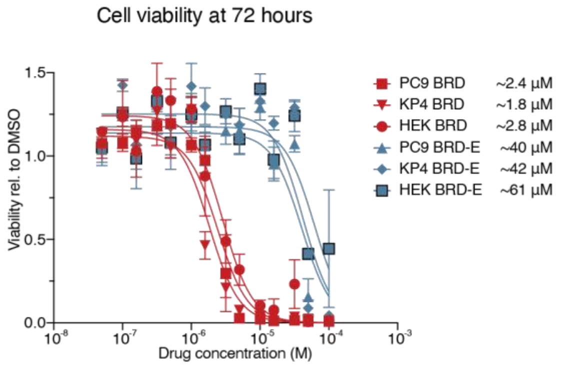

Supplementary Figure 1. BRD1732 viability effects are stereospecific across a range of cell line lineages. Cell viability after 72-hour drug treatment of BRD1732 (BRD, **1**) and BRD-E (**2**) for indicated cell lines. Fit was generated by 4-variable non-linear regression (n = 3 technical replicates, n = 2 biological replicates).

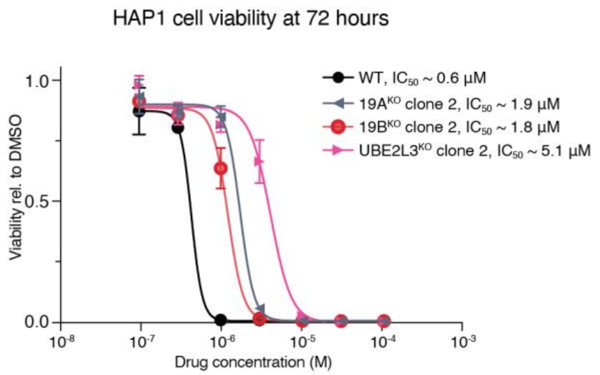

Supplementary Figure 2. Cell viability of CRISPR/Cas9-edited HAP1 cells following treatment with BRD1732. Cell viability after 72-hour drug treatment of BRD1732 in CRISPR/Cas9 knockout cell lines with indicated genotypes. Mean ± s.d., *n* = 6 biological replicates representing at least two independent experiments, except for wild-type (WT) and double knock-out (DKO) cells where *n* = 9 biological replicates were analyzed.

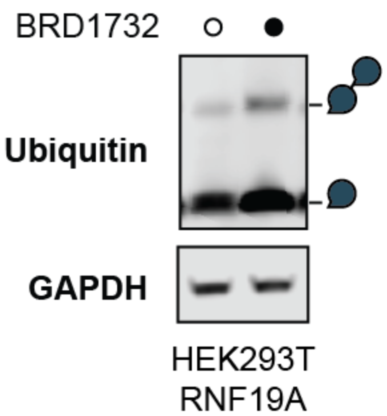

Supplementary Figure 3. Accumulation of ubiquitin species is prominent with RNF19A overexpression. HEK293T cells were transfected with GFP-RNF19A and treated with DMSO or 5μM BRD1732 for 6 hours followed by immunoblot analysis. Immunoblots are representative of three biological experiments.

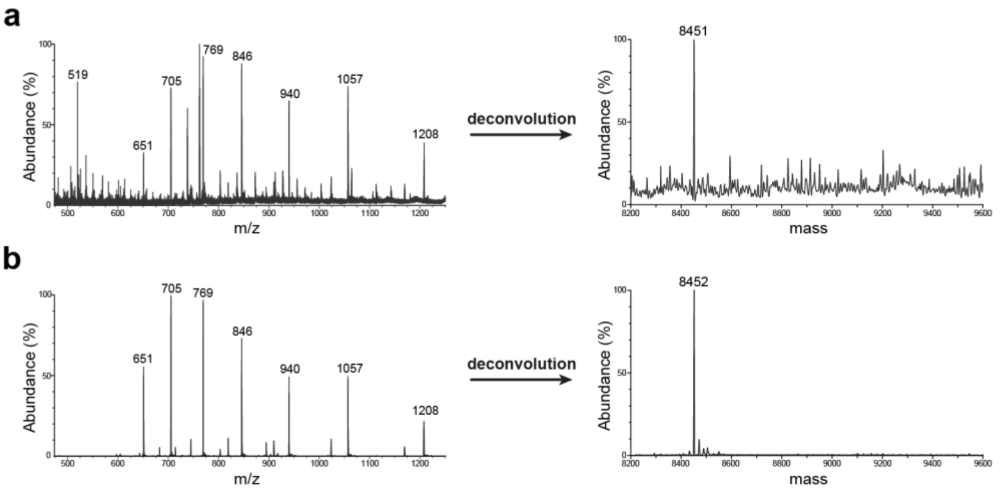

Supplementary Figure 4. LC-MS of ubiquitin and ubiquitin conjugate following trypsin digestion. a, Intact protein LC-MS of ubiquitin purified from BRD1732-treated Expi293F cells treated with trypsin under non-denaturing conditions (data also shown in Fig. 2**d**). b, Intact protein LC-MS of recombinant ubiquitin treated with trypsin under non-denaturing conditions. Images are representative of three biological experiments.

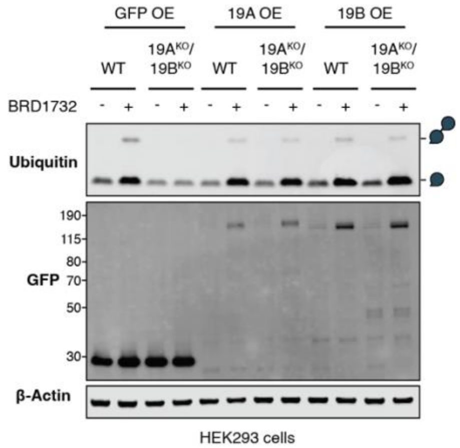

Supplementary Figure 5. Formation of BRD1732 ubiquitin conjugates is dependent on RNF19. Immunoblot of HEK293T WT and RNF19A/B double knockout cells transfected with GFP, RNF19A or RNF19B then treated for 6 hours with 5 μM BRD1732 or DMSO. Immunoblots are representative of four biological experiments.

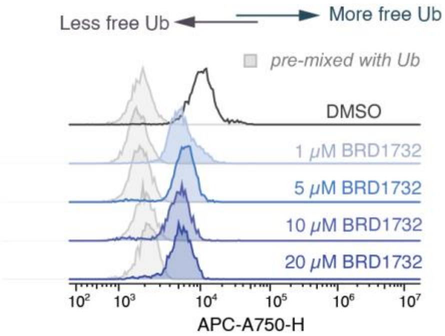

Supplementary Figure 6. BRD1732 treatment minimally reduces free ubiquitin in HEK293T cells. Flow cytometry of HEK293T cells treated with BRD1732 at indicated concentrations for 6 hours, stained with free-ubiquitin binder HA-tUI followed by Alexa Fluor 750-conjugated anti-HA antibody. Cells pre-incubated with excess free ubiquitin prior to staining (gray traces) served as a negative control. Immunoblots are representative of three biological experiments.

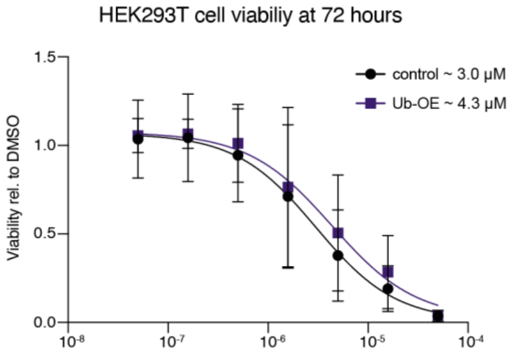

Supplementary Figure 7. Overexpression of ubiquitin does not alter the cytotoxicity of BRD1732 in HEK293T cells. HEK293T cells were transiently transfected with empty vector (control) or ubiquitin-overexpression vector (Ub-OE) and incubated for 48 hours. Cell viability was then measured after an additional 72-hour BRD1732 treatment. Mean ± s.d., n = 9 biological replicates representing three independent experiments.

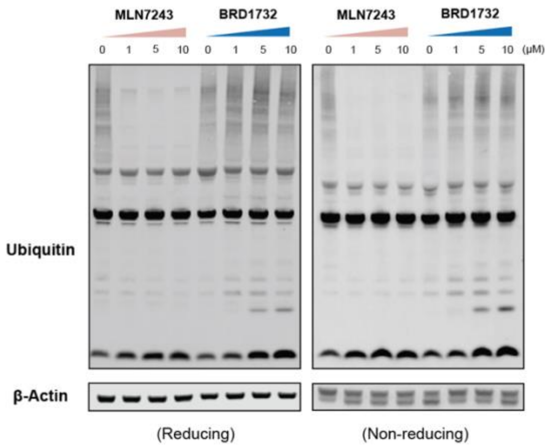

Supplementary Figure 8. MLN7243 does not induce low-molecular weight ubiquitin chains like BRD1732. Immunoblot following treatment of KP4 cells with indicated concentrations of BRD1732 or MLN7243 for 6 hours. Immunoblots are representative of four biological experiments.

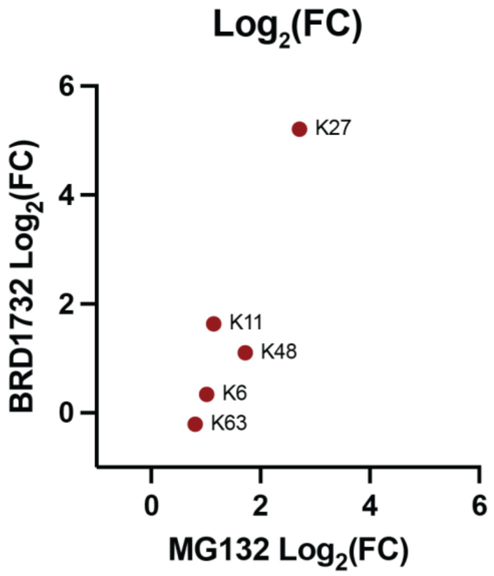

Supplementary Figure 9. BRD1732 induces dramatic accumulation of K27-linked ubiquitin chains. TMT proteomics dataset from **5a** analyzed for peptides corresponding to specific ubiquitin-ubiquitin linkages.

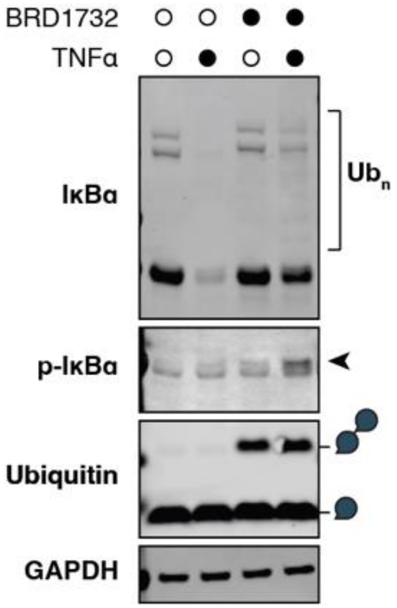

Supplementary Figure 10. BRD1732 inhibits TNFα-induced IκBα ubiquitination and degradation. Immunoblot analysis of lysates from KP4 cells pre-treated with DMSO or 5 μM BRD1732 (6 hours), followed by stimulation with TNFα (20 min). Immunoblots are representative of two biological experiments.

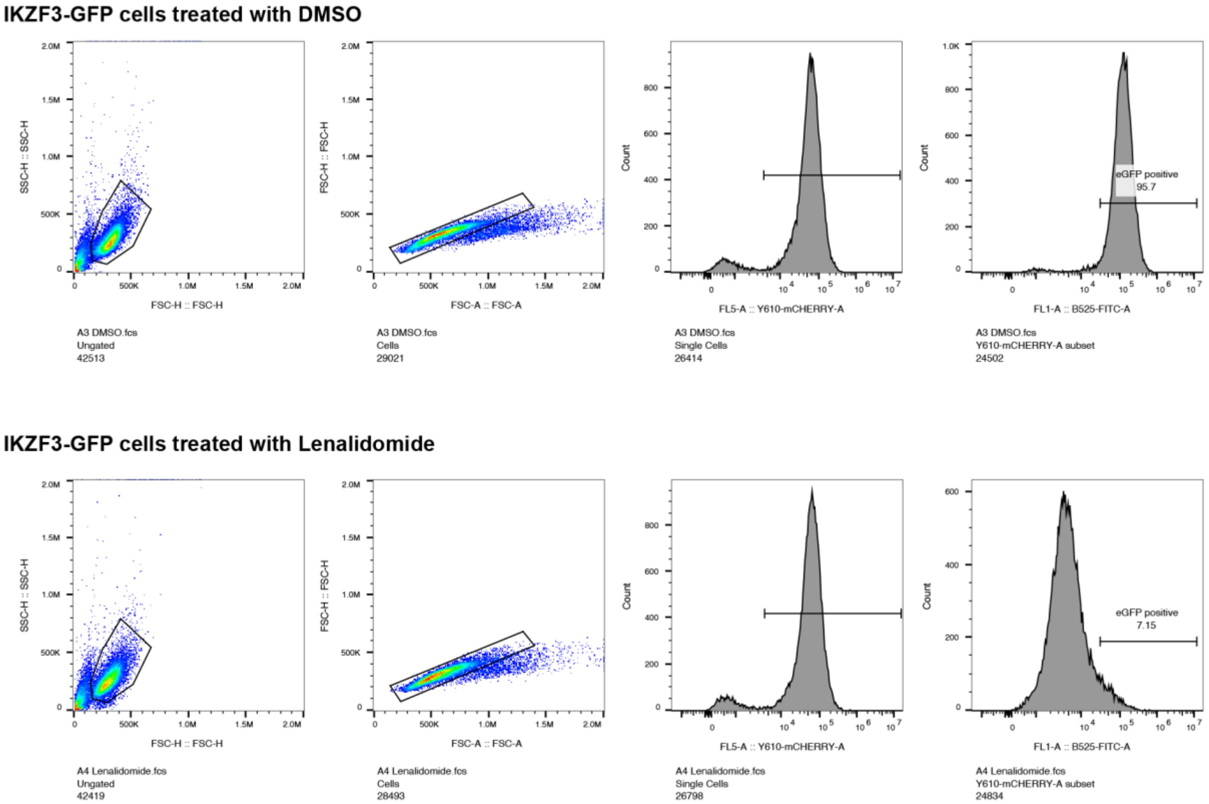

Supplementary Figure 11. Gating strategy for flow cytometry analysis of IKZF3-GFP degradation. HEK293T cells stably expressing IKZF3-GFP were treated with either DMSO (top) or 5 μM lenalidomide (bottom) for 6 h, and GFP levels were analyzed by flow cytometry. Cells were sequentially gated for size and granularity (FSC-H vs. SSC-H), singlets (FSC-A vs. FSC-H), and mCherry expression (Y610 channel) to identify the transduced population. GFP fluorescence (FITC channel) was quantified in the mCherry+ subset to determine the percentage of GFP-positive cells.

Supplementary Tables

Supplementary Table 1. Antibodies used in this study

Designation	Catalog number	Source	Dilution
anti-Ubiquitin, Mouse IgG	13-1600	Invitrogen	1:1000
anti-β-actin	4970S/3700S	Cell signaling	1:1000
Anti-GFP	2956S	Cell signaling	1:1000
anti-HA (Alexa Fluor® 750)	20818S	Cell signaling	1:500
anti-Ub-H2A-K119	8240S	Cell signaling	1:500
anti-E2T	12992S	Cell signaling	1:1000
anti-E2A/B	4944S	Cell signaling	1:1000
Anti-UBE2L3	3848S	Cell signaling	1:1000
anti-Mcl-1	94296S	Cell signaling	1:1000
anti-Nrf1	8052S	Cell signaling	1:1000
anti-p21	2947S	Cell signaling	1:1000
anti-p27	3686S	Cell signaling	1:1000
anti-p53	9282S	Cell signaling	1:1000
anti-GAPDH	97166S	Cell signaling	1:1000
anti-IκBα	2859T	Cell signaling	1:1000
anti-Phospho IκBα	2859S	Cell signaling	1:1000
anti-ZFAND5	MA5-26456	Invitrogen	1:1000
anti-ZFAND6	PA5-57083	Invitrogen	1:1000
Goat anti-Mouse IgG IRDye® 800CW	926-68070	LICOR	1:10000
Goat anti-Rabbit IgG IRDye® 680RD	926-32211	LICOR	1:10000

Supplementary Table 2. Plasmids used in this study

Plasmid	Source
eGFP, pLVX6	Gift from Zhenkun Lou
Human RNF19A, pLVX6	Gift from Zhenkun Lou
Human RNF19B, pLVX6	This study
Human RNF19A C316K, pLVX6	This study
Human RNF19A RBR2TM, pLVX6	This study
Human RNF19A RBR1TM, pLVX6	This study
Human RNF19A RBR, pLVX6	This study
Human ubiquitin, pLVX6	This study
Human UBB^{+1} with myc tag, pCMV-Myc	Gift from Nico Dantuma Addgene plasmid # 20254
Human ubiquitin with myc tag, pCMV-Myc	This study
HA-tUI, pET28	Gift from Robert Cohen Addgene plasmid # 122662

Supplementary Note

Chemical synthesis of compounds

Abbreviations

DCM – dichloromethane
DIBAL - diisobutylaluminium hydride
DIPEA - N,N-Diisopropylethylamine
DMF – Dimethylformamide
$NaHCO_3$ – Sodium bicarbonate
EtOAc – Ethyl acetate
Boc_2O – Boc anhydride
MeCN – Acetonitrile

***Synthesis of BRD1732 (1)*:**

General Scheme:

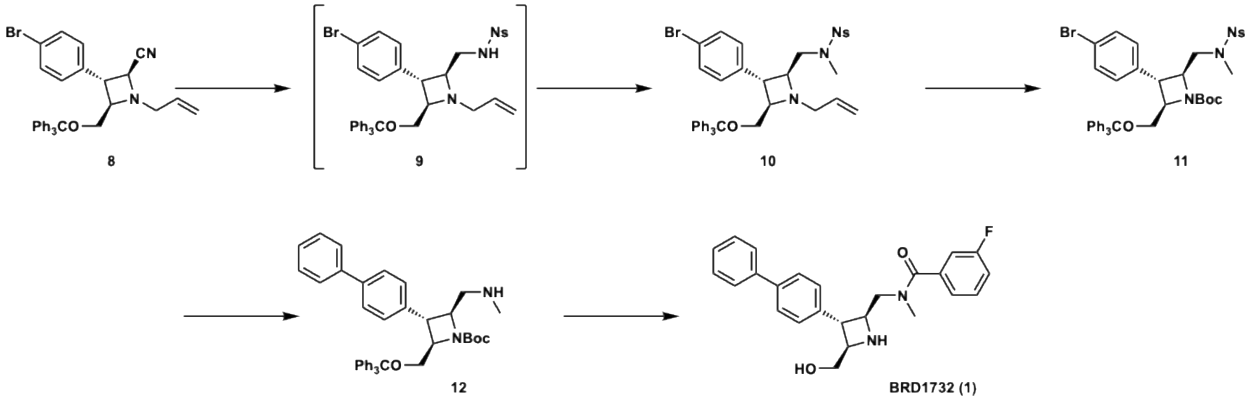

Step 1:

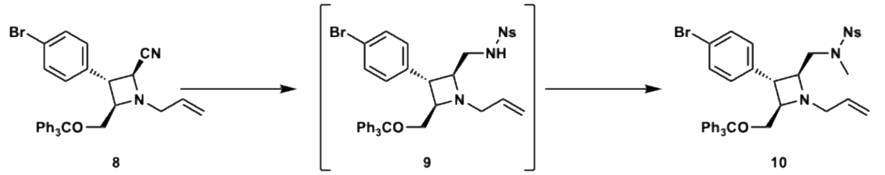

N-(((2S,3R,4R)-1-allyl-3-(4-bromophenyl)-4-((trityloxy)methyl)azetidin-2-yl)methyl)-N-methyl-4-nitrobenzenesulfonamide (10).

To a solution of (2S,3R,4R)-1-allyl-3-(4-bromophenyl)-4-((trityloxy)methyl)azetidine-2-carbonitrile (**8**) [JP-5-14, (Lowe *et al.*, 2012)] (2.9 g, 5.3 mmol, 1.0 equiv) in DCM (53 mL, 0.1 M) at 0 °C was added DIBAL-H (1 M in DCM) (31.8 mL, 31.8 mmol, 6.0 equiv) dropwise over

20 min. The reaction was slowly warmed to ambient temperature (23 °C) over 2 h. Upon complete reduction, the reaction was cooled to 0 °C and MeOH was added dropwise (*note: vigorous bubbling and significant exotherm observed*) until bubbling ceased. Next, a solution of saturated Rochelle's salt (potassium sodium tartrate in H_2O, 15 mL per 1.0 mmol of substrate **1**) was added dropwise over 5 min (*note: significant exotherm observed*), and the mixture was stirred at 0 °C for 2 h. The mixture was then extracted 4x with DCM, and combined organic layers were dried over Na_2SO_4, filtered, and removed *in vacuo* to afford crude primary amine as a thick yellow oil.

The resulting crude oil was reconstituted in DCM (53 mL, 0.1 M) and cooled to 0 °C before the addition of Et_3N (2.2 mL, 15.9 mmol, 3.0 equiv) and 2-nitrobenzenesulfonyl chloride (1.3 g, 5.83 mmol, 1.1 equiv). After complete conversion, the solvent was removed *in vacuo* and crude residue was pass through a column using (EtOAc/hexanes) to afford desired product as a white foaming solid (3.5 g, 4.7 mmol, 89%) which was directly used for the next step.

To a solution of **N-(((2S,3R,4R)-1-allyl-3-(4-bromophenyl)-4-((trityloxy)methyl)azetidin-2-yl)methyl)-4-nitrobenzenesulfonamide (9)** (2.7 g, 3.7 mmol 1.0 equiv) in DMF (15 mL) was added Cs_2CO_3 (1.8 g, 5.5 mmol, 1.5 equiv) followed by methyl iodide (255 µL, 4.1 mmol, 1.1 equiv) and allowed to stir for 16 h at ambient temperature. The reaction was then quenched by the addition of water and extracted 3x with EtOAc. The combined organic layers were dried over Na_2SO_4, filtered, and purified using flash column chromatography (EtOAc/hexanes) to afford desired product as a white foaming solid (2.54 g, 3.37 mmol, 91%). ^{1}H NMR (400 MHz, CDCl3) δ 7.93 (dd, J = 7.6, 1.4 Hz, 1H), 7.71 – 7.59 (m, 3H), 7.51 – 7.39 (m, 8H), 7.39 – 7.19 (m, 10H), 7.17 (d, J = 7.7 Hz, 2H), 5.94 – 5.76 (m, 1H), 5.24 (d, J = 17.0 Hz, 1H), 5.09 (d, J = 9.7 Hz, 1H), 3.58 – 3.41 (m, 2H), 3.36 – 3.18 (m, 6H), 2.98 (s, 3H). ^{13}C NMR (100 MHz, CDCl3) δ 148.31, 144.03, 139.31, 135.10, 133.46, 132.15, 131.48, 130.92, 129.57, 128.74, 127.81, 127.01, 124.09, 120.47, 118.20, 86.64, 69.05, 68.89, 66.82, 61.19, 53.75, 43.06, 36.79. Chemical Formula: C40H38BrN3O5S, $[M+H]^+$, Calculated:752.1788, Found:752.1834.

Step 2:

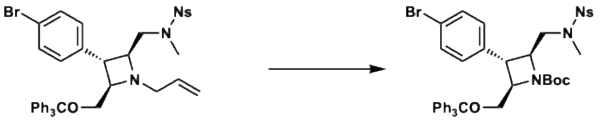

10 11

tert-butyl (2S,3R,4R)-3-(4-bromophenyl)-2-(((N-methyl-4-nitrophenyl)sulfonamido)methyl)-4-((trityloxy)methyl)azetidine-1-carboxylate (11).

To a solution of **10** (1.3 g, 1.73 mmol, 1.0 equiv) in 2:1 EtOH/DCM (15 mL) was added 1,3-dimethylbarbituric acid (406 mg, 2.60 mmol, 1.5 equiv) and $Pd(PPh_3)_4$ (200.1 mg, 0.13 mmol, 0.1 equiv). The mixture was left to stir at RT for 4 h, or until complete deallylation was observed, at which point Boc_2O (566 mg, 2.60 mmol 1.5 equiv) was added in a single portion. After complete conversion, the solvent was removed *in vacuo* and crude material was purified using flash column chromatography (EtOAc/hexanes) to afford desired product as a pale yellow foaming solid (1.1 g, 1.35 mmol, 78%). ^{1}H NMR (400 MHz, $CDCl_3$) δ 7.91 – 7.87 (m,1H), 7.71 – 7.67 (m, 1H), 7.64 – 7.59 (m, 2H), 7.50 – 7.45 (m, 8H), 7.35 – 7.30 (m, 6H), 7.28 – 7.24 (m, 3H), 7.20 (d, J = 8.4 Hz, 2H), 4.28 (dd, J = 11.5, 5.9 Hz, 1H), 4.23 – 4.16 (m, 1H), 3.78 (t, J = 6.4 Hz, 1H), 3.75 – 3.63 (m,

2H), 3.56 – 3.49 (m, 1H), 3.38 – 3.31 (m, 1H), 2.92 (s, 3H), 1.43 (s, 9H). ^{13}C NMR (100 MHz, $CDCl_3$) δ 157.35, 148.38, 143.84, 138.89, 133.53, 132.06, 131.76, 131.58, 130.80, 129.20, 128.76, 127.92, 127.10, 124.18, 120.94, 86.80, 80.55, 66.09, 64.06, 53.34, 42.14, 36.38, 28.33. Chemical Formula: C42H42BrN3O7S, [M^+Na], Calculated: 834.1892, Found: 834.1899.

Step 3:

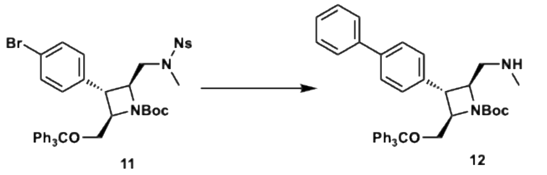

tert-butyl (2S,3R,4R)-3-([1,1'-biphenyl]-4-yl)-2-((methylamino)methyl)-4-((trityloxy)methyl)azetidine-1-carboxylate (12).

A reaction vessel charged with **11** (1.1 g, 1.35 mmol, 1.0 equiv) and phenylboronic acid (329 mg, 2.71 mmol, 2.0 equiv) was evacuated and backfilled 3x with N_2. Degassed solutions of dioxane (13.5 mL, 0.1 M) and 0.5 M K_3PO_4 (8.1 mL, 4.05 mmol, 3.0 equiv) were added, followed by XPhos Pd G3 (117 mg, 0.135 mmol, 0.10 equiv), and the mixture was heated to 100 °C. After 1 h or upon complete conversion, the mixture was quenched with saturated NH_4Cl and extracted 3x with EtOAc. The combined organic layers were dried over Na_2SO_4, filtered, and removed *in vacuo* to afford a dark brown residue.

The residue was reconstituted in DMF (10 mL), followed by the addition of K_2CO_3 (1.12 g, 8.1 mmol, 6 equiv) and thiophenol (413 µL, 4.05 mmol, 3 equiv). The reaction was allowed to stir at ambient temperature or until completion, at which point saturated $NaHCO_3$ was added to quench the reaction. The aqueous layer was extracted 3x with EtOAc, and combined organic layers were dried over Na_2SO_4, filtered, and removed *in vacuo*. The crude material was purified using flash column chromatography (MeOH/DCM) to afford desired product as an off white solid (750 mg, 1.20 mmol, 89%). ^1H NMR (400 MHz, $CDCl_3$) δ 7.64 – 7.58 (m, 4H), 7.54 – 7.46 (m, 8H), 7.40 – 7.36 (m, 4H), 7.36 – 7.33 (m, 5H), 7.31 – 7.27 (m, 3H), 4.51 (s, 1H), 4.26 (s, 1H), 3.61 (t, J = 6.7 Hz, 1H), 3.54 (dd, J = 9.9, 5.4 Hz, 1H), 3.41 (dd, J = 10.0, 3.2 Hz, 1H), 3.19 (dd, J = 12.4, 7.1 Hz, 1H), 3.07 (dd, J = 12.4, 3.6 Hz, 1H), 2.56 (s, 3H), 1.45 (s, 9H). ^{13}C NMR (100 MHz, CDCl3) δ 157.92, 143.96, 140.77, 140.02, 139.24, 134.06, 128.84, 128.81, 127.88, 127.68, 127.38, 127.35, 127.11, 127.09, 86.79, 80.43, 66.43, 64.02, 56.59, 41.58, 36.24, 28.38. Chemical Formula: C42H44N2O3, $[M+H]^+$, Calculated:625.3425, Found: 625.3474.

Step 4:

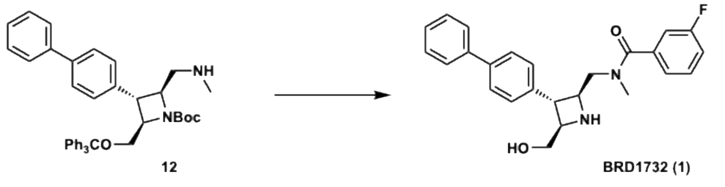

N-(((2S,3R,4R)-3-([1,1'-biphenyl]-4-yl)-4-(hydroxymethyl)azetidin-2-yl)methyl)-3-fluoro-N-methylbenzamide (BRD1732, 1).

To a solution of **12** (424 g, 0.68 mmol, 1.0 equiv) in DCM (5 mL) and cooled to 0 °C. Et_3N (284 µL, 2.04 mmol, 3.0 equiv) and 3-fluorobenzoyl chloride (99 µL, 0.081 mmol, 1.2 equiv) were added slowly. The reaction was slowly warmed to ambient temperature (23 °C) over 1 h. Upon complete conversion, the reaction was quenched with saturated NH_4Cl and extracted 3x with DCM. The combined organic layers were dried over Na_2SO_4, filtered, and removed *in vacuo*, and crude residue.

The residue was reconstituted in 4 M HCl in dioxane (1.7 mL, 6.8 mmol, 10 equiv), followed by the addition of Et_3SiH (163 µL, 1.02 mmol, 1.5 equiv). After 1 h, solvent was removed *in vacuo* and crude material was purified using flash column chromatography (MeOH/DCM) to afford desired product as a pale yellow residue (260 mg, 0.64 mmol, 94%). ^{1}H NMR (400 MHz, CDCl3) δ 7.67 – 7.55 (m, 4H), 7.54 – 7.42 (m, 4H), 7.42 – 7.35 (m, 2H), 7.21 – 7.07 (m, 3H), 4.88 (s, 1H), 4.68 – 4.43 (m, 3H), 4.34 (dd, *J* = 13.1, 6.6 Hz, 1H), 4.13 (t, *J* = 8.1 Hz, 1H), 3.99 (d, *J* = 12.1 Hz, 1H), 3.79 (d, *J* = 12.1 Hz, 1H), 3.62 (d, *J* = 14.5 Hz, 1H), 3.00 (s, 3H). ^{13}C NMR (100 MHz, $CDCl_3$) δ 171.82, 163.59, 161.12, 141.40, 140.23, 137.20 (d, *J* = 6.2 Hz), 135.09, 130.51 (d, *J* = 7.3 Hz), 128.91, 128.14, 127.93, 127.65, 127.07, 122.95, 117.13 (d, *J* = 20.7 Hz), 114.43 (d, *J* = 22.9 Hz), 65.90, 61.57, 59.12, 49.89, 41.81, 39.61. Chemical Formula: C25H25FN2O2, [M+H]$^+$, Calculated: 405.1973, Found: 405.2011.

Synthesis of BRD1732 isomers (2, 3) :

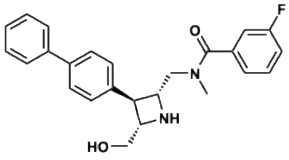

BRD-E (2)

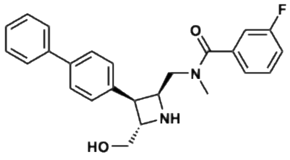

BRD-D (3)

N-(((2R,3S,4S)-3-([1,1'-biphenyl]-4-yl)-4-(hydroxymethyl)azetidin-2-yl)methyl)-3-fluoro-N-methylbenzamide (BRD-E, 2).

The enantiomer of BRD1732 was synthesized from (2R,3S,4S)-1-allyl-3-(4-bromophenyl)-4-((trityloxy)methyl)azetidine-2-carbonitrile (prepared as in Lowe *et al.*, 2012) in a manner analogous to BRD1732. ^{1}H NMR (400 MHz, CD_3OD) δ 7.65 – 7.59 (m, 4H), 7.49 (d, J = 7.8 Hz, 2H), 7.46 – 7.41 (m, 3H), 7.38 – 7.32 (m, 2H), 7.19 – 7.12 (m, 2H), 6.95 (d, J = 7.6 Hz, 1H), 6.89 (d, J = 9.0 Hz, 1H), 4.09 (dd, J = 8.7, 4.4 Hz, 1H), 4.00 (dd, J = 13.7, 6.7 Hz, 1H), 3.78 – 3.62 (m, 4H), 3.55 (s, 1H), 2.95 (s, 3H). ^{13}C NMR (100 MHz, $CDCl_3$) δ 172.72 (s), 163.64 (s), 161.17 (s), 141.70 (s), 140.14 (s), 136.50 (d, *J* = 6.8 Hz), 134.58 (s), 130.61 (d, *J* = 8.2 Hz), 128.93 (s), 128.03 (s), 127.96 (s), 127.73 (s), 127.07 (s), 122.62 (s), 117.53 (d, *J* = 21.0 Hz), 114.18 (d, *J* = 23.2 Hz), 65.29 (s), 61.48 (s), 58.72 (s), 49.96 (s), 41.70 (s), 39.26 (s). Molecular formula: $C_{25}H_{25}FN_2O_2$. Calculated mass: 404.19. ESIMS m/z 405.0 $(M+H)^+$

N-(((2R,3R,4S)-3-([1,1'-biphenyl]-4-yl)-4-(hydroxymethyl)azetidin-2-yl)methyl)-3-fluoro-N-methylbenzamide (BRD-D, 3).

The (2R,3R,4S) diastereomer of BRD1732 was synthesized from (2R,3R,4S)-1-allyl-3-(4-bromophenyl)-4-((trityloxy)methyl)azetidine-2-carbonitrile (prepared as in Lowe *et al.*, 2012) in a manner analogous to BRD1732. ^{1}H NMR (400 MHz, DMSO d6) δ 8.30 (s, 1H), 7.75 – 7.64 (m, 2H), 7.63 – 7.57 (m, 2H), 7.55 – 7.51 (m, 4H), 7.50 – 7.24 (m, 3H), 7.22 – 7.12 (m, 2H), 4.36 (m, 1H), 4.05 (m, 1H), 3.81 – 3.38 (m, 4H), 3.22 – 3.09 (m, 1H), 2.88 (s, 1.5H), 2.74 (s, 1.5H). ^{13}C NMR (100 MHz, $CDCl_3$) δ 173.60 (s), 163.64 (s), 161.17 (s), 141.62 (s), 139.92 (s), 135.94 (d, *J* = 8.2 Hz), 132.26 (s), 130.62 (d, *J* = 7.5 Hz), 128.98 (s), 128.77 (s), 127.98 (s), 127.85 (s), 127.03 (s), 122.96 (d, *J* = 2.6 Hz), 117.84 (d, *J* = 21.0 Hz), 114.44 (d, *J* = 23.4 Hz), 64.28 (s), 59.81 (s), 59.22 (s), 49.26 (s), 40.24 (s), 38.76 (s). Molecular formula: $C_{25}H_{25}FN_2O_2$. Calculated mass: 404.19. ESIMS m/z 405.5 $(M+H)^+$.

Synthesis of BRD1732 analogs (4-7):

General scheme of BRD1732 analogs synthesis:

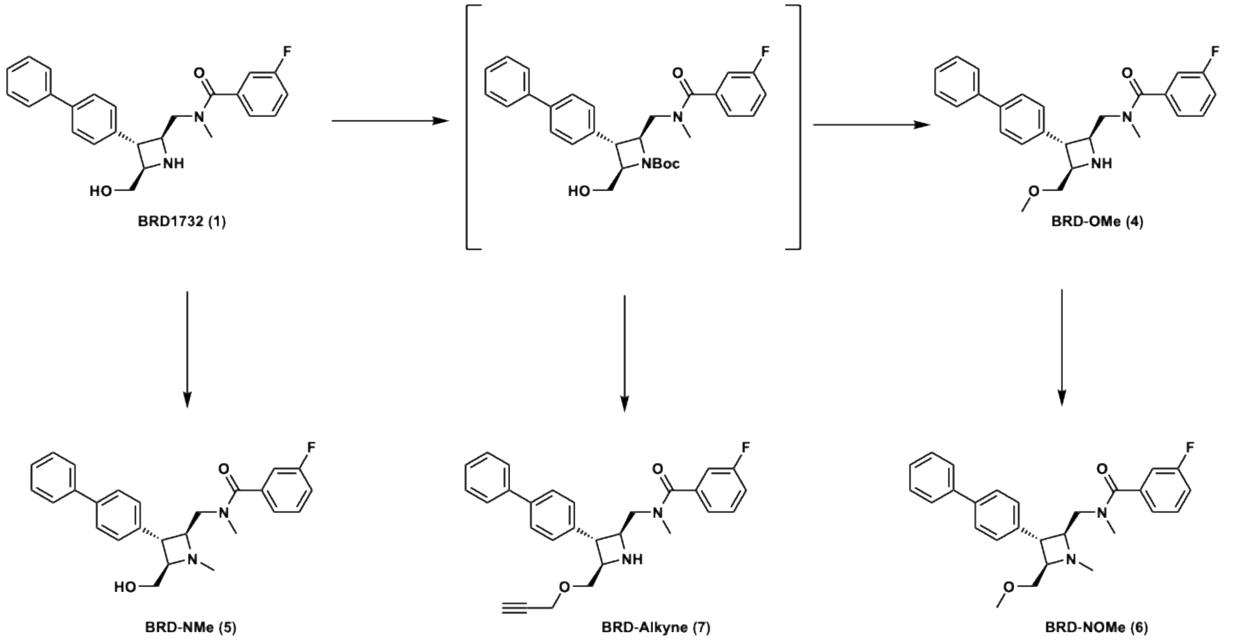

Synthesis of BRD-OMe (4):

N-(((2S,3R,4R)-3-([1,1'-biphenyl]-4-yl)-4-(methoxymethyl)azetidin-2-yl)methyl)-3-fluoro-N-methylbenzamide (BRD-OMe, 4).

The **BRD1732** (100 mg, 0.25 mmol, 1.0 equiv) was dissolved in DCM (5 mL), followed by addition of TEA (70 µL, 0.5 mmol, 2 equiv) and Boc_2O (65 mg, 0.3 mmol 1.2 equiv). After complete conversion, the solvent was removed *in vacuo* and crude material was resuspended in MeCN, followed by addition of 60 % NaH on mineral oil (18 mg, 0.75 mmol, 3.0 equiv) and MeI (156 µL, 2.5 mmol, 5 equiv) and left to stir at RT for 16 hr. After complete conversion the reaction mixture was concentrated and followed by adding saturated solution of $NaHCO_3$ and extracting the aqueous layer 3x with DCM. The combined organic layers were dried over Na_2SO_4, filtered, and removed *in vacuo* to afford a brown residue.

The residue was reconstituted in 4 M HCl in dioxane (1.0 mL, 4.0 mmol, 16 equiv), After 1 h, solvent was removed *in vacuo* and crude material was purified using flash column chromatography (MeOH/DCM) to afford desired product as a pale yellow solid (80 mg, 0.19 mmol, 76%). ^{1}H NMR (400 MHz, $CDCl_3$) δ 7.66 (d, J = 8.1 Hz, 2H), 7.61 – 7.57 (m, 2H), 7.53 (d, J = 8.1 Hz, 2H), 7.50 – 7.45 (m, 2H), 7.43 – 7.37 (m, 2H), 7.24 (d, J = 7.6 Hz, 1H), 7.20 – 7.14 (m, 2H), 5.05 (s, 1H), 4.71 (s, 1H), 4.42 (dd, J = 14.8, 9.1 Hz, 1H), 4.06 (t, J = 8.7 Hz, 1H), 3.74 – 3.64 (m, 2H), 3.59 (d, J = 15.0 Hz, 1H), 3.54 (s, 3H), 3.05 (s, 3H). ^{13}C NMR (100 MHz, $CDCl_3$) δ 173.18, 163.68, 161.20, 141.76, 140.15, 136.43 (d, J = 7.1 Hz), 134.72, 130.50 (d, J = 8.0 Hz), 128.94, 128.14, 128.08, 127.75, 127.08, 122.91 (d, J = 3.0 Hz), 117.57 (d, J = 21.0 Hz), 114.48

(d, J = 23.2 Hz), 68.49, 62.50, 61.19, 59.50, 49.89, 42.02, 39.07. Chemical Formula: C26H27FN2O2, $[M+H]^+$, Calculated: 419.2129, Found:419.2193.

Synthesis of BRD-OMe (5):

N-(((2S,3R,4R)-3-([1,1'-biphenyl]-4-yl)-4-(hydroxymethyl)azetidin-2-yl)methyl)-3-fluoro-N-methylbenzamide (BRD-NMe, 5).

The **BRD1732** (46 mg, 0.114 mmol, 1.0) was dissolved in MeCN (2 mL) followed by addition of K_2CO_3 (24 mg, 0.170 mmol, 1.5 equiv) and MeI (14 µL, 0.228 mmol, 2 equiv) and left to stir. The reaction was monitored by LCMS, after complete conversion the reaction mixture was concentrated and crude material was purified material was purified by HPLC to afford the desired product as an off-white solid (25 mg, 0.060 mmol, 52%). ^{1}H NMR (400 MHz, $CDCl_3$) δ 7.66 (d, J = 8.0 Hz, 2H), 7.61 – 7.56 (m, 2H), 7.48 (t, J = 7.5 Hz, 2H), 7.44 – 7.33 (m, 4H), 7.18 – 7.12 (m, 1H), 7.06 (d, J = 7.6 Hz, 1H), 6.98 (d, J = 8.3 Hz, 1H), 4.83 (dd, J = 13.9, 7.4 Hz, 1H), 4.19 – 3.94 (m, 6H), 3.16 (s, 3H), 3.08 (s, 3H). ^{13}C NMR (100 MHz, CDCl3) δ 171.71, 163.73, 161.25, 141.83, 139.99, 136.65 (d, J = 7.2 Hz), 134.14, 130.55 (d, J = 7.9 Hz), 128.97, 128.20, 127.85, 127.25, 127.09, 122.62 (d, J = 2.7 Hz), 117.66 (d, J = 21.1 Hz), 114.31 (d, J = 23.1 Hz), 76.24, 69.19, 58.83, 50.14, 41.87, 41.03, 40.46. Chemical Formula: C26H27FN2O2, $[M+H]^+$, Calculated: 419.2129, Found: 419.2193.

Synthesis of BRD-NOMe (6):

N-(((2S,3R,4R)-3-([1,1'-biphenyl]-4-yl)-4-(methoxymethyl)-1-methylazetidin-2-yl)methyl)-3-fluoro-N-methylbenzamide (BRD-NOMe, 6).

The **BRD-OMe** (29 mg, 0.069 mmol, 1.0 equiv) was dissolved in MeCN (2 mL) followed by addition of K_2CO_3(19.1 mg, 0.138 mmol, 2.0 equiv) and MeI (21.5 µL, 0.345 mmol, 5 equiv) and left to stir at RT. The reaction was monitored by LCMS, after complete conversion the reaction mixture was concentrated, and crude material was purified by using HPLC to afford desired product as a white solid (21 mg, 0.049 mmol, 70%). ^{1}H NMR (400 MHz, $CDCl_3$) δ 7.65 (d, J = 8.0 Hz, 2H), 7.60 – 7.55 (m, 2H), 7.48 (t, J = 7.5 Hz, 2H), 7.43 – 7.37 (m, 3H), 7.36 – 7.31 (m, 1H), 7.14 – 7.09 (m, 1H), 6.94 (d, J = 7.5 Hz, 1H), 6.87 (d, J = 8.5 Hz, 1H), 4.82 (dd, J = 14.7, 6.6 Hz, 1H), 4.25 – 4.02 (m, 5H), 3.78 (d, J = 8.5 Hz, 1H), 3.44 (s, 3H), 3.12 (s, 3H), 3.08 (s, 3H). ^{13}C NMR (100 MHz, $CDCl_3$) δ 171.44, 163.69, 161.21, 141.92, 140.01, 136.78 (d, J = 6.7 Hz), 133.76, 130.45 (d, J = 8.0 Hz), 128.96, 128.18, 127.83, 127.41, 127.09, 122.46 (d, J = 2.9 Hz), 117.48 (d, J = 21.0 Hz), 114.19 (d, J = 23.0 Hz), 73.90, 69.84, 69.61, 59.38, 49.24, 42.36, 42.07, 40.28. Chemical Formula: C27H29FN2O, $[M+H]^+$, Calculated: 433.2286, Found: 443.2230.

Synthesis of BRD-alkyne (7):

N-(((2S,3R,4R)-3-([1,1'-biphenyl]-4-yl)-4-((prop-2-yn-1-yloxy)methyl)azetidin-2-yl)methyl)-3-fluoro-N-methylbenzamide (BRD-alkyne, 7)

The **BRD1732** (30 mg, 0.074 mmol, 1.0 equiv) was dissolved in DCM (2 mL), followed by addition of TEA (21 µL, 0.148 mmol, 2 equiv) and Boc_2O (19 mg, 0.088 mmol 1.2 equiv). After complete conversion, the solvent was removed *in vacuo* and crude material was resuspended in MeCN, followed by addition of 60 % NaH on mineral oil (5.3 mg, 0.22 mmol, 3.0 equiv) and Propargyl bromide (20 µL, 0.22 mmol, 3 equiv) and left to stir at RT for 16 hr. After complete conversion the reaction mixture was concentrated and followed by adding saturated solution of $NaHCO_3$ and extracting the aqueous layer 3x with DCM. The combined organic layers were dried over Na_2SO_4, filtered, and removed *in vacuo* to afford a brown residue.

The residue was reconstituted in 4 M HCl in dioxane (370 µL, 1.48 mmol, 20 equiv), After 1 h, solvent was removed *in vacuo* and crude material was purified by HPLC to obtain the desired product as an off-white solid (20 mg, 0.045 mmol, 61%). ^{1}H NMR (400 MHz, $CDCl_3$) δ 7.67 (d, *J* = 8.1 Hz, 2H), 7.62 – 7.58 (m, 2H), 7.54 (d, *J* = 8.1 Hz, 2H), 7.48 (t, *J* = 7.5 Hz, 2H), 7.45 – 7.36 (m, 2H), 7.27 (d, *J* = 7.6 Hz, 1H), 7.24 – 7.14 (m, 2H), 5.11 (s, 1H), 4.78 (s, 1H), 4.52 – 4.41 (m, 2H), 4.32 (dd, *J* = 16.1, 2.1 Hz, 1H), 4.08 (t, *J* = 8.7 Hz, 1H), 3.96 – 3.81 (m, 2H), 3.54 (d, *J* = 14.5 Hz, 1H), 3.07 (s, 3H), 2.58 (s, 1H). ^{13}C NMR (100 MHz, $CDCl_3$) δ 173.37, 163.69, 161.21, 141.86, 140.12, 136.27 (d, *J* = 7.0 Hz), 134.56, 130.52 (d, *J* = 8.0 Hz), 128.95, 128.16, 128.13, 127.77, 127.09, 122.97 (d, *J* = 3.0 Hz), 117.67 (d, *J* = 21.0 Hz), 114.53 (d, *J* = 23.2 Hz), 78.22, 76.38, 65.53, 62.32, 61.18, 58.93, 50.02, 42.06, 39.07. Chemical Formula: C28H27FN2O2, $[M+H]^+$, Calculated: 443.2129, Found: 443.2176.

NMR Spectra

Compound 10 ^1H NMR ($CDCl_3$)

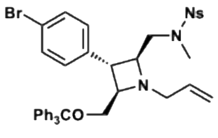

10

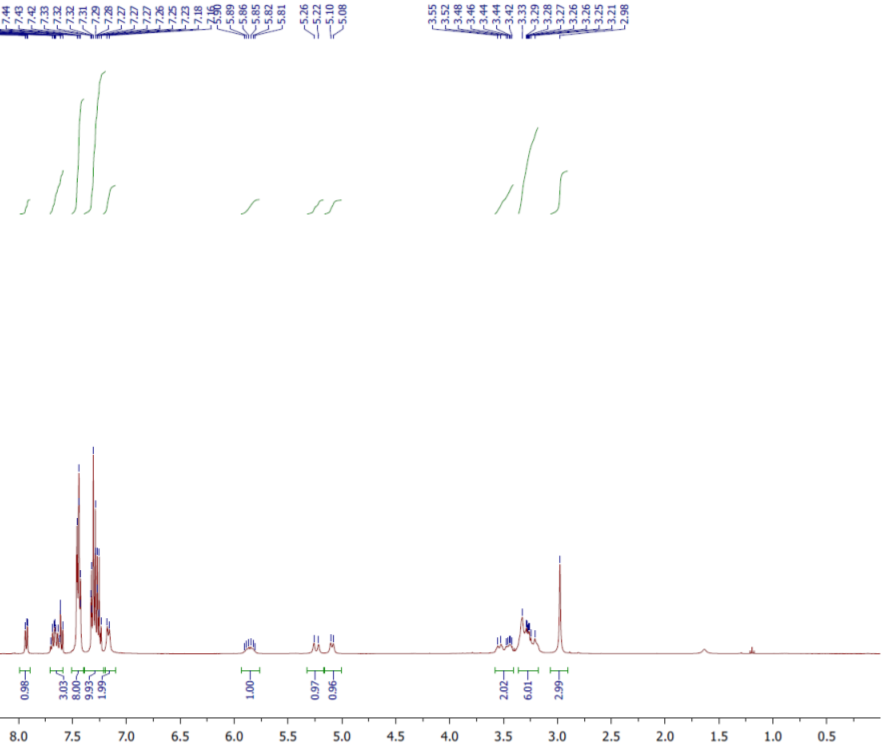

Compound 10 ^{13}C NMR ($CDCl_3$)

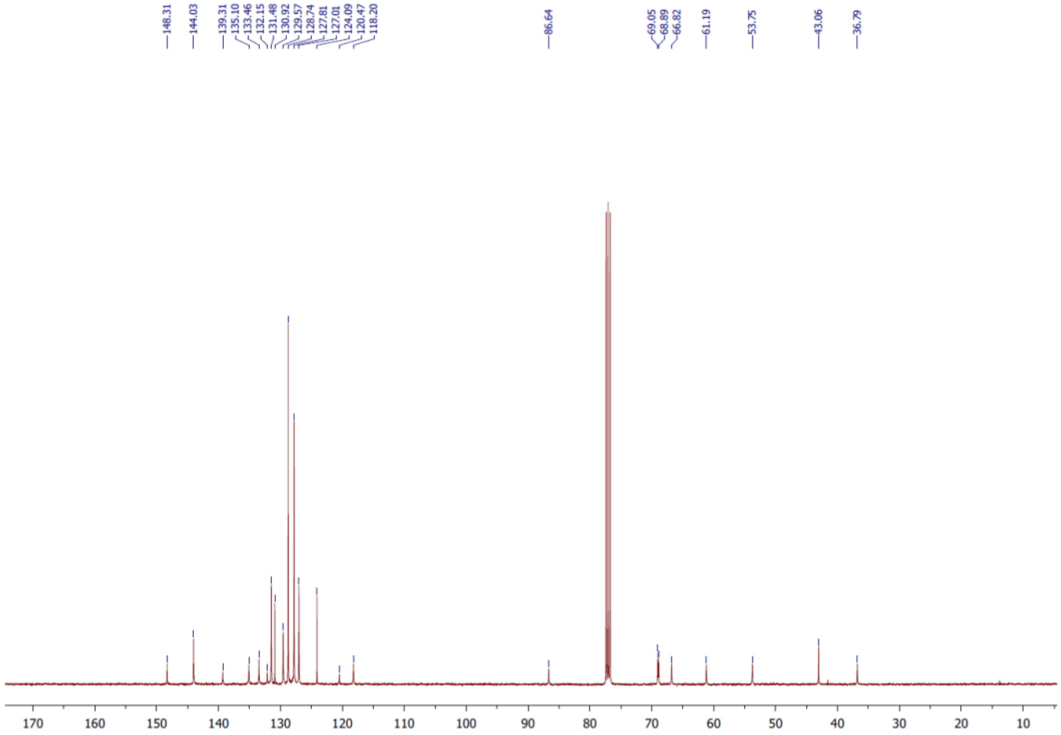

Compound 11 ^{1}H NMR ($CDCl_3$)

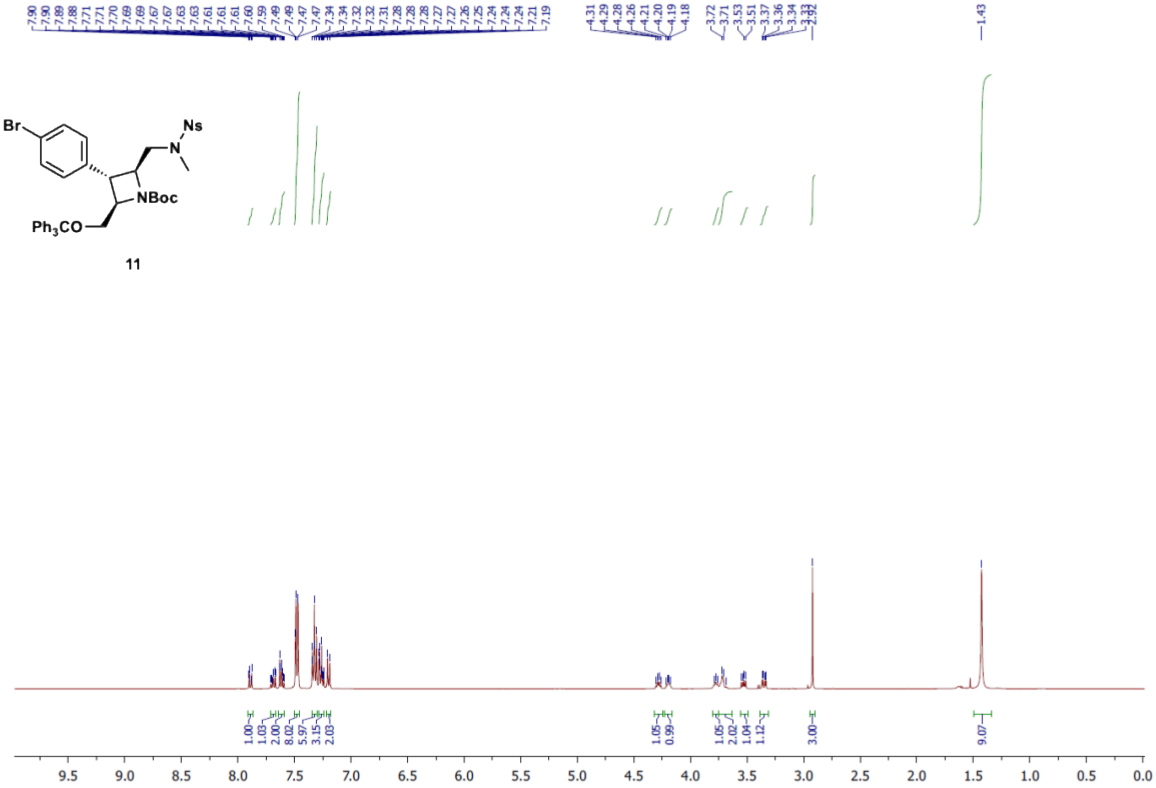

Compound 11 ^{13}C NMR ($CDCl_3$)

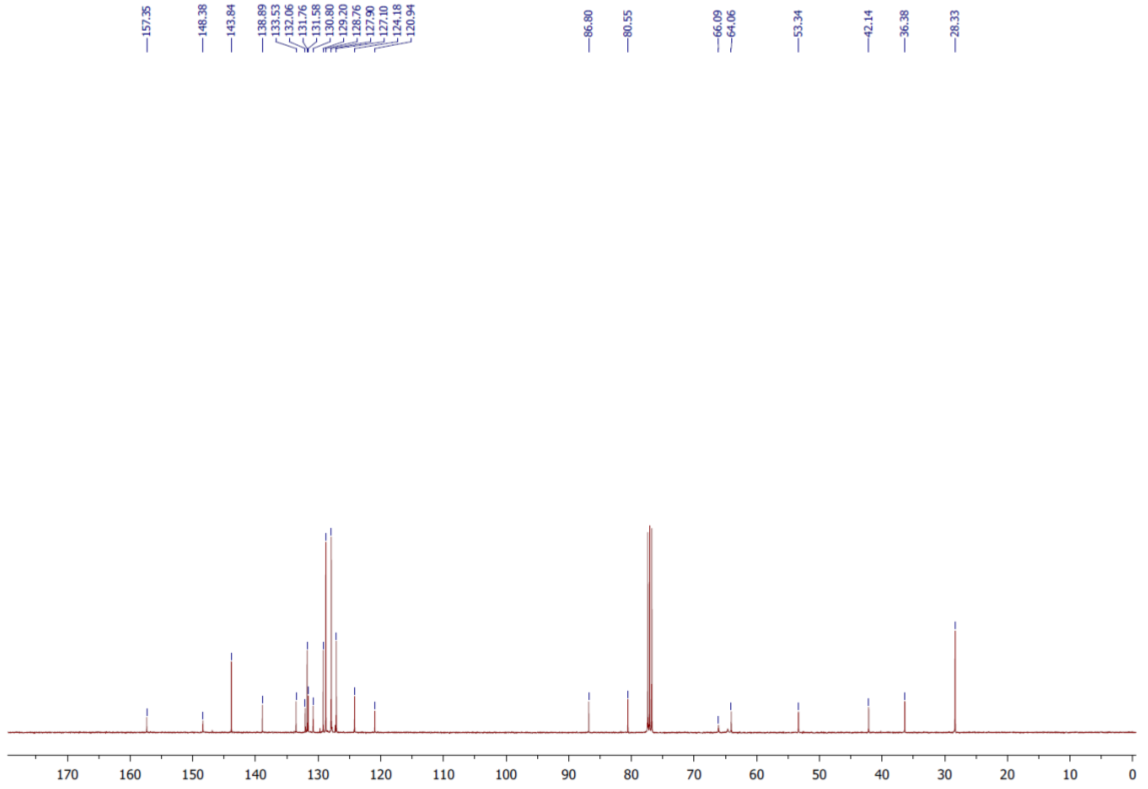

Compound 12 ^{1}H NMR ($CDCl_3$)

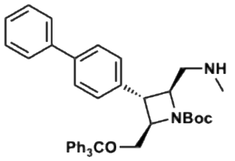

12

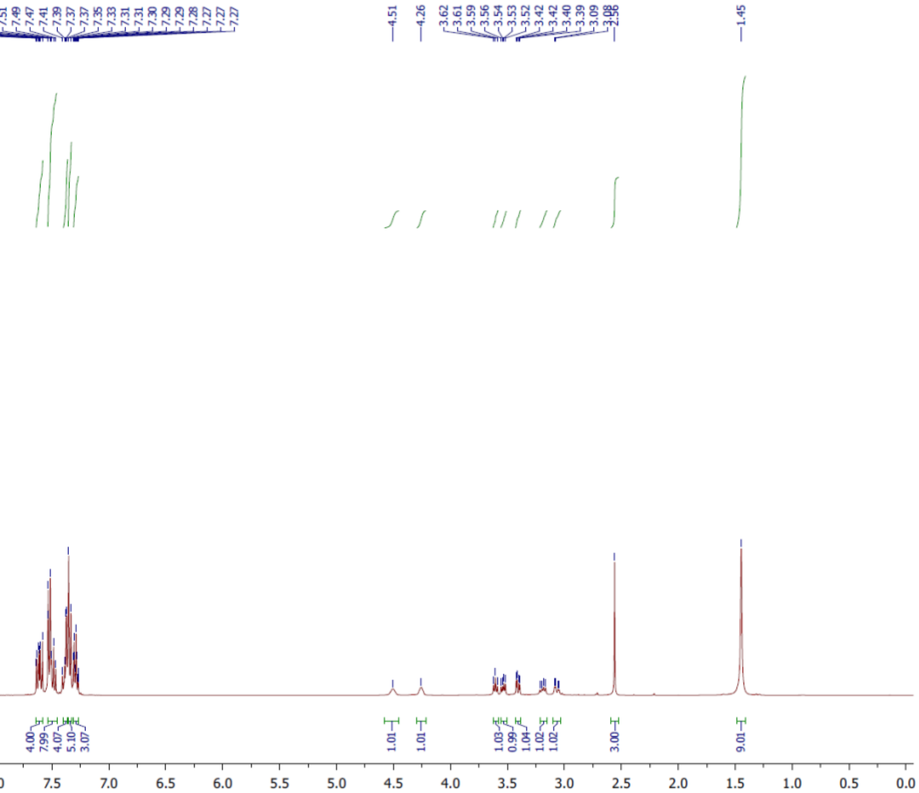

Compound 12 ^{13}C NMR ($CDCl_3$)

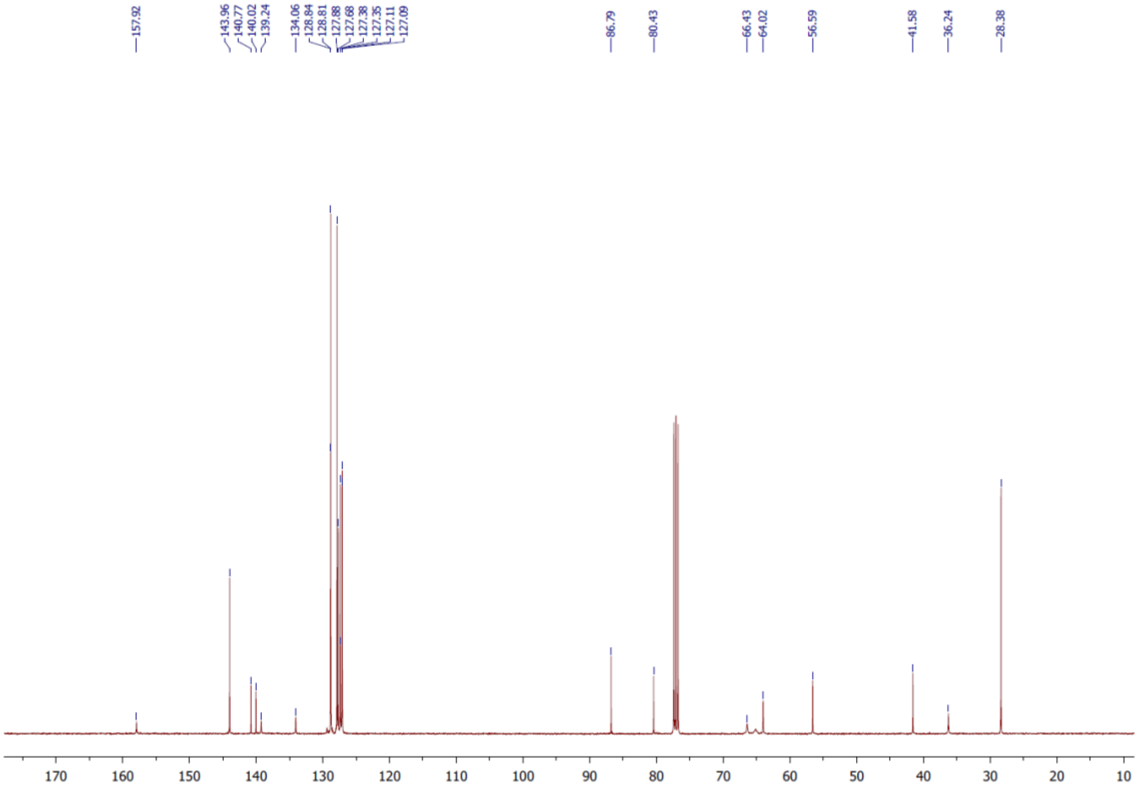

BRD1732 (1) ^{1}H NMR ($CDCl_3$)

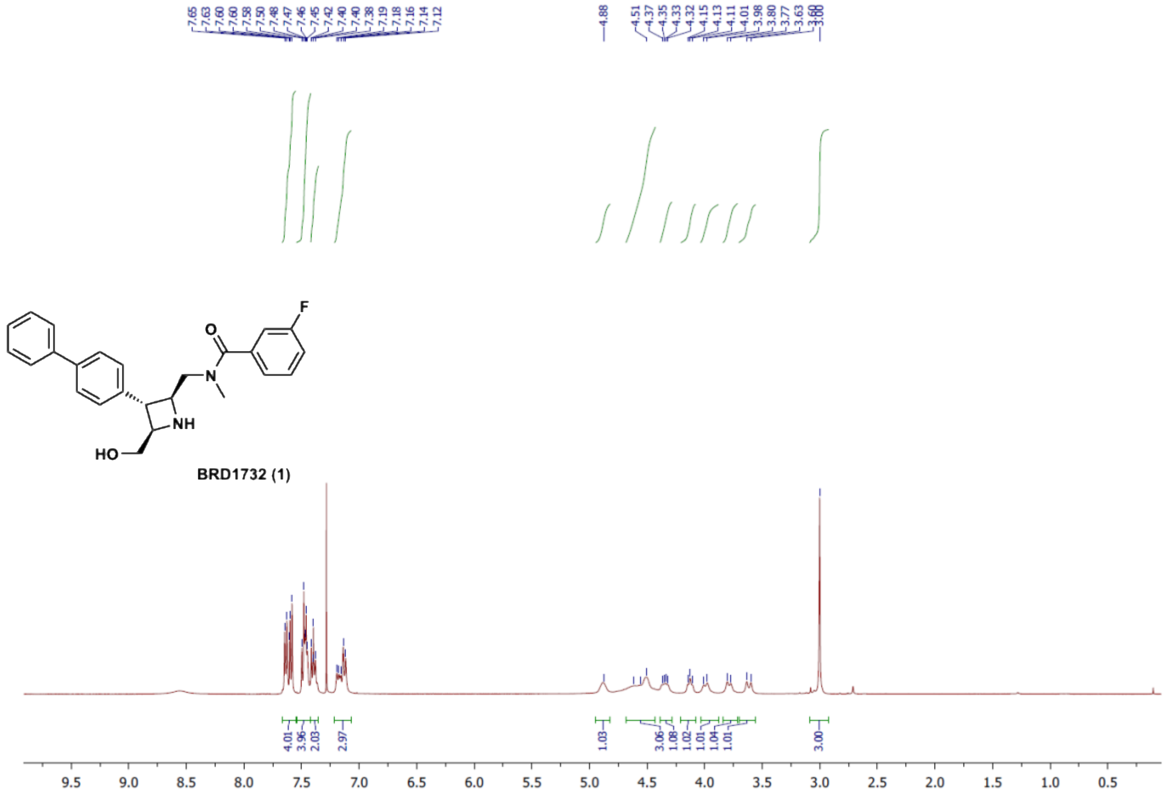

BRD1732 (1) ^{13}C NMR ($CDCl_3$)

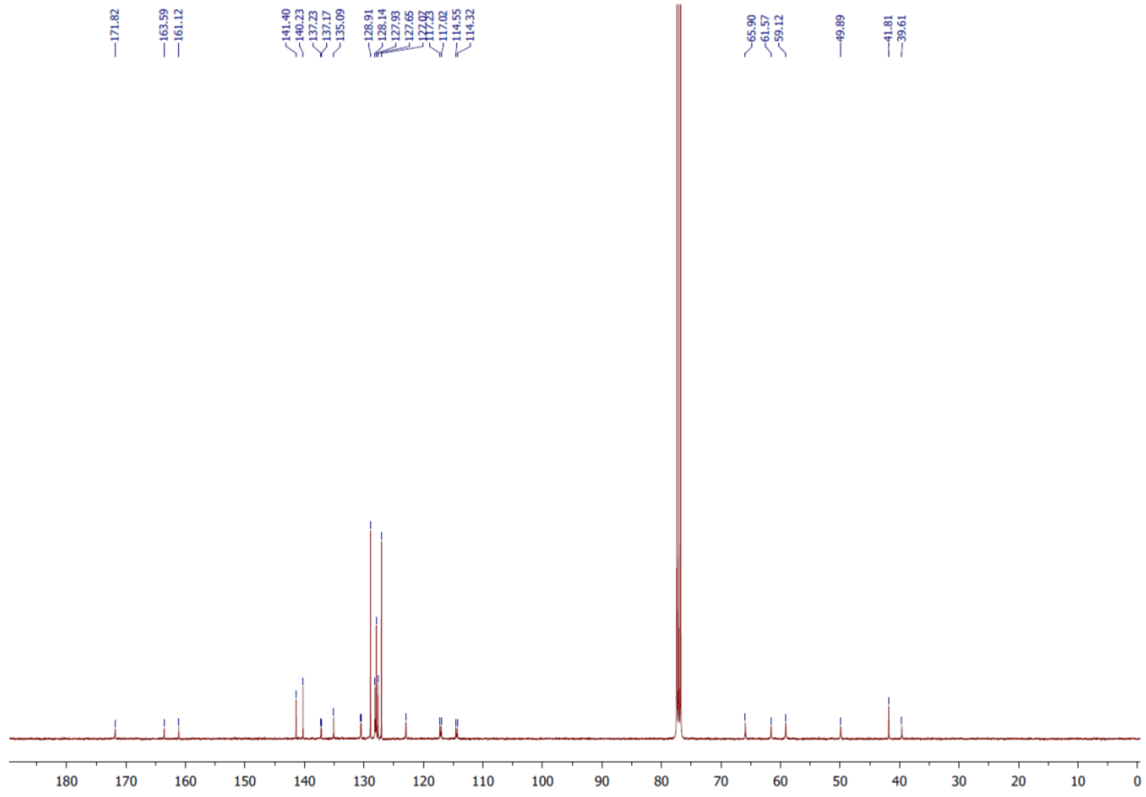

BRD-OMe (4) ^{1}H NMR ($CDCl_3$)

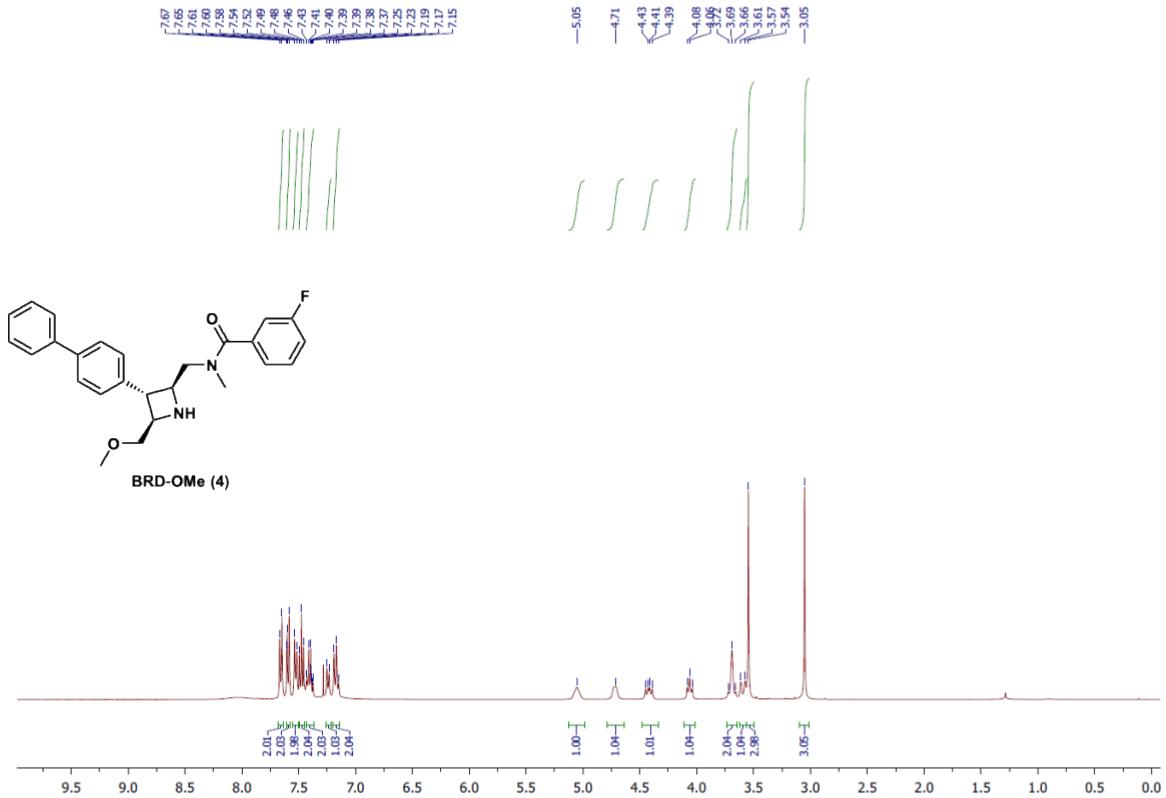

BRD-OMe (4)

BRD-OMe (4) ^{13}C NMR ($CDCl_3$)

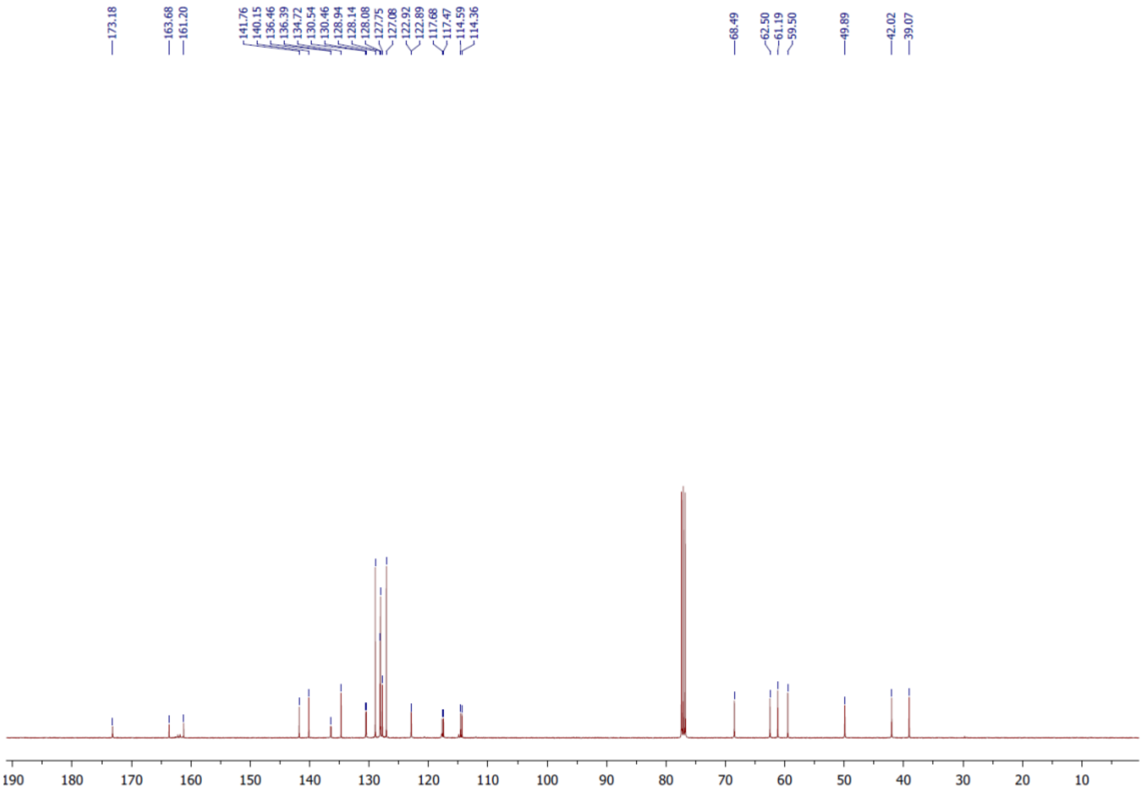

BRD-NMe (5) ^{1}H NMR ($CDCl_3$)

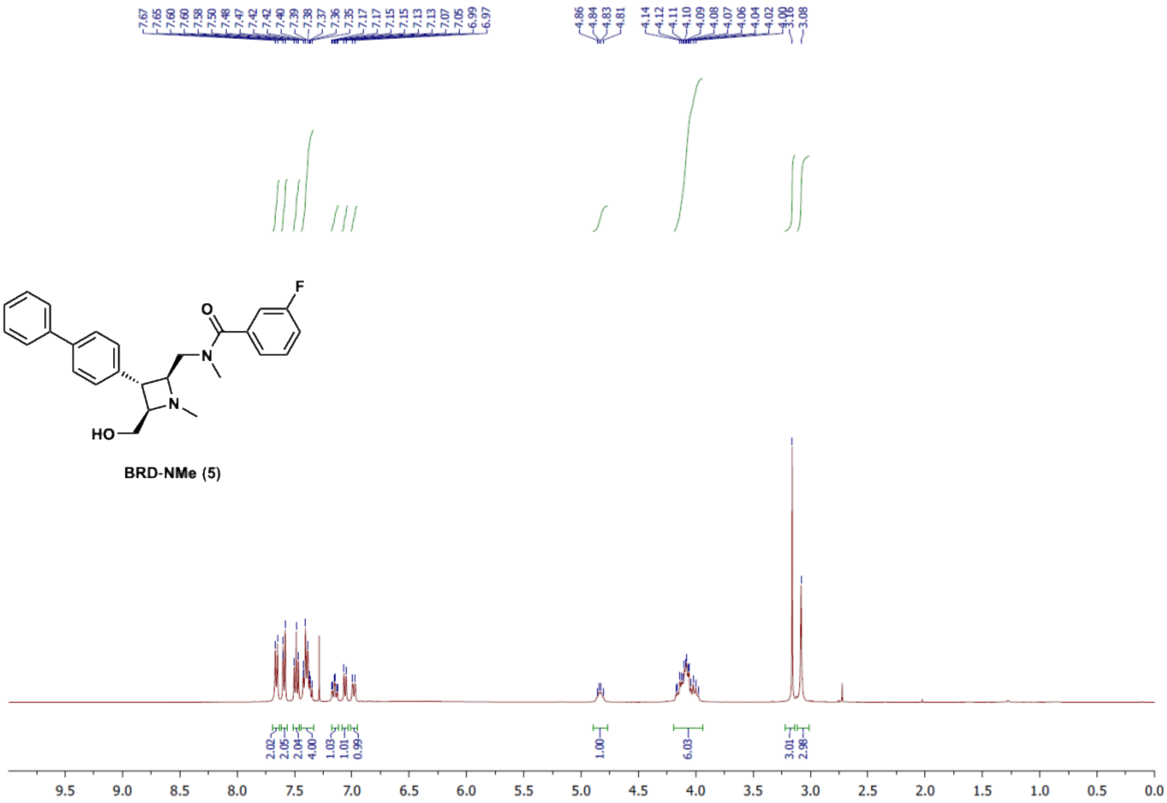

BRD-NMe (5)

BRD-NMe (5) ^{13}C NMR ($CDCl_3$)

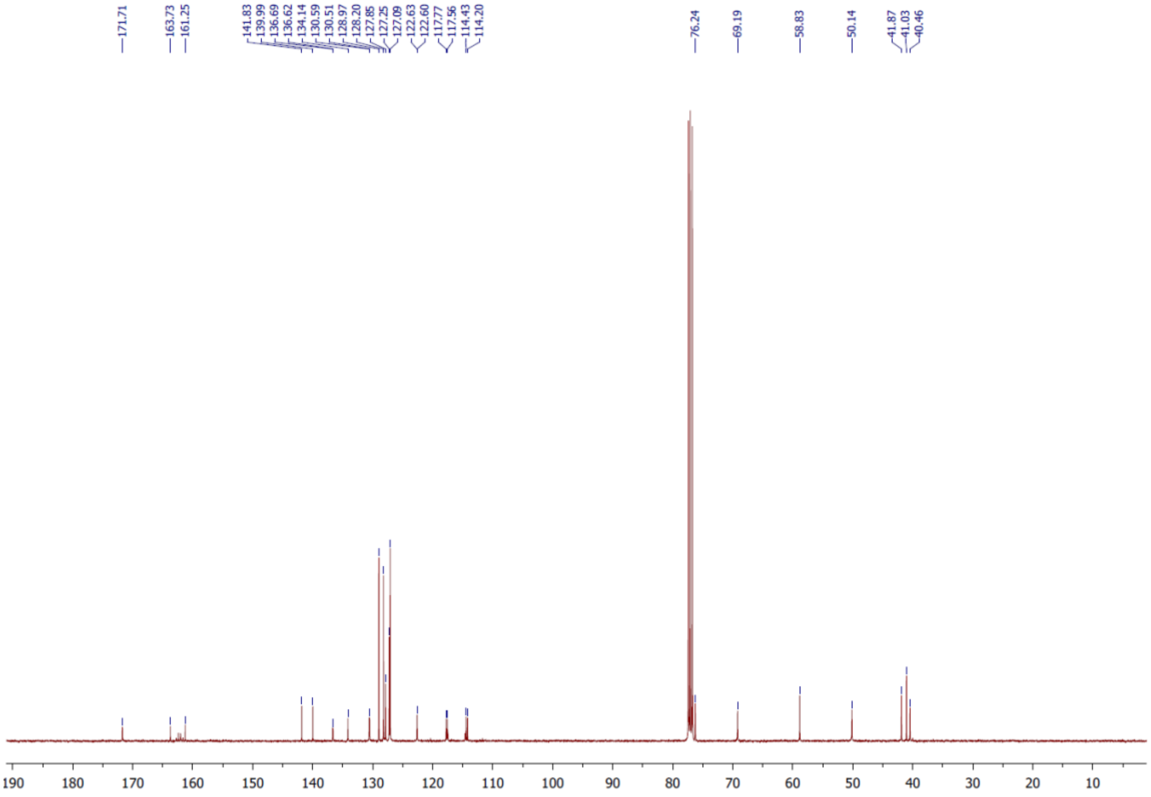

BRD-NOMe (6) ^{1}H NMR ($CDCl_3$)

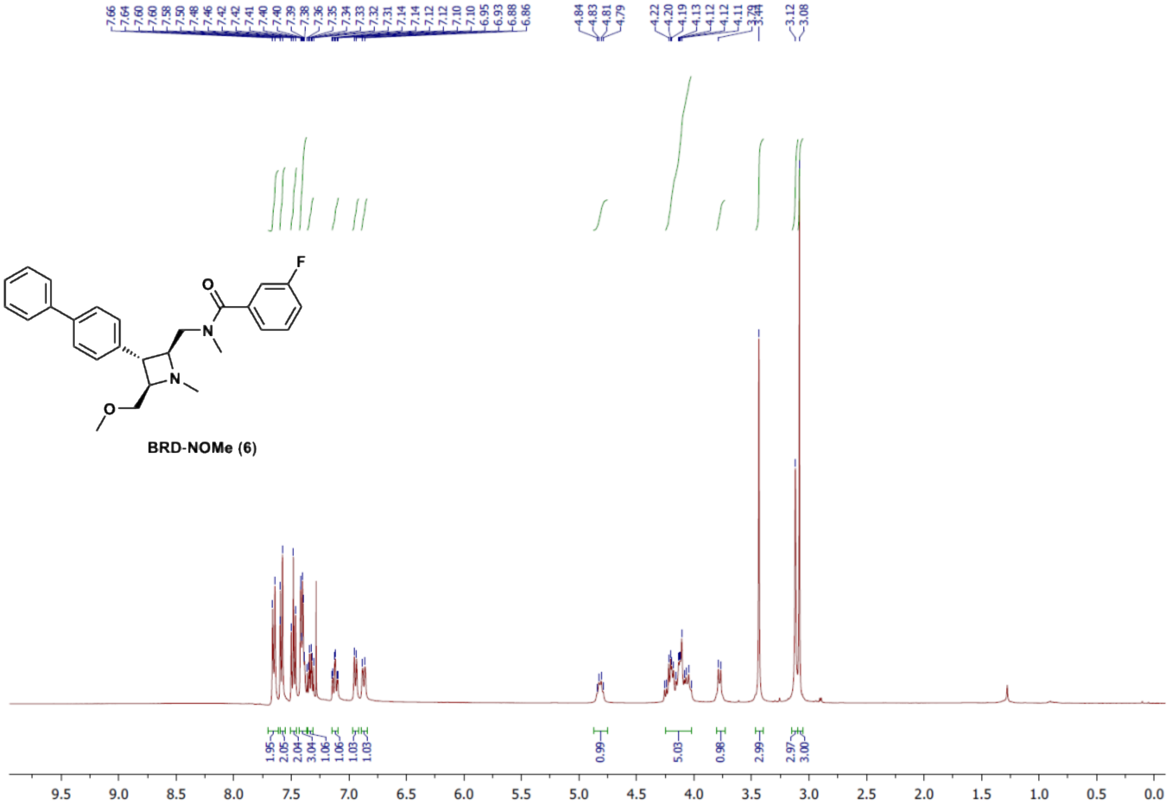

BRD-NOMe (6) ^{13}C NMR ($CDCl_3$)

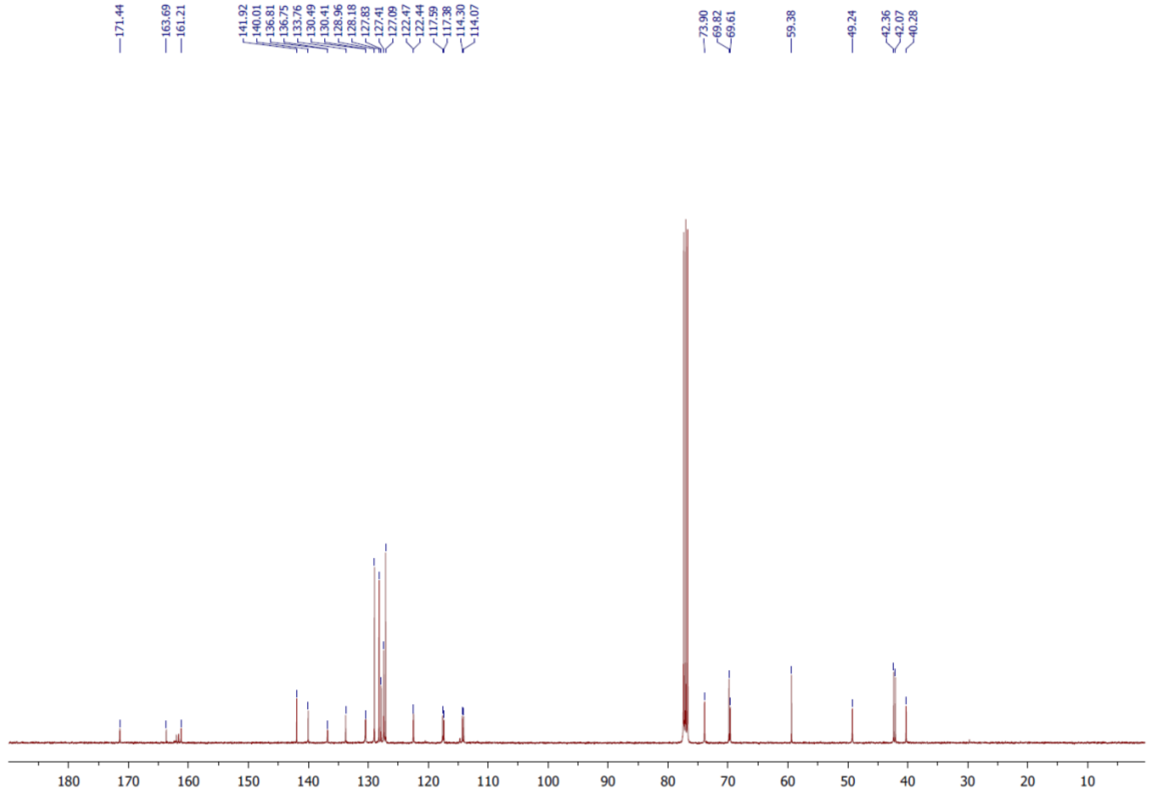

BRD-alkyne (7) ^{1}H NMR ($CDCl_3$)

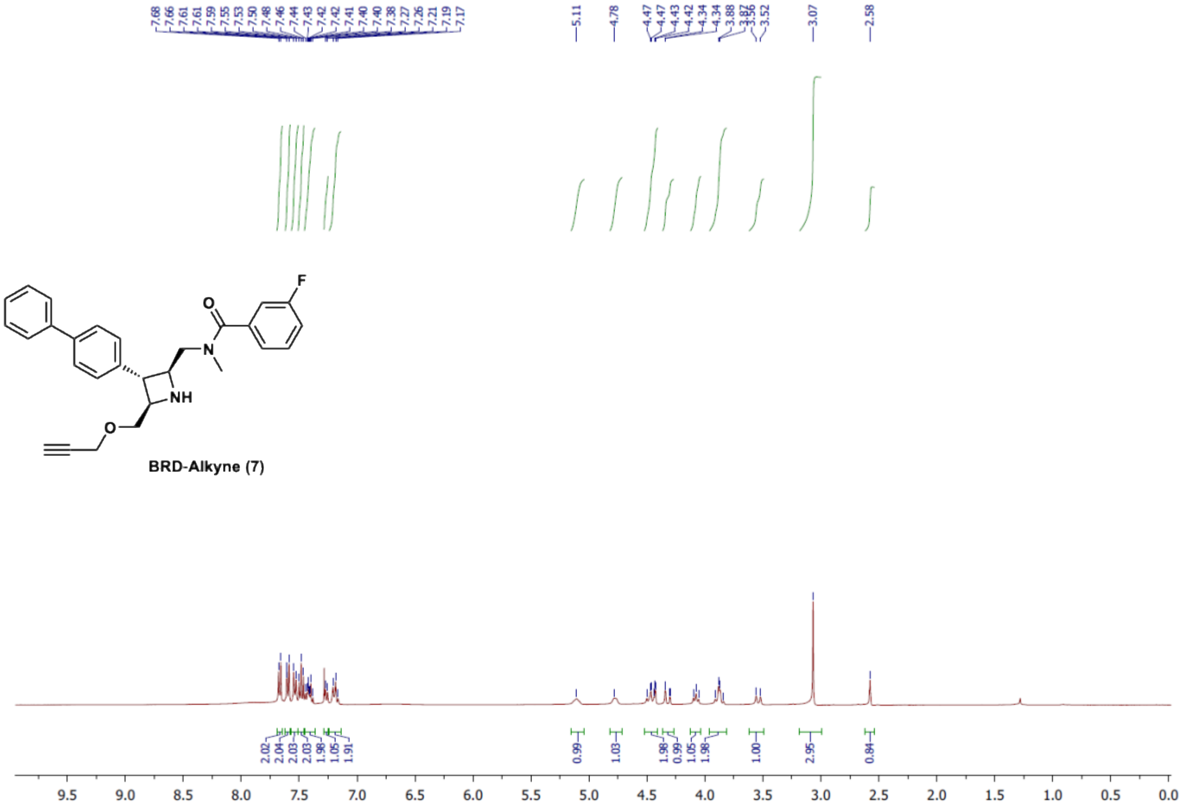

BRD-alkyne (7) ^{13}C NMR ($CDCl_3$)

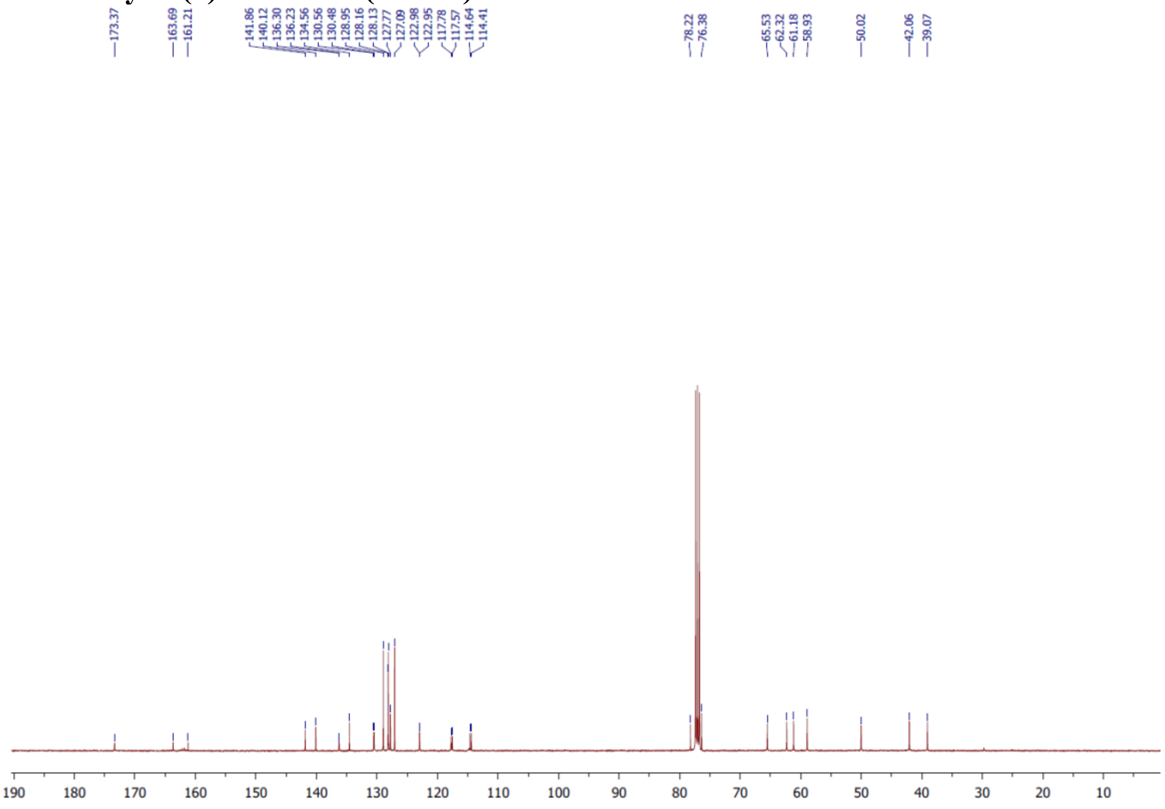

Purchased chemical compounds

Name	Source	Purity
Cycloheximide	Sigma-Aldrich	≥95%
MG132	Sigma-Aldrich	98%
MLN4929	Calbiochem	98%
MLN7243	Chemie Tek	99%

Source Data of Supplementary Information

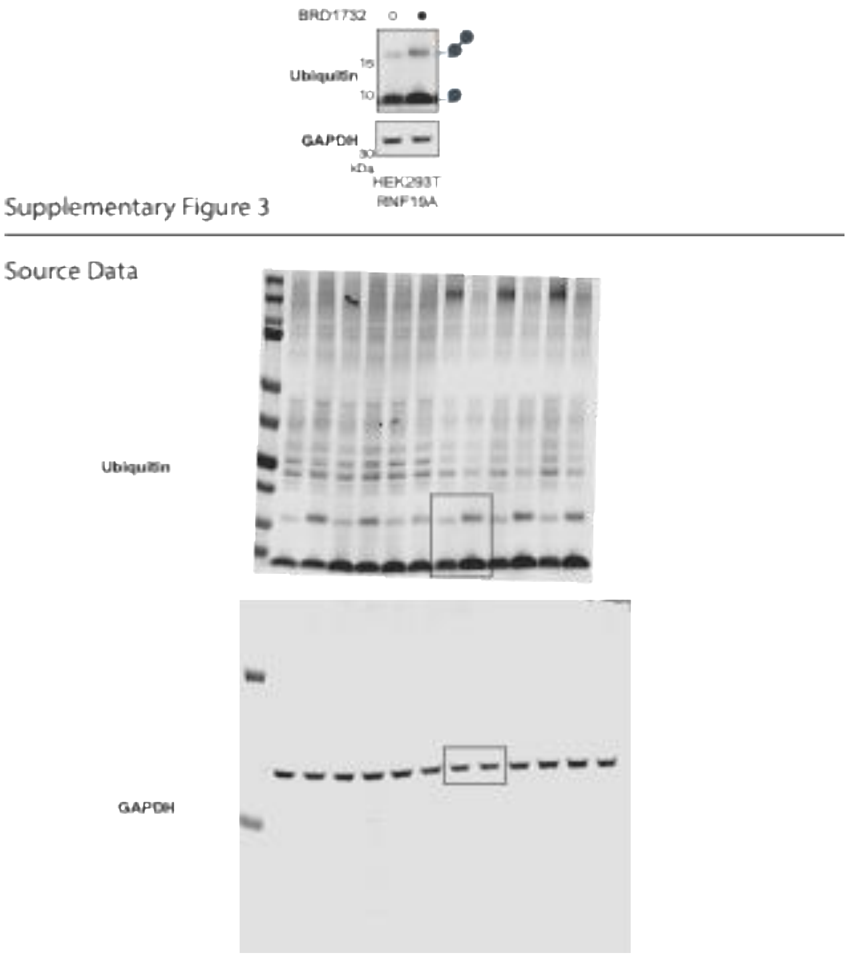

Supplementary Information | Source Data Figure 1. Source data of Supplementary Figure 3.

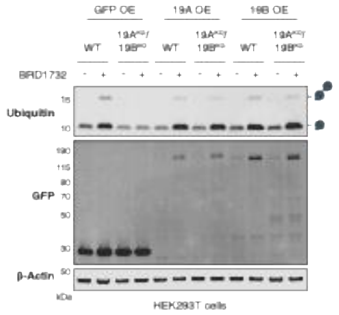

Supplementary Figure 5

Source Data

Ubiquitin

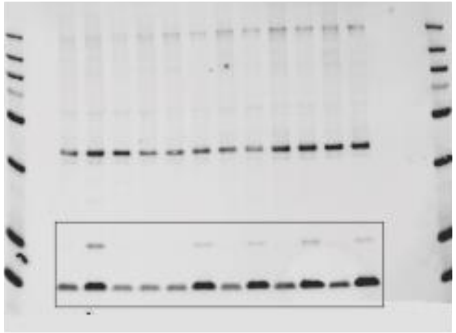

GFP

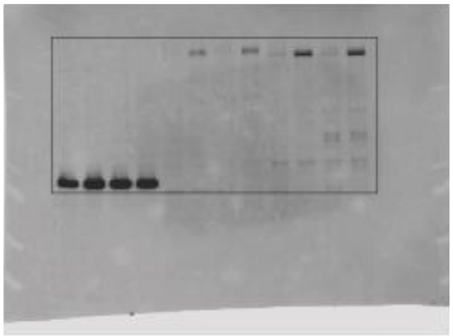

β-Actin

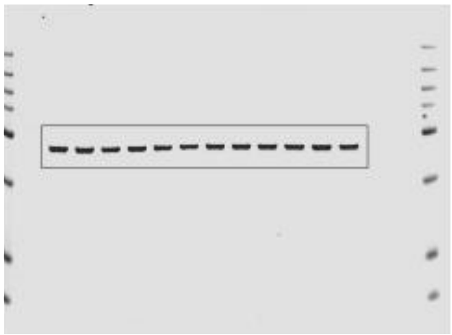

Supplementary Information | Source Data Figure 2. Source data of Supplementary Figure 5.

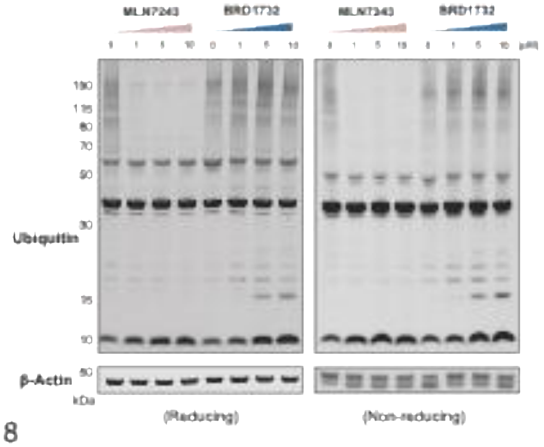

Supplementary Figure 8

Source Data

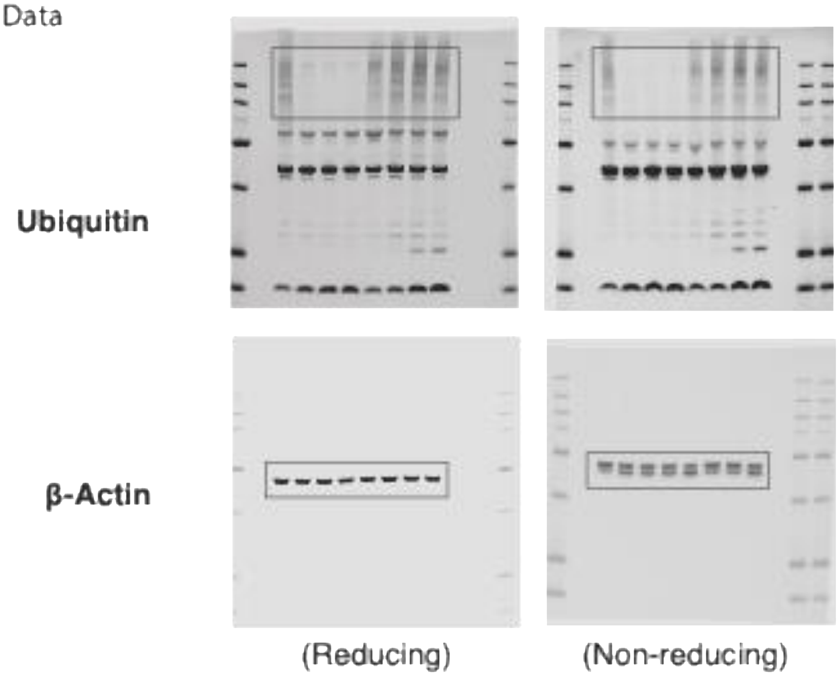

Supplementary Information | Source Data Figure 3. Source data of Supplementary Figure 8.

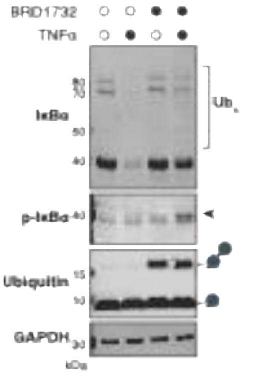

Supplementary Figure 10

Source Data

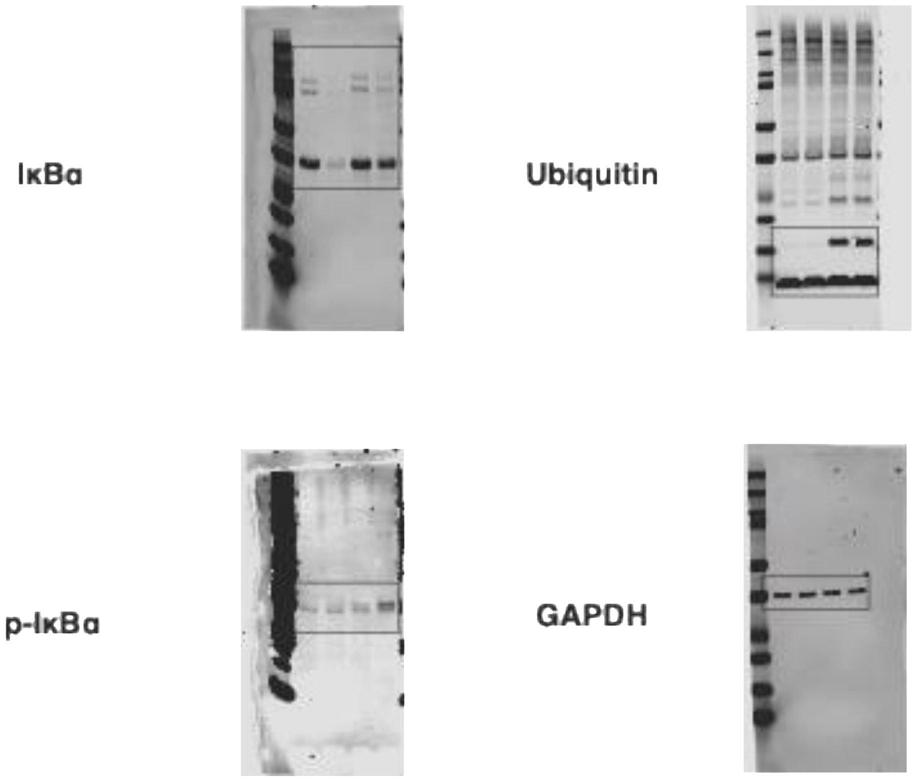

Supplementary Information | Source Data Figure 4. Source data of Supplementary Figure 10.

Citations:

Lowe, J.T.; Lee IV, M.D.; Akella, L.; Davoine, E.; Donckele, E.J.; Durak, L.; Duvall, J.R.; Gerard, B.; Holson, E.B.; Joliton, A.; Kesavan, S.; Lemercier, B.C.; Liu, H.; Marie, J-C.; Mulrooney, C.A.; Muncipinto, M.; Welzel-O'Shea, M.; Panko, L.M.; Rowley, A.; Suh, B-C.; Thomas, M.; Wagner, F.F.; Wei, J.; Foley, M.A.; Marcaurelle, L.A. Synthesis and profiling of a diverse collection of azetidine-based scaffolds for the development of CNS-focused lead-like libraries. *J. Org. Chem*. **2012**, 77, 7187–7211.
