# Supplementary material for: Highly specific intracellular ubiquitination of a small molecule
Source: Nat Chem Biol. 2025 Aug 21;22(4):663–71. doi: 10.1038/s41589-025-02011-1 (PMC13038411; doi:10.1038/s41589-025-02011-1)
Supplement: Supplementary file 2 — Reporting Summary [file 41589_2025_2011_MOESM2_ESM.pdf]

nature portfolio

Corresponding author(s): Jonathan Ostrem

Last updated by author(s): Jun 19, 2025

Reporting Summary

Nature Portfolio wishes to improve the reproducibility of the work that we publish. This form provides structure for consistency and transparency in reporting. For further information on Nature Portfolio policies, see our Editorial Policies and the Editorial Policy Checklist.

Statistics

For all statistical analyses, confirm that the following items are present in the figure legend, table legend, main text, or Methods section.

n/a	Confirmed	
☐	☒	The exact sample size (*n*) for each experimental group/condition, given as a discrete number and unit of measurement
☐	☒	A statement on whether measurements were taken from distinct samples or whether the same sample was measured repeatedly
☐	☒	The statistical test(s) used AND whether they are one- or two-sided<br>*Only common tests should be described solely by name; describe more complex techniques in the Methods section.*
☒	☐	A description of all covariates tested
☐	☒	A description of any assumptions or corrections, such as tests of normality and adjustment for multiple comparisons
☐	☒	A full description of the statistical parameters including central tendency (e.g. means) or other basic estimates (e.g. regression coefficient) AND variation (e.g. standard deviation) or associated estimates of uncertainty (e.g. confidence intervals)
☐	☒	For null hypothesis testing, the test statistic (e.g. *F*, *t*, *r*) with confidence intervals, effect sizes, degrees of freedom and *P* value noted<br>*Give P values as exact values whenever suitable.*
☒	☐	For Bayesian analysis, information on the choice of priors and Markov chain Monte Carlo settings
☐	☒	For hierarchical and complex designs, identification of the appropriate level for tests and full reporting of outcomes
☐	☒	Estimates of effect sizes (e.g. Cohen's *d*, Pearson's *r*), indicating how they were calculated

Our web collection on statistics for biologists contains articles on many of the points above.

Software and code

Policy information about availability of computer code

Data collection	Cell viability assay data were collected using Biotech Gens v3.03. Proteomics data were collected using Xcalibur v4.2. LC-MS data were collected using MassLynx v4.2. Flow Cytometry data were collected using CytExpert v.2.4. Western blot data were collected using Image Studio v5.0.
Data analysis	Cell viability assay data were processed by using GraphPad Prism v.10.2.1. Proteomic analysis in Yamato cells was performed by using Cluster 3.0 and Multiexperiment Viewer. RNA-Seq analysis was performed by using STAR v.2.3.1 and R package DESeq2. Genome-wide CRISPR screening was analyzed by using MAGeCK package. Proteomics data in HCT116 were analyzed by using R package v.3.6.2, rcorr v.0.4.2, Dplyr v.l.0.7, Tidyr v.l.0.0, Reshape2 v.l.4.3, GO.db v.3.10.0, Tibble v.3.1.2, fdrtool v.l.0.12, org.Hs.db v.3.10.0, and Msconvert v.1.1. Flow cytometry data were collected using FlowJo v.10. DeepCoverMOA analysis were using online web server (http://wren.hms.harvard.edu/DeepCoverMOA/). The network analysis were generated using Cytoscape v.3.10.l. Prism data were processed using the code (https://github.com/broadinstitute/repurposing) and R packages v.3.5.l. The LC-MS data were processed by using Masslynx v.4.2.

For manuscripts utilizing custom algorithms or software that are central to the research but not yet described in published literature, software must be made available to editors and reviewers. We strongly encourage code deposition in a community repository (e.g. GitHub). See the Nature Portfolio guidelines for submitting code & software for further information.

Data

Policy information about availability of data

All manuscripts must include a data availability statement. This statement should provide the following information, where applicable:

- Accession codes, unique identifiers, or web links for publicly available datasets
- A description of any restrictions on data availability
- For clinical datasets or third party data, please ensure that the statement adheres to our policy

There are no restrictions on the availability of the data. Proteome quantification data are available in the PRIDE repository (PXD053243 and PXD065168).

Research involving human participants, their data, or biological material

Policy information about studies with human participants or human data. See also policy information about sex, gender (identity/presentation), and sexual orientation and race, ethnicity and racism.

Reporting on sex and gender	N/A
Reporting on race, ethnicity, or other socially relevant groupings	N/A
Population characteristics	N/A
Recruitment	N/A
Ethics oversight	N/A

Note that full information on the approval of the study protocol must also be provided in the manuscript.

Field-specific reporting

Please select the one below that is the best fit for your research. If you are not sure, read the appropriate sections before making your selection.

☒ Life sciences ☐ Behavioural & social sciences ☐ Ecological, evolutionary & environmental sciences

For a reference copy of the document with all sections, see nature.com/documents/nr-reporting-summary-flat.pdf

Life sciences study design

All studies must disclose on these points even when the disclosure is negative.

Sample size	CRISPR, quantitative proteomics, and RNAseq were performed in technical triplicate and cell viability and flow cytometry experiments were performed in biological triplicate. Samples sizes were chosen based on common standards in the field. (Slabicki et al 2020 Nature, doi.org/10.1038/s41586-020-2374-x)
Data exclusions	No data were excluded.
Replication	Multiple replicates were performed for all experiments. Number of replicates are noted in figures by individual dots, and/or specified in figure legends.
Randomization	Randomization was not performed in this study. Media conditions were standardized and multiple biological replicates were utilized to ensure consistency and control for confounding variables.
Blinding	Blinding was not utilized in this study as data collected were empirical and not dependent upon interpretation by the researcher.

Reporting for specific materials, systems and methods

We require information from authors about some types of materials, experimental systems and methods used in many studies. Here, indicate whether each material, system or method listed is relevant to your study. If you are not sure if a list item applies to your research, read the appropriate section before selecting a response.

Materials & experimental systems

n/a	Involved in the study
☐	☒ Antibodies
☐	☒ Eukaryotic cell lines
☒	☐ Palaeontology and archaeology
☒	☐ Animals and other organisms
☒	☐ Clinical data
☒	☐ Dual use research of concern
☒	☐ Plants

Methods

n/a	Involved in the study
☒	☐ ChIP-seq
☐	☒ Flow cytometry
☒	☐ MRI-based neuroimaging

Antibodies

Antibodies used

20 antibodies were used in this study. The antibodies that were purchased from Cell Signaling:
1. anti-β-actin (Cat.4970S/3700S, 1:1000 for WB, Lot#19/21),
2. anti-HA conjugated with Alexa Fluor® 750 (Cat.20818S, 1:500 for WB, 210168),
3. anti-Ub-H2A-K119 (Cat.8240S, 1:500 for WB, Lot#9),
4. anti-E2T (Cat.12992S, 1:1000 for WB, Lot#1),
5. anti-E2A/B (Cat.4944S, 1:1000 for WB, Lot#1),
6. anti-UBE2L3 (Cat.3848S, 1:1000 for WB, Lot#1),
7. anti-Mcl-1 (Cat.94296S, 1:1000 for WB, Lot#5),
8. anti-Nrf1 (Cat.8052S, 1:1000 for WB, Lot#5),
9. anti-p21 (Cat.2947S, 1:1000 for WB, Lot#11),
10. anti-p27 (Cat.3686S, 1:1000 for WB, Lot#5),
11. anti-GAPDH (Cat.97166S, 1:1000 for WB, Lot#1),
12. anti-IκBα (Cat.2859T, 1:1000 for WB, Lot#17),
13. anti-phospho IκBα(Cat.2859S, 1:1000 for WB, Lot#25),
14. anti-GFP (Cat.2956S, 1:1000 for WB, Lot#6),
15. anti-p53 (Cat.9282S, 1:1000 for WB, Lot#6);
Antibodies from LICOR :
16. Goat anti-Mouse IgG IRDye® 800CW (Cat.926-68070, 1:10000 for WB,),
17. Goat anti-Rabbit IgG IRDye® 680RD (Cat.926-32211, 1:10000 for WB,);
Antibodies from Invitrogen:
18. anti-Ubiquitin (Cat.13-1600, 1:1000 for WB, lot#YE37371),
19. anti-ZFAND5 (Cat.MA5-26456, 1:1000 for WB, lot#AA4620362),
20. anti-ZFAND6 (Cat.PA5-57083, 1:1000 for WB, lot#ZI4458577).

Validation

All antibodies listed are commercially available and used for western blotting according to the manufacturer recommendations.
Validation by the manufacturers and the corresponding citations are as shown on their website:
1. anti-β-actin (Cat.4970S/3700S)
https://www.cellsignal.com/products/primary-antibodies/b-actin-13e5-rabbit-mab/4970
https://www.cellsignal.com/products/primary-antibodies/b-actin-8h10d10-mouse-mab/3700
2. anti-HA conjugated with Alexa Fluor® 750 (Cat.20818S),
https://www.cellsignal.com/products/antibody-conjugates/ha-tag-c29f4-rabbit-mab-alexa-fluor-750-conjugate/20818
3. anti-Ub-H2A-K119 (Cat.8240S),
https://www.cellsignal.com/products/primary-antibodies/ubiquityl-histone-h2a-lys119-d27c4-xp-rabbit-mab/8240
4. anti-E2T (Cat.12992S),
https://www.cellsignal.com/products/primary-antibodies/ube2t-d2l7h-rabbit-mab/12992
5. anti-E2A/B (Cat.4944S),
https://www.cellsignal.com/products/primary-antibodies/hr6a-hr6b-antibody/4944
6. anti-UBE2L3 (Cat.3848S),
https://www.cellsignal.com/products/primary-antibodies/ube2l3-antibody/3848
7. anti-Mcl-1 (Cat.94296S),
https://www.cellsignal.com/products/primary-antibodies/mcl-1-d2w9e-rabbit-mab/94296
8. anti-Nrf1 (Cat.8052S),
https://www.cellsignal.com/products/primary-antibodies/tcf11-nrf1-d5b10-rabbit-mab/8052
9. anti-p21 (Cat.2947S),
https://www.cellsignal.com/products/primary-antibodies/p21-waf1-cip1-12d1-rabbit-mab/2947
10. anti-p27 (Cat.3686S),
https://www.cellsignal.com/products/primary-antibodies/p27-kip1-d69c12-xp-rabbit-mab/3686
11. anti-GAPDH (Cat.97166S),
https://www.cellsignal.com/products/primary-antibodies/gapdh-d4c6r-mouse-mab/97166
12. anti-IκBα (Cat.2859T),
https://www.cellsignal.com/products/primary-antibodies/phospho-ikba-ser32-14d4-rabbit-mab/2859
13. anti-phospho IκBα(Cat.2859S),
https://www.cellsignal.com/products/primary-antibodies/phospho-ikba-ser32-14d4-rabbit-mab/2859
14. anti-GFP (Cat.2956S),
https://www.cellsignal.com/products/primary-antibodies/gfp-d5-1-rabbit-mab/2956
15. anti-p53 (Cat.9282S);
https://www.cellsignal.com/products/primary-antibodies/p53-antibody/9282
Antibodies from Invitrogen:

18. anti-Ubiquitin (Cat.13-1600),
https://www.thermofisher.com/antibody/product/Ubiquitin-Antibody-clone-Ubi-1-Monoclonal/13-1600
19. anti-ZFAND5 (Cat.MA5-26456),
https://www.thermofisher.com/antibody/product/ZFAND5-Antibody-clone-OTI9F2-Monoclonal/MA5-26456
20. anti-ZFAND6 (Cat.PA5-57083).
https://www.thermofisher.com/antibody/product/ZFAND6-Antibody-Polyclonal/PA5-57083

Eukaryotic cell lines

Policy information about cell lines and Sex and Gender in Research

Cell line source(s)	HEK293T cells (female, human) were purchased from ATCC<br>MM.1S cells (female, human) were purchased from ATCC<br>PC9 cells (male, human) were purchased from ATCC<br>HCT116 cells (male, human) were obtained from the Broad Institute Genetic Perturbation Platform<br>KP4 cells (male, human) were purchased from JCRB Cell Bank (Japan)<br>HEK293T IKZF3 reporter cells (female, human) were a gift from the laboratory of Benjamin Ebert (DFCI).<br>Yamato-SS cells (male, human) were a gift from Osaka University<br>Expi293F cells (female, human) were purchased from ThermoFisher
Authentication	KP4 and Yamato-SS cell lines were authenticated by standard fingerprinting analyses. All other cell lines were authenticated by commercial vendors.
Mycoplasma contamination	All cell lines tested negative for mycoplasma.
Commonly misidentified lines<br>(See ICLAC register)	HEK293T cells were used due to ease of transient transfection.

Plants

Seed stocks	N/A
Novel plant genotypes	N/A
Authentication	N/A

Flow Cytometry

Plots

Confirm that:

☒ The axis labels state the marker and fluorochrome used (e.g. CD4-FITC).

☒ The axis scales are clearly visible. Include numbers along axes only for bottom left plot of group (a 'group' is an analysis of identical markers).

☒ All plots are contour plots with outliers or pseudocolor plots.

☒ A numerical value for number of cells or percentage (with statistics) is provided.

Methodology

Sample preparation	Cells were detached from plate and washed with PBS. Where relevant, cells were fixed with 4% formaldehyde, permeabilized with methanol, then stained with detection reagents or antibodies. Cells were then resuspended in PBS with 1% FBS and analyzed by flow cytometry.
Instrument	CytoFLEX S flow cytometer
Software	CytExpert, FlowJo
Cell population abundance	Flow cytometry was performed only on isogenic cell lines. Singlet cells were gated by FSC-H and FSC-A. Live cells were gated using FSC-A and SSC-A.
Gating strategy	Example cell gating is shown in supplementary information. For IKZF3-GFP-IRES-mCherry reporter cells, mCherry histogram

Gating strategy

showed two distinct populations of cells, so gate was set to include only the mCherry positive population. FITC/eGFP gate was set based on DMSO-treated cell sample and applied to all subsequent samples in the experiment.

☒ Tick this box to confirm that a figure exemplifying the gating strategy is provided in the Supplementary Information.
