# Supplementary figures and images for: Highly specific intracellular ubiquitination of a small molecule
Source: Nat Chem Biol. 2025 Aug 21;22(4):663–71. doi: 10.1038/s41589-025-02011-1 (PMC13038411; doi:10.1038/s41589-025-02011-1)

**Figure 2a**

(Left panel)

**Ubiquitin**

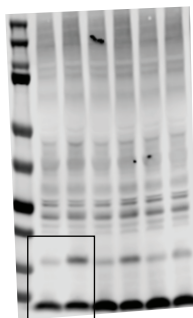

(Right panel)

**Ubiquitin**

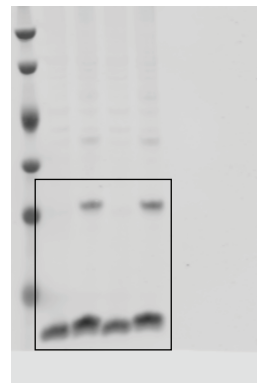

**GAPDH**

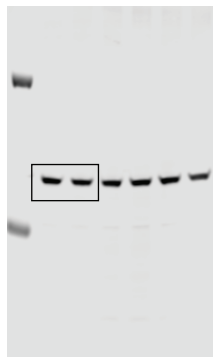

Supplement: Supplementary file 8 — Unprocessed western blots. [file 41589_2025_2011_MOESM8_ESM.pdf]

Figure 3b

Ubiquitin

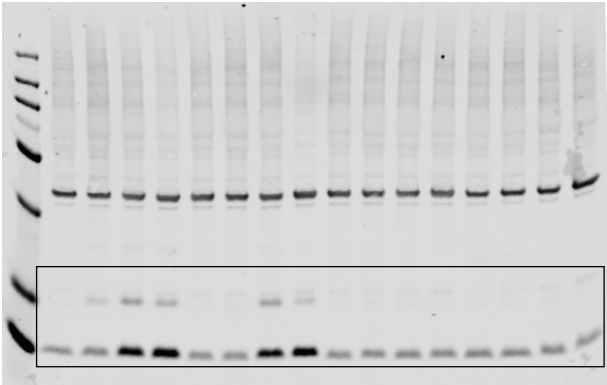

$\beta$ -Actin

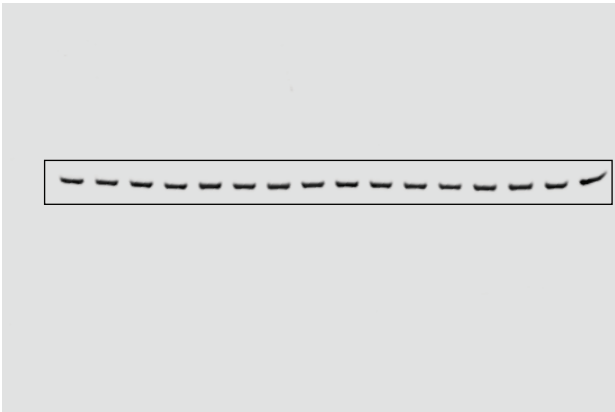

Supplement: Supplementary file 9 — Unprocessed western blots. [file 41589_2025_2011_MOESM9_ESM.pdf]

**Figure 4a**

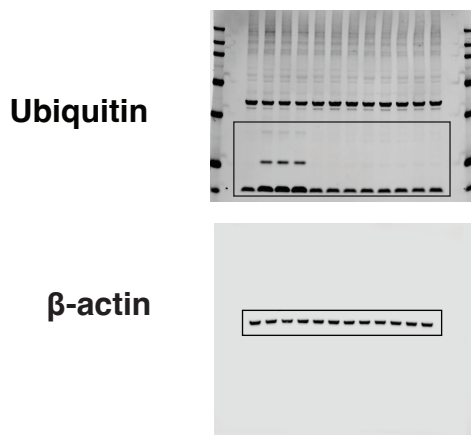

**Figure 4b**

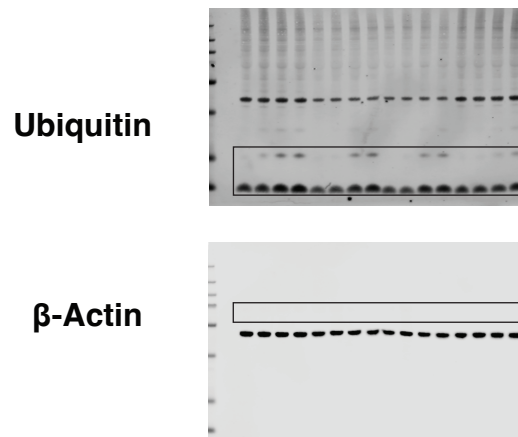

**Figure 4d**

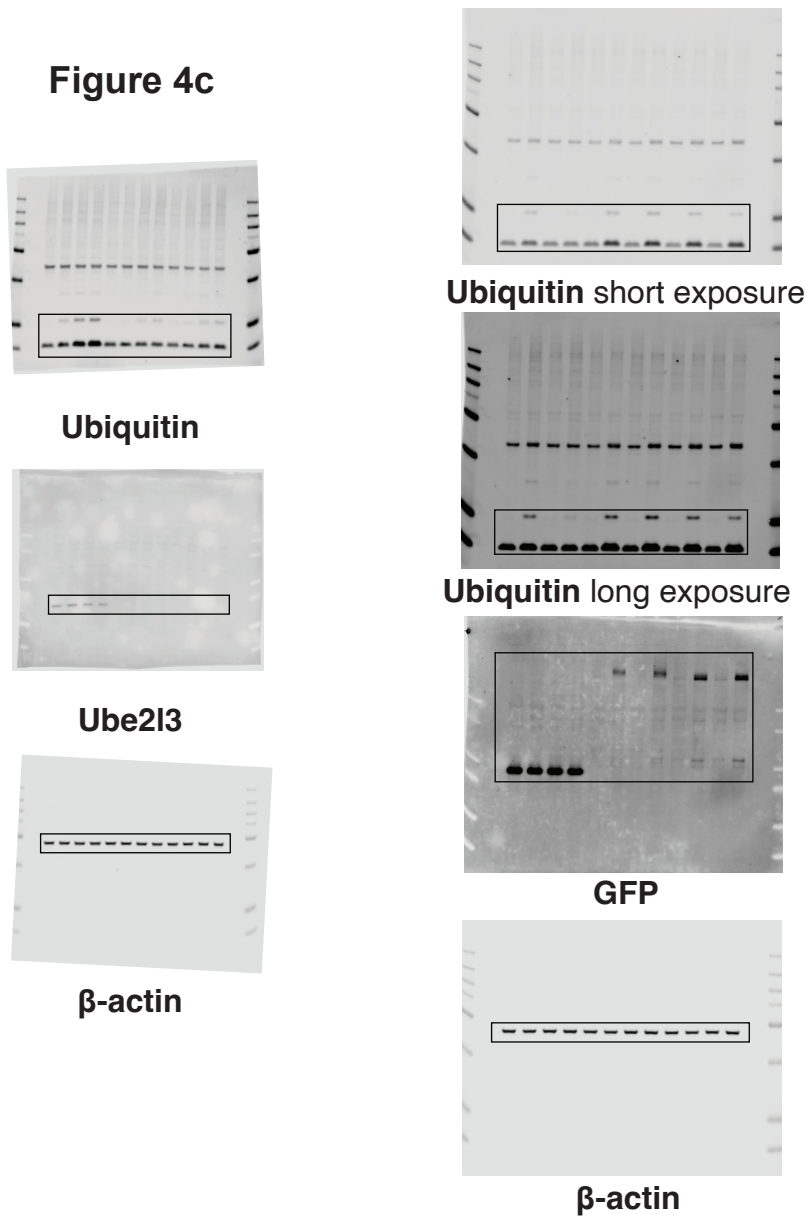

**Figure 4e**

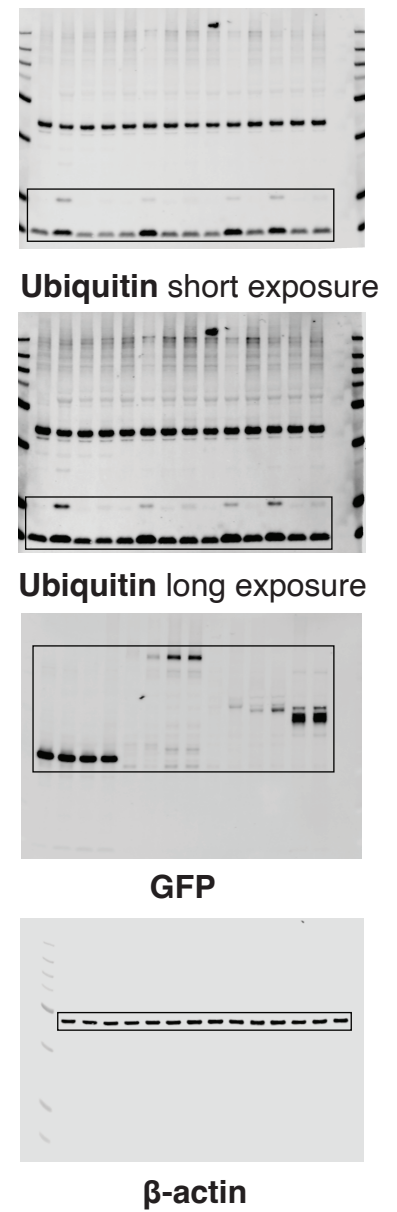

Supplement: Supplementary file 10 — Unprocessed western blots. [file 41589_2025_2011_MOESM10_ESM.pdf]

Figure 5a

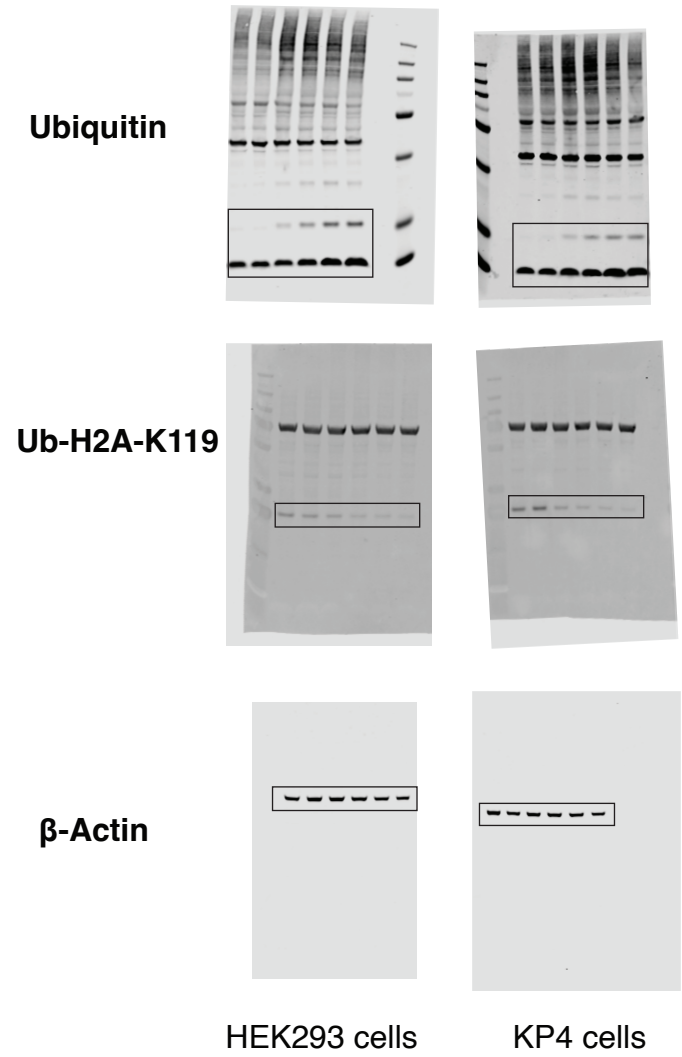

Figure 5e

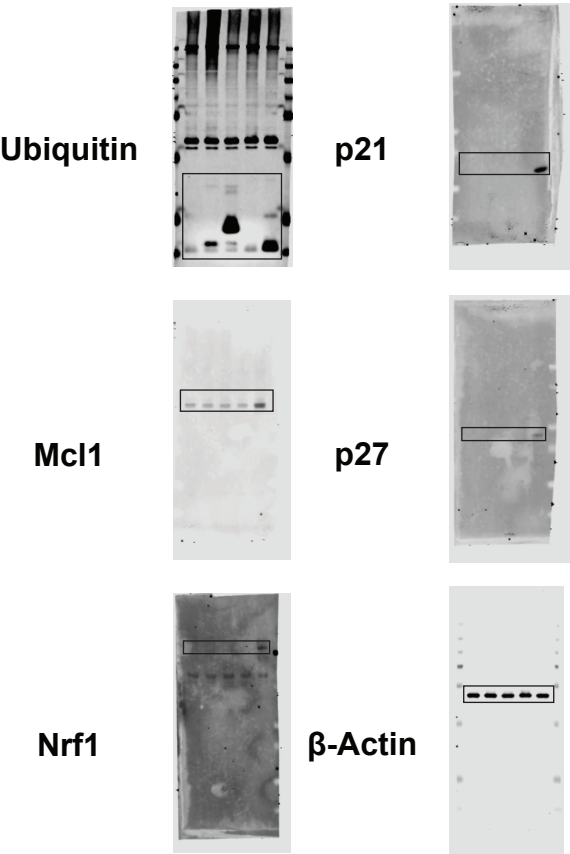

Figure 5c

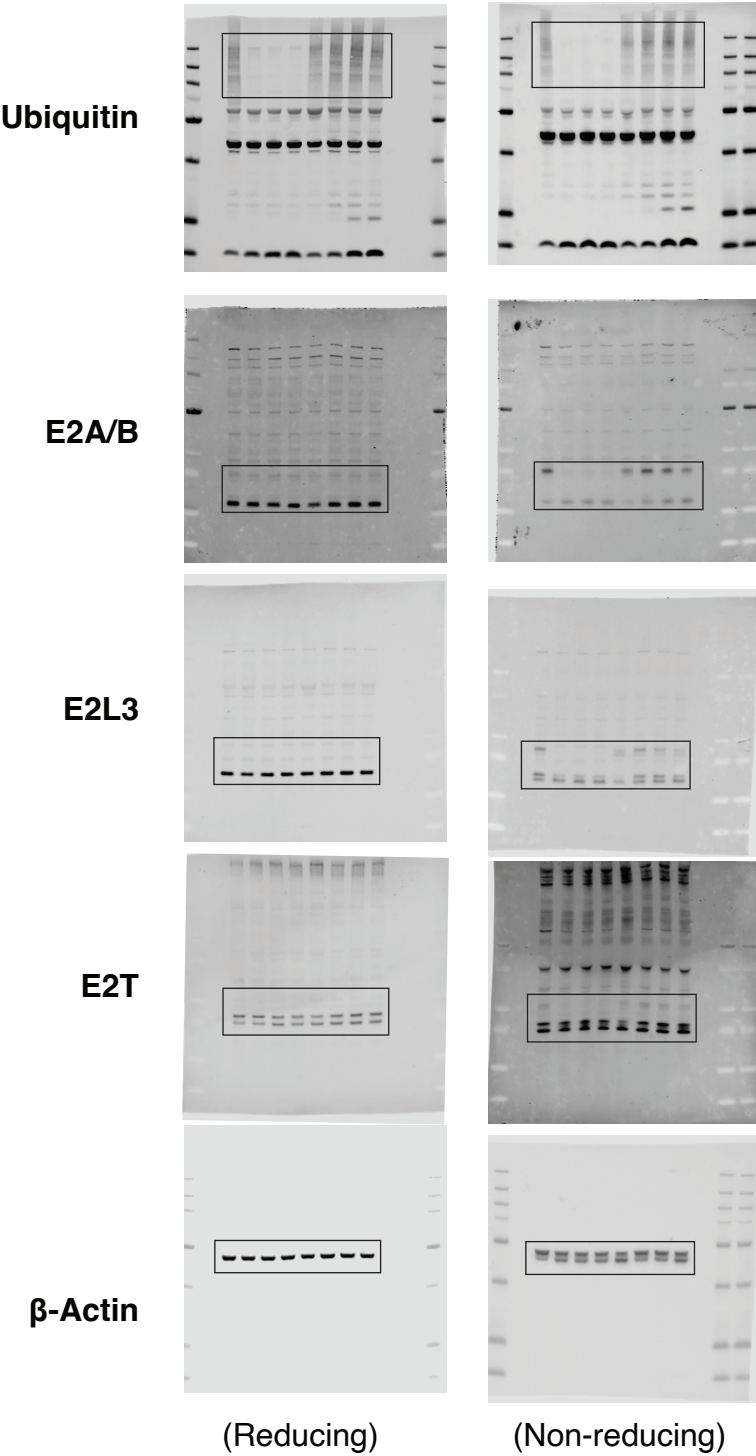

Supplement: Supplementary file 11 — Unprocessed western blots. [file 41589_2025_2011_MOESM11_ESM.pdf]

**Figure 6d**

**Ubiquitin**

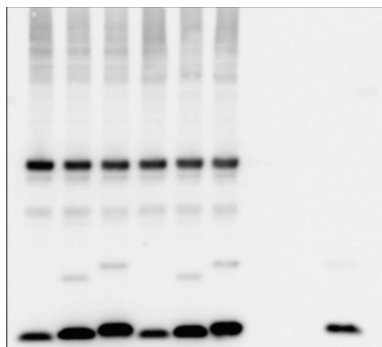

**K27-Ub**

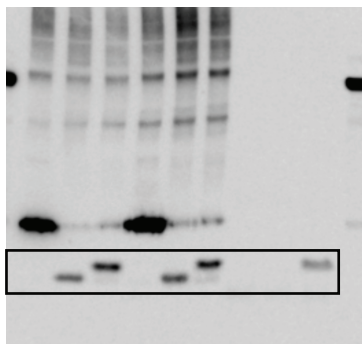

**$\beta$ -Actin**

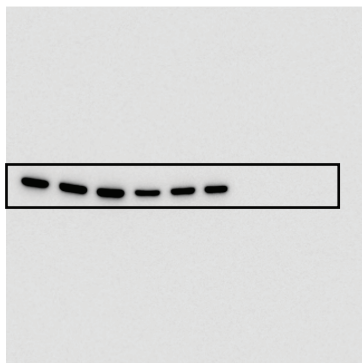

**Figure 6f**

**I $\kappa$ B $\alpha$**

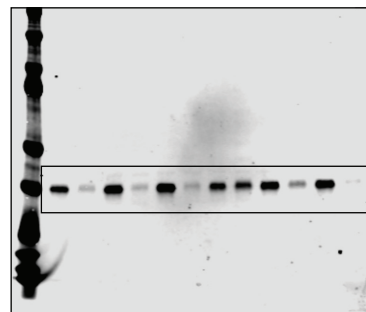

**Ubiquitin**

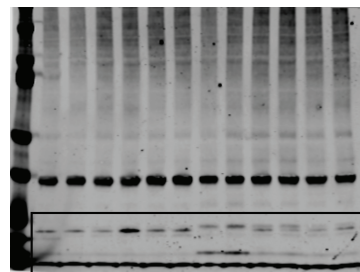

**GAPDH**

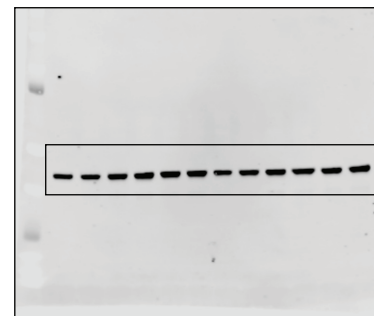

Supplement: Supplementary file 12 — Unprocessed western blots. [file 41589_2025_2011_MOESM12_ESM.pdf]

Extended Data Fig. 1a

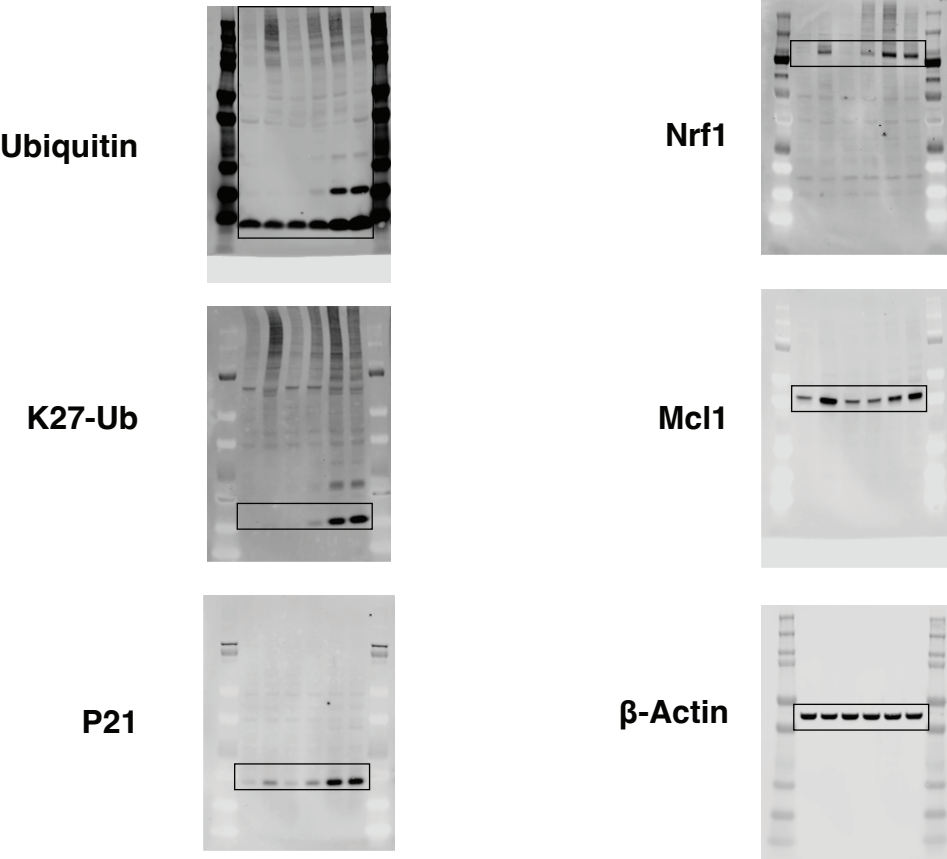

Extended Data Fig. 1b

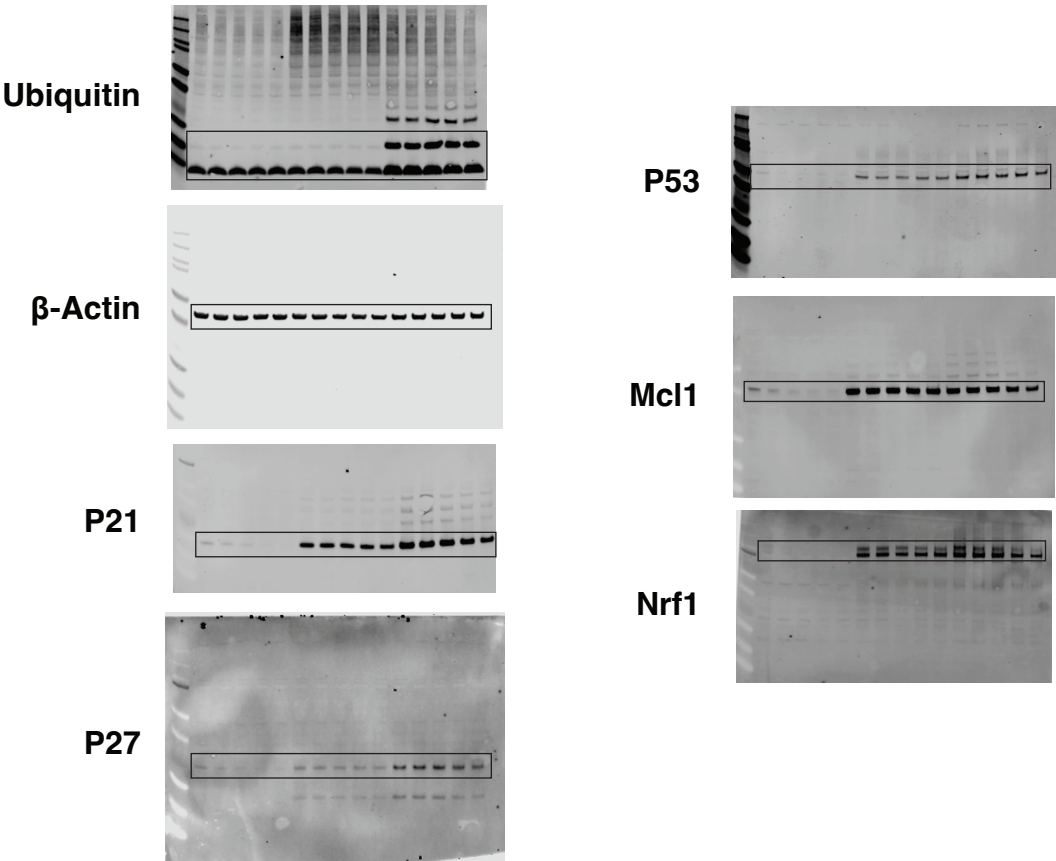

Supplement: Supplementary file 13 — Unprocessed western blots. [file 41589_2025_2011_MOESM13_ESM.pdf]

Extended Data Fig 2b

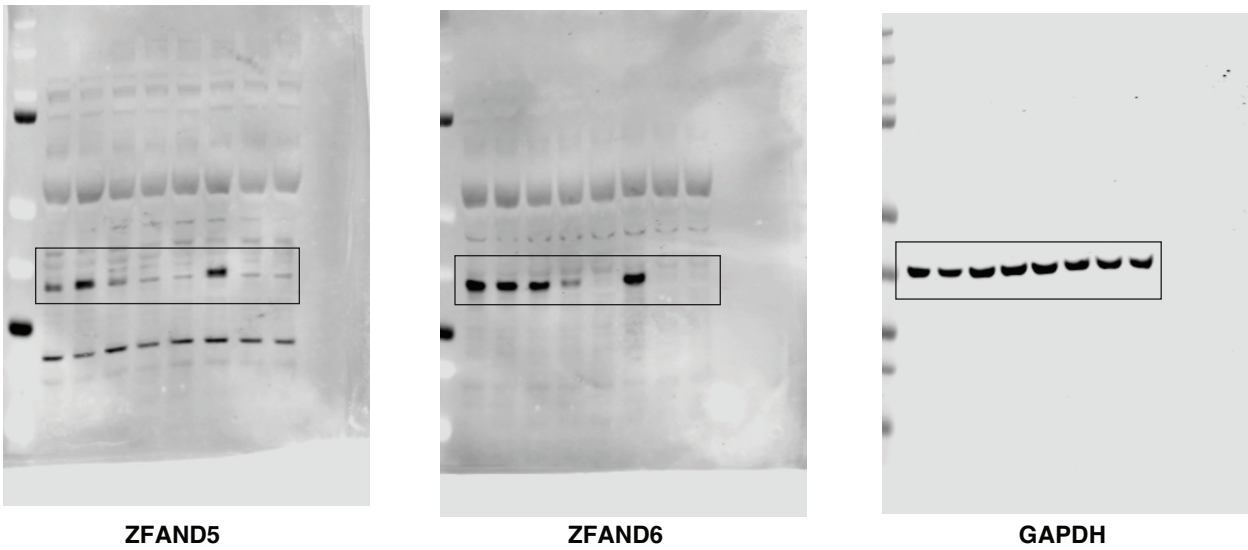

Extended Data Fig 2c

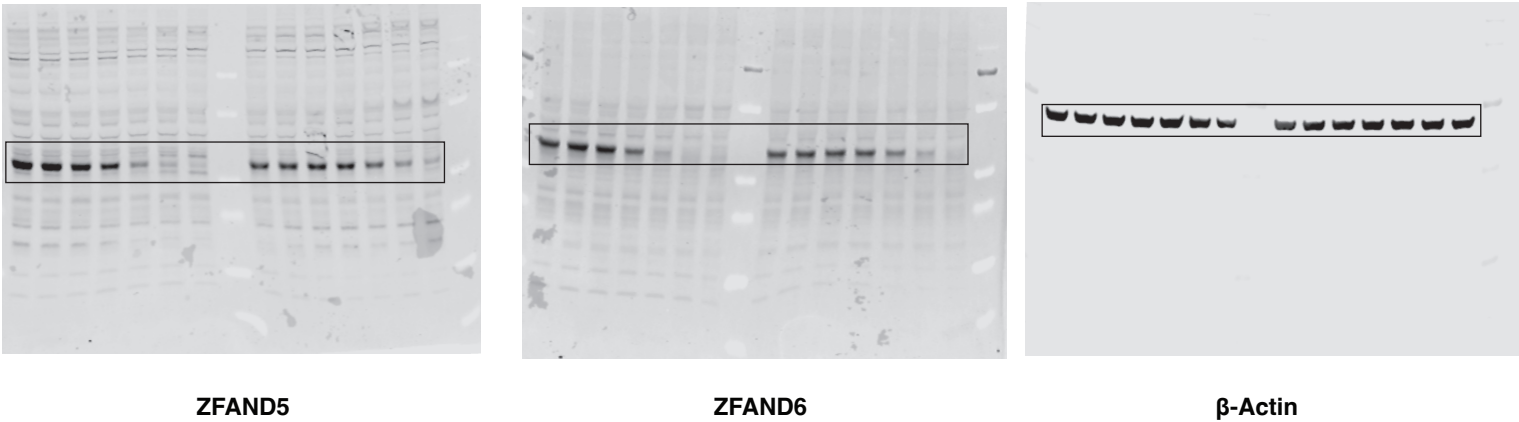

Supplement: Supplementary file 14 — Unprocessed western blots. [file 41589_2025_2011_MOESM14_ESM.pdf]
